# SLC25A4^high^ Hepatocytes Enhance Lifestyle Intervention Response in MASLD by Recruiting Eating Macrophages

**DOI:** 10.1002/advs.78004

**Published:** 2026-10-15

**Authors:** Yiting Qian, Linying Lai, Yu Zhang, Yan Meng, Ziping Song, Baoyuan Huang, Guisheng Song, Bo Sun, Wenzhuo Yang

**Affiliations:** ^1^ Department of Gastroenterology and Hepatology Institute of Digestive Disease, Tongji Hospital, School of Medicine Tongji University Shanghai People's Republic of China; ^2^ Department of Clinical Medicine Suzhou Vocational Health College Suzhou People's Republic of China; ^3^ Department of Gastroenterology, Shanghai East Hospital, School of Medicine Tongji University Shanghai People's Republic of China; ^4^ Department of Medicine University of Minnesota Medical School Minneapolis Minnesota USA; ^5^ School of Pharmacy Hubei University of Chinese Medicine Wuhan People's Republic of China; ^6^ Department of Gastroenterology, Xiang'an Hospital of Xiamen University, School of Medicine Xiamen University Xiamen People's Republic of China

**Keywords:** Exercise, Macrophage, MASLD, PANX1, SLC25A4

## Abstract

While lifestyle interventions (LSI) are the cornerstone of metabolic dysfunction‐associated steatotic liver disease (MASLD) management, the molecular mechanisms driving the histological and metabolic regression in LSI‐responsive patients remain unclear. C57BL/6N mice were hydrodynamically injected with *Slc25a4* and *Sleeping Beauty transposon*. Single‐cell RNA sequencing (scRNA‐seq) profiled the hepatic immune landscape. Metabolomics and co‐culture assays analyzed intercellular crosstalk. Responsive MASLD patients had high *SLC25A4* expression, correlating with exercise‐induced hepatic steatosis improvement. *Slc25a4* overexpression augmented exercise effects. scRNA‐seq identified a distinct population of *Clec4f^+^Timd4^+^P2ry12^+^* macrophages, defined as lipid‐eating macrophages, in livers of *Slc25a4*‐overexpressing mice. Macrophage depletion reinstated hepatosteatosis in high‐fat, high‐fructose, and high‐cholesterol diet‐fed exercised mice with *Slc25a4* overexpression. Mechanistically, *SLC25A4* mediates mitochondrial ATP exchange and selective ATP efflux. Ectopically overexpressed *Slc25a4* activates Pannexin 1 to release ATP, which recruits eating macrophages via P2ry12, clearing lipid‐laden hepatocytes. The GDF15‐PPARα axis between macrophages and hepatocytes was activated. Macrophage *Gdf15* knockdown abrogated *Slc25a4*‐mediated enhanced hepatic fatty acid oxidation (FAO), and hepatic PPARα knockdown abolished this effect as well. Slc25a4 orchestrates an LSI‐responsive hepatocyte–macrophage axis: Pannexin 1‐mediated ATP release recruits lipid‐eating macrophages, whose GDF15 activates PPARα‐driven FAO, establishing Slc25a4 as a therapeutic target to restore exercise response in MASLD.

## Graphical abstract

SLC25A4^high^ hepatocytes drive metabolic‐associated steatotic liver disease (MASLD) remission by recruiting lipid‐eating macrophages. The hepatocyte Slc25a4/PANX1/ATP/P2ry12 axis mediates find‐me signaling that triggers macrophage recruitment and efferocytosis, while recruited macrophages secrete GDF15 to activate hepatocyte PPARα and promote fatty acid oxidation, establishing a reciprocal hepatocyte‐macrophage crosstalk axis that enhances lifestyle intervention responsiveness.

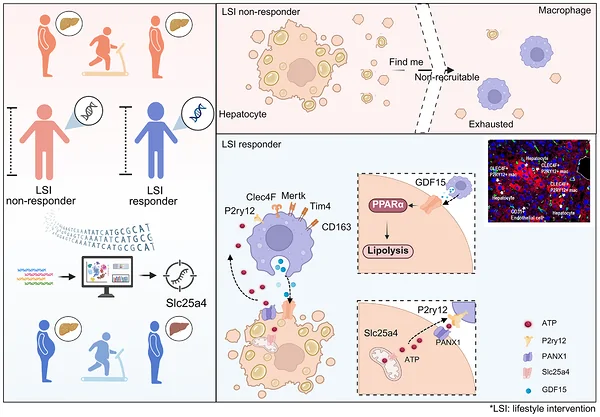

## 1 Introduction

Metabolic dysfunction‐associated steatotic liver disease (MASLD) is the most prevalent chronic liver disease globally, with progressive cases (metabolic dysfunction‐associated steatohepatitis, MASH) carrying a high risk of fibrosis and cirrhosis [1, 2]. Despite extensive pharmacological research, lifestyle intervention (LSI)—particularly weight loss through exercise—remains the definitive first‐line therapy capable of driving profound hepatic metabolic remodeling [3, 4, 5]. However, the precise intracellular signaling networks that successfully orchestrate this exercise‐induced regression remain incompletely elucidated. Intriguingly, among MASH patients who strictly adhere to LSI and achieve comparable weight loss (≥ 5%), clinical responses vary significantly. While a subset exhibits robust histological and metabolic improvements (LSI‐responsive), others remain refractory (LSI‐nonresponsive) [6, 7]. Crucially, this striking clinical divergence serves as an unprecedented window to identify the core genetic determinants that dictate exercise efficacy. By systematically contrasting the transcriptomic landscapes of responsive and non‐responsive cohorts to identify key exercise‐sensitizing genes, we pinpointed solute carrier family 25 member 4 (*SLC25A4)* as a premier candidate driving this beneficial hepatic remodeling.

SLC25A4 encodes the adenine nucleotide translocator 1 (ANT1), a key protein of the inner mitochondrial membrane [8, 9, 10]. Its primary role is to catalyze the exchange of mitochondrial ATP for cytosolic ADP, thereby sustaining oxidative phosphorylation and cellular energy supply [11]. Beyond its transport function, SLC25A4 is a critical regulator of the mitochondrial permeability transition pore (mPTP), which is essential for maintaining mitochondrial membrane potential and controlling cell apoptosis [12, 13]. Dysregulation of SLC25A4 has been linked to increased oxidative stress and mitochondrial dysfunction. While its impact is well‐documented in the heart and muscle, its role in hepatic metabolic remodeling is only beginning to be understood.

Hepatic macrophages play a decisive role in MASH regression through their phagocytic capacity, specifically termed efferocytosis [14]. Beyond modulating inflammation, pro‐resolving macrophages actively identify and engulf lipid‐laden or apoptotic hepatocytes [14]. This targeted clearance is essential to prevent secondary necrosis and the subsequent release of pro‐fibrotic danger signals [15, 16]. By efficiently removing lipotoxic debris and promoting tissue debridement, macrophage‐mediated efferocytosis serves as a critical driver of hepatic remodeling and fibrosis resolution [16, 17].

In this study, we identify Slc25a4 as a primary determinant of exercise responsiveness in MASLD. We propose that Slc25a4 flags lipid‐laden hepatocytes, marking them for selective recognition and clearance by macrophages. Using liver‐specific models, we demonstrate that Slc25a4 overexpression orchestrates the targeted removal of steatotic cells and restores hepatic lipid metabolism. Our findings establish Slc25a4 as a critical driver that enables the therapeutic benefits of lifestyle interventions.

## 2 Materials and Methods

### 2.1 Animal Experiments

Healthy eight‐week‐old male C57BL/6N mice were purchased from Beijing Weitong Lihua Experimental Animal Co., Ltd. Mice were randomly assigned based on the experimental objectives. Random numbers were generated using the standard = RAND() function in Microsoft Excel.

To overexpress Slc25a4 in hepatocytes, mice were hydrodynamically injected via the tail vein with 12.5 µg of pSB‐TBG‐Slc25a4 and 0.625 µg of SB100X (Sleeping Beauty). Control mice received 12.5 µg of pSB‐TBG‐Scramble and 0.625 µg of SB100X. Plasmid mixtures were diluted in 2 mL of sterile saline (0.9% NaCl) and filtered through a 0.22 µm membrane. A rapid injection (within 5–7 s) of a volume equivalent to 10% of the mouse's body weight was performed, following the standard hydrodynamic injection (HDI) protocol.

To establish the metabolic dysfunction‐associated steatotic liver disease (MASLD) model, mice were fed a high‐fat, high‐fructose, and high‐cholesterol diet (HFFC, XT310, Xietong Shengwu, China), while control animals received a standard chow diet (CD, 1010001, Xietong Shengwu, China) and a diet‐restricted chow (XTCON50J, Xietong Shengwu, China). Exercise intervention consisted of daily treadmill running at 12 m/min for 60 min/day, 7 days/week, during the final 4 weeks of HFFC feeding.

Carbenoxolone (CBX; 20 mg/kg, Abcam), a PANX1 hemichannel inhibitor, was administered via intraperitoneal injection. Macrophages were depleted in vivo by intraperitoneal injection of 200 µL clodronate liposomes (LipoCLO, 40337ES08, YEASEN), and PBS liposomes (LipoPBS, 40338ES05, YEASEN) were used as control. shRNA vectors were employed to achieve cell‐type‐specific knockdown of target genes, including Pparα in hepatocytes and Gdf15 in hepatic macrophages. For CCR2 blockade, mice received intraperitoneal injections of anti‐CCR2 neutralizing antibody (Bio X Cell, cat. no. BE0457; 20 µg/mouse) or isotype control (Bio X Cell) on the same schedule.

All the mice were housed on a 12 h light/dark cycle at 25°C with ad libitum food and water. All animal experiments were approved by Animal Ethical and Welfare Committee of Tongji Hospital of Tongji University (Ethics No.20230101‐20240630‐DW‐004).

### 2.2 Data Sources

Raw data and clinical information of GSE106737 and GSE83452 on patients were downloaded from the Gene Expression Omnibus (GEO, http://www.ncbi.nlm.nih.gov/geo/) [7, 18]. Although the GEO summary of GSE106737 describes only the Roux‐en‐Y gastric bypass (RYGB) cohort (n = 21), inspection of the full sample metadata revealed that this dataset is a collection comprising the RYGB cohort, a lifestyle intervention (LSI) responder/non‐responder cohort, and additional samples, as also described in the original publication. In the present analysis, only the LSI responder/non‐responder cohort was extracted from GSE106737 and used as the training cohort to identify candidate targets. For GSE83452, which was derived from the longitudinal cohort of the Antwerp University Hospital, patients who underwent bariatric surgery were excluded, and only patients with transcriptomic profiles of paired liver biopsies obtained before and after LSI were retained. GSE83452 served as an independent validation cohort to verify the derived results. All patients were consecutively recruited at the Liver Clinic and Obesity Clinic of the Antwerp University Hospital and underwent hepatologic and metabolic work‐ups. Following a 1‐year lifestyle intervention (LSI), NASH patients were classified as responders if they exhibited improvement in lobular inflammation and/or ballooning, and as non‐responders if no such improvement was observed. Their corresponding detailed histological information is provided in Table S2. Data access was authorized by Springer Nature, permit no.: 6322300929277. All the specimens were collected with informed consent, according to the regulations of the Liver Clinic and Obesity Clinic of the Antwerp University Hospital.

### 2.3 Differential Expression and Gene Set Enrichment Analysis

Data were RMA‐normalized using oligo/bioconductor and corrected for batch effects using SVA/Bioconductor package in R [19]. Differentially expressed genes (DEGs) were screened by the “limma” package, and visualized by the “ggplot2” package [20]. Gene Ontology (GO) enrichment analysis on DEGs was carried out with clusterProfiler package to explore the characteristic function [21]. Ontology gene sets (c5.all.v7.4.symbols) were used for gene set enrichment analysis (GSEA) through the clusterProfiler package. A false discovery rate (FDR) < 0.25 and *P* < 0.05 were considered significant enrichment.

### 2.4 Protein–Protein Interaction Enrichment Analysis

For each given gene list, protein‐protein interaction enrichment analysis (PPI) was carried out with the following databases: STRING, BioGrid, OmniPath, InWeb_IM [22, 23, 24]. Only physical interactions in STRING (physical score > 0.132) and BioGrid were used. The resultant network contains the subset of proteins that form physical interactions with at least one other member in the list. If the network contained between 3 and 500 proteins, the Molecular Complex Detection (MCODE) algorithm was applied to identify densely connected network components [25]. Pathway and process enrichment analysis was applied to each MCODE component independently, and the three best‐scoring terms by p‐value were retained as the functional description of the corresponding components, shown in the tables underneath corresponding network plots.

### 2.5 Weighted Gene Co‐Expression Network Analysis (WGCNA)

We used the goodSamplesGenes method of R software package WGCNA on gene expression matrix to remove outlier genes and samples, and constructed a scale‐free co‐expression network [25]. At first, the Pearson's correlation matrices and average linkage method were both performed for all pair‐wise Genes. Then, a weighted adjacency matrix was constructed using a power function:

$$\;a_{mn}={\left|c_{mn}\right|}^{\beta }$$

(*c_mn_* = Pearson's correlation between Gene_m and Gene_n; *a_mn_* = adjacency between Gene_m and Gene_n; β was a soft‐thresholding parameter that could emphasize strong correlations between Genes and penalize weak correlations)

After choosing the power of 9, the adjacency was transformed into a topological overlap matrix (TOM), which could measure the network connectivity of a Gene defined as the sum of its adjacency with all other Genes for network generation, and the corresponding dissimilarity (1‐TOM) was calculated.

$$\;\mathrm{TO}M_{mn}=d_{mn}+a_{mn}/\min\left(e_{m}+e_{n}\right)+1-a_{mn}$$

(*d_mn_* =  Sum of the adjacency coefficients of the nodes connected by Gene_m and Gene_n; *e* = Sum of the adjacency coefficients of the given gene with all other genes in the weighted network).

To classify Genes with similar expression profiles into Gene modules, average linkage hierarchical clustering was conducted according to the TOM‐based dissimilarity measure with a minimum size (Gene group) of 30 for the Genes dendrogram. To further analyze the module, we calculated the dissimilarity of module eigen Genes, chose a cut line for module dendrogram and merged some module. The dissimilarity of module eigengenes was calculated to merge certain modules (distance less than 0.25). Finally, a total of 4 co‐expression modules were obtained, in which the grey module was considered as the set of genes that could not be assigned to any module.

For hub gene screening, gene significance (GS) was obtained by calculating the expression correlation with the gene [26]. Module membership (mm) was obtained by calculating the expression correlation of module eigenvectors with genes. Based on the cut‐off criteria (|MM| > 0.76 and |GS| > 0.6), 13 genes with high connectivity in the clinical significant module were identified as hub genes.

### 2.6 Human Liver Specimens

To validate the expression pattern and clinical relevance of SLC25A4 in our in‐house cohort, liver biopsy samples were collected from patients diagnosed with MASLD at Tongji Hospital of Tongji University between 2014 and 2023. Patients were followed up for their response to exercise intervention, and FibroScan results before and after the intervention were recorded. A total of 9 patients pathologically diagnosed with fatty liver disease were included, after excluding secondary causes including alcohol intake and drug usage. Based on FibroScan‐derived controlled attenuation parameter (CAP) values, a scatter plot depicting CAP change rate versus Slc25a4 fluorescence intensity was created. The association between these continuous variables was evaluated using Pearson's correlation coefficient. All clinical procedures were approved by the Ethics Committee of Tongji Hospital of Tongji University (Approval No. 2023‐001). All research was conducted in accordance with both the Declarations of Helsinki and Istanbul. Written informed consent was obtained from all participants prior to sample collection.

### 2.7 Single‐Cell RNA Sequencing

Fresh liver tissues were harvested from mice and immediately placed in tissue preservation solution. Samples were transferred on ice to OE Biotech Co., Ltd. (Shanghai, China) for single‐cell isolation, library preparation, and sequencing using the 10x Genomics Chromium platform. Briefly, single‐cell isolation was performed via gentle mechanical dissociation combined with collagenase digestion, followed by filtration through a 70 µm cell strainer to remove tissue debris. Library preparation was conducted using the 10x Genomics Chromium Single Cell 3’ Reagent Kit (v3.1). For data analysis, raw sequencing data were processed using the Cell Ranger (v7.1.0) pipeline for demultiplexing, alignment, and cell barcode filtering. The resulting gene expression matrices were further analyzed with the Seurat package, including quality control, dimensionality reduction (PCA and UMAP), cell clustering, cell type annotation and differential gene expression analysis. Trajectory analysis was performed using Monocle 2 to characterize the progression of cell populations. The raw sequencing data in this study have been deposited in the NCBI Gene Expression Omnibus (GEO) under accession number GSE343644.

### 2.8 RNA Sequencing Analysis

Primary mouse hepatocytes with Slc25a4ʰ^i^ ^g^ʰ mCherry^+^ were sorted from C57BL/6N mice after they had been hydrodynamically injected with pSB‐TBG‐Slc25a4‐mCherry (Slc25a4 mice) or empty vector (Control mice), followed by HFFC diet feeding and exercise intervention, for transcriptomic sequencing. Total RNA was isolated using TRIzol reagent and immediately stored at ‐80°C, and its purity and concentration were measured with a NanoDrop 2000 spectrophotometer (Thermo Scientific, USA). The transcriptome sequencing and related analyses were conducted by Shanghai Bioprofile Technology Company Ltd.

### 2.9 Metabolomics Analysis

The plasmids pLV3‐CMV‐Slc25a4‐Puro and pLV3‐CMV‐MCS‐Puro were purchased from Wuhan Miaoling Biotechnology Co., Ltd. Lentivirus was prepared by using lentivirus packaging system (Takara Bio, USA) and transduction was performed by spin infection. Briefly, virus was added to primary hepatocytes in a 6‐well plate. The plate was sealed and centrifuged at 2,500 rpm for 90 min at room temperature. After 48 h of transduction, primary mouse hepatocyte supernatant samples were collected and immediately stored at ‐80 °C. Before LC‐MS analysis, the samples were thawed on ice and processed to remove proteins. Then samples were detected and analyzed by Shanghai Bioprofile Technology Company Ltd.

### 2.10 Isolation and Culture of Primary Hepatocytes

Primary hepatocytes were isolated from male C57BL/6N mice. Mice were euthanized, and the livers were immediately excised, rinsed in pre‐cooled PBS, and minced into small fragments (∼1 mm^3^) under sterile conditions. The liver tissue was transferred into digestion solution containing 100 units/ml collagenase type IV (Sigma‐Aldrich, USA) in Hank's balanced salt solution (HBSS) and incubated at 37°C for 30 min with gentle agitation. Following digestion, the cell suspension was filtered through a 70 µm nylon mesh and centrifuged at 50 × g for 3 min at 4°C to enrich hepatocytes. The resulting pellet was washed twice with Williams’ E medium (Gibco, USA) supplemented with 10% fetal bovine serum (FBS), 1% penicillin‐streptomycin, and 1% L‐glutamine. Cell viability was assessed by trypan blue exclusion, and hepatocytes with viability >85% were seeded onto collagen I‐coated plates at a density of 1 × 10^6^ cells/well in 6‐well plates. Cells were cultured at 37°C in a 5% CO_2_ incubator, and the medium was changed after 4–6 h to remove non‐adherent cells.

### 2.11 Cell Culture, Migration Assays and ATP Measurement

RAW264.7 (mouse macrophage), AML12 (mouse hepatocyte) and THLE‐2 (human hepatocyte) were purchased from FuHeng Cell Center (Shanghai, China). For macrophage migration, 5 × 10^4^/mL cells were placed on an 8 µm pore Transwell, incubated with supernatants collected from mouse primary hepatocytes overexpressing Slc25a4 in lower chamber, and migration determined by crystal violet staining and ImageJ. Measurement of ATP was performed according to the manufacturer's protocol (Cat. no. S0027; Beyotime Biotechnology).

Monocytes were isolated from mouse peripheral blood using Mouse Peripheral Blood Monocyte Isolation Solution Kit (Cat. no. P5230; Beijing Solarbio Science & Technology Co., Ltd) followed by stimulation with macrophage colony‐stimulating factor (M‐CSF) (Cat. no. M9170; Sigma‐Aldrich) to induce differentiation. Free fatty acids (FFAs) were prepared by mixing oleic acid (OA) and palmitic acid (PA) at a ratio of 2:1, and the mixture was used for the preparation of conditioned medium. THLE‐2 cells were treated with recombinant GDF15 protein (Cat. no. AB50077; Abcam) or a control reagent, and then harvested for analysis of associated protein expression changes.

### 2.12 RNA Isolation and Relative Quantitative PCR

For mRNA detection, RNA extracted by TRIzol method was reverse transcribed according to the manufacturer's protocol (cat. no. R323; Vazyme Biotech Co.,Ltd). Further, real‐time PCR was performed by a QuantStudio 5 Real‐Time PCR System (Applied Biosystems) using SYBR green reagent (cat. no. Q711; Vazyme Biotech Co.,Ltd). β‐actin was used as an internal control and 2^‑ΔΔCq^ method was used for expression quantification. Primers used for quantitative RT‐PCR in the process are listed in Table S1.

### 2.13 Western Blot

Samples were lysed by RIPA lysis buffer (Beyotime Institute of Biotechnology, China) containing 1% phenylmethanesulfonyl fluoride. A total of 30 µg protein was loaded onto 10%, 12.5% SDS‐polyacrylamide gels, and transferred to PVDF membranes, which were incubated overnight at 4°C with primary antibody followed by an incubation with the horseradish peroxidase‑conjugated goat anti‑rabbit IgG secondary antibody (cat. no. A0208; 1:10,000; Beyotime Institute of Biotechnology) and goat anti‑mouse IgG (cat. no. A0216; 1:10 000; Beyotime Institute of Biotechnology) at room temperature for 1 h. Pre‐stained protein markers (WJ103, Epizyme Biotech; RM19001, ABclonal) were loaded alongside the samples to monitor protein migration and transfer efficiency. The protein bands were visualized by ECL Moon from Beyotime Biotechnology and imaged with a chemiluminescence imaging system. The primary antibodies were as follows: anti‑Slc25a4 (cat. no. TD6674; 1:1,000; Abmart), anti‑Panx1 (cat. no. 12595‐1‐AP; 1:1,000; Proteintech), anti‐CPT1α (cat. no. PS19122; 1:1,000; Abmart), anti‐PPARα (cat. no. TA5301; 1:1,000; Abmart), anti‐FASN (cat. no. 66591‐1‐Ig; 1:5,000; Proteintech), anti‐GDF15 (cat. no. 27455‐1‐AP; 1:5,000; Proteintech), anti‐TNFα (cat. no. ab215188; 1:1,000; Abcam) and anti‑β‑actin (cat. no. MA1‑140; 1:10,000; Thermo Fisher Scientific, Inc.). Each target protein was quantified against the corresponding β‐actin loading control from the same membrane/blot.

### 2.14 Biochemical, Histological Assessment and Multiplex Immunohistochemistry (mIHC)

Levels of triglycerides (TG) in the liver tissue or serum were determined using a commercial kit (Nanjing Jiancheng, China) according to the instructions. Liver samples were fixed in 4% paraformaldehyde for 24 h at room temperature. For the preparation of paraffin‐embedded tissues, tissues were routinely dehydrated at room temperature using an increasing ethanol gradient followed by xylene. The tissues were then embedded in paraffin. For the preparation of frozen tissues, tissues were routinely dehydrated in an increasing sucrose gradient, embedded in optimal cutting temperature (OCT) compound, and stored at −80°C. Hematoxylin and eosin (H&E) staining was carried out in liver sections. Cell apoptosis was assessed by TUNEL staining of liver sections using a commercial kit (CX107, Epizyme Biotech, Shanghai, China). Multiplex immunohistochemistry was performed by staining 4‐µm‐thick formalin‐fixed, paraffin‐embedded whole tissue sections with standard primary antibodies sequentially and paired with TSA 7‐color kit (D110071‐50T, WiSeeBio), then by staining with DAPI. The primary antibodies were as follows: Anti‑Slc25a4 (cat. no. TD6674; 1:50; Abmart), Anti‐Clec4f (cat. no. 156803; 1:200; Biolegend), Anti‐CCR2 (cat. no. 16153‐1‐AP; 1:1000; Proteintech), Anti‐CD163 (cat. no. GB15340; 1:1000; Servicebio), Anti‐P2RY12 (cat. no. TD10263; 1:50; Abmart), Anti‐CD31 (cat. no. 77699; 1:1,000; CST), Anti‐F4/80 (cat. no. ab300421, 1:5000; Abcam).

### 2.15 Flow Cytometry

The dissected livers were digested in the Digestion buffer (10 mL Collagenase IV, 1 mg/mL, Sigma) and 5 µL DNase I (100 U/µL, Sigma) at 37°C for 30 min. The cells were poured through 70 µm sterile nylon filter and centrifuged at 50 g for 5 min at 4°C to remove hepatocytes. The supernatant was centrifuged at 400 g for 5 min at 4°C, washed three times with 2% FBS PBS buffer, and then subjected to flow cytometry analysis. Antibodies were purchased from Biolegend and Thermo Fisher Scientific. The following markers and clones were used based on the project objectives: Bodipy, CCR2, F4/80, Clec4f, and Tim4. Cell suspension was stained with appropriate antibodies for 30 min on ice. Stained cells were then suspended in an appropriate volume of fluorescence‐activated cell sorting (FACS) buffer (PBS with 2% BSA). Data were acquired on BD LSR Fortessa and analyzed with FlowJo Software.

### 2.16 Statistical Analysis

Statistical analyses were performed using R version 4.3.2 (R Foundation for Statistical Computing, Vienna, Austria) and GraphPad Prism (version 8.0, GraphPad Software, Boston, MA, USA). One‐way analysis of variance was used for comparison of three or more groups, and after one‐way analysis, Tukey's test was used for post‐hoc test. A two‐tailed Student's *t*‐test was used for comparisons between the two groups. All data were expressed as mean ± standard error of the mean (SEM). *P* < 0.05 was considered to indicate a statistically significant difference.

## 3 Results

### 3.1 High Hepatic SLC25A4 Expression is Associated with the Beneficial Effects of Exercise in MASLD

We performed transcriptomic analysis on liver biopsies from a well‐characterized MASH patient cohort enrolled in a clinical study at the University of Antwerp (Belgian registration number B30020071389). Briefly, patients were stratified into LSI responders and non‐responders based on histological outcomes assessed at the 1‐year follow‐up, and demographic, biochemical, and histological characteristics of both cohorts are summarized in Table S2. Using the training (GSE106737) and validation (GSE83452) cohorts, we identified that hub‐gene *SLC25A4* (encoding the ANT1 protein) was significantly upregulated in LSI responders relative to non‐responders (Figure 1A and Supplementary Results). The detailed step‐by‐step screening process and pipeline are provided in Figures S1–S3. Subsequently, we overexpressed *Slc25a4* in the livers of exercise‐subjected mice. Compared with exercise alone, thyroxine‐binding globulin (TBG) promoter‐driven *Slc25a4* overexpression significantly alleviated steatosis and inflammation (Figure 1B,C). *Slc25a4* overexpression shifted the hepatic transcriptome toward reduced *de novo* lipogenesis (DNL), enhanced β‐oxidation, and attenuated inflammation (Figure 1D). Specifically, elevated Cpt1α and Pparα protein levels, together with reduced Fasn levels, promoted fatty acid oxidation (FAO), whereas decreased *Il1β*, *Tnfα*, and *Il6* expression and increased *Il10* expression exerted anti‐inflammatory effects, collectively promoting an FAO‐active and anti‐inflammatory state. (Figure 1D,E). Consistently, in our clinical sample set, baseline hepatic *Slc25a4* levels positively correlated with exercise‐induced improvement in Controlled Attenuation Parameter‐assessed liver steatosis (R = 0.700, *P* = 0.036). Patients with higher baseline *Slc25a4* expression exhibited significantly greater reductions in hepatic fat content following the exercise intervention (Figure S4). To determine whether Slc25a4‐mediated reduction in steatosis and inflammation relies solely on exercise rather than dietary restriction, we overexpressed *Slc25a4* in the livers of mice under dietary restriction (Slc25a4 mice). No significant change in steatosis or inflammation was observed in the livers of Slc25a4 mice under dietary restriction (Figure S5A–C). Under dietary restriction, liver‐specific expression of *Slc25a4* failed to change the expression of genes controlling *de novo* lipogenesis (DNL), FAO, and hepatic inflammation (Figure S5D,E). We next investigated the effect of Slc25a4 on the MASLD induced by high‐fat, high‐fructose, and high‐cholesterol diet (HFFC) (Figure 1F–H). Hematoxylin and eosin (H&E) staining and morphological analysis revealed that *Slc25a4* overexpression failed to alter hepatic steatosis and inflammation after 8 or 16 weeks of HFFC treatment (Figure 1F,G). Consistently, no significant change was observed in the expression of genes controlling DNL, FAO, and inflammation in livers of *Slc25a4* ‐overexpressing mice (Figure 1I). Together, Slc25a4 mediates the metabolic benefits of exercise but not of caloric restriction.

**FIGURE 1:**
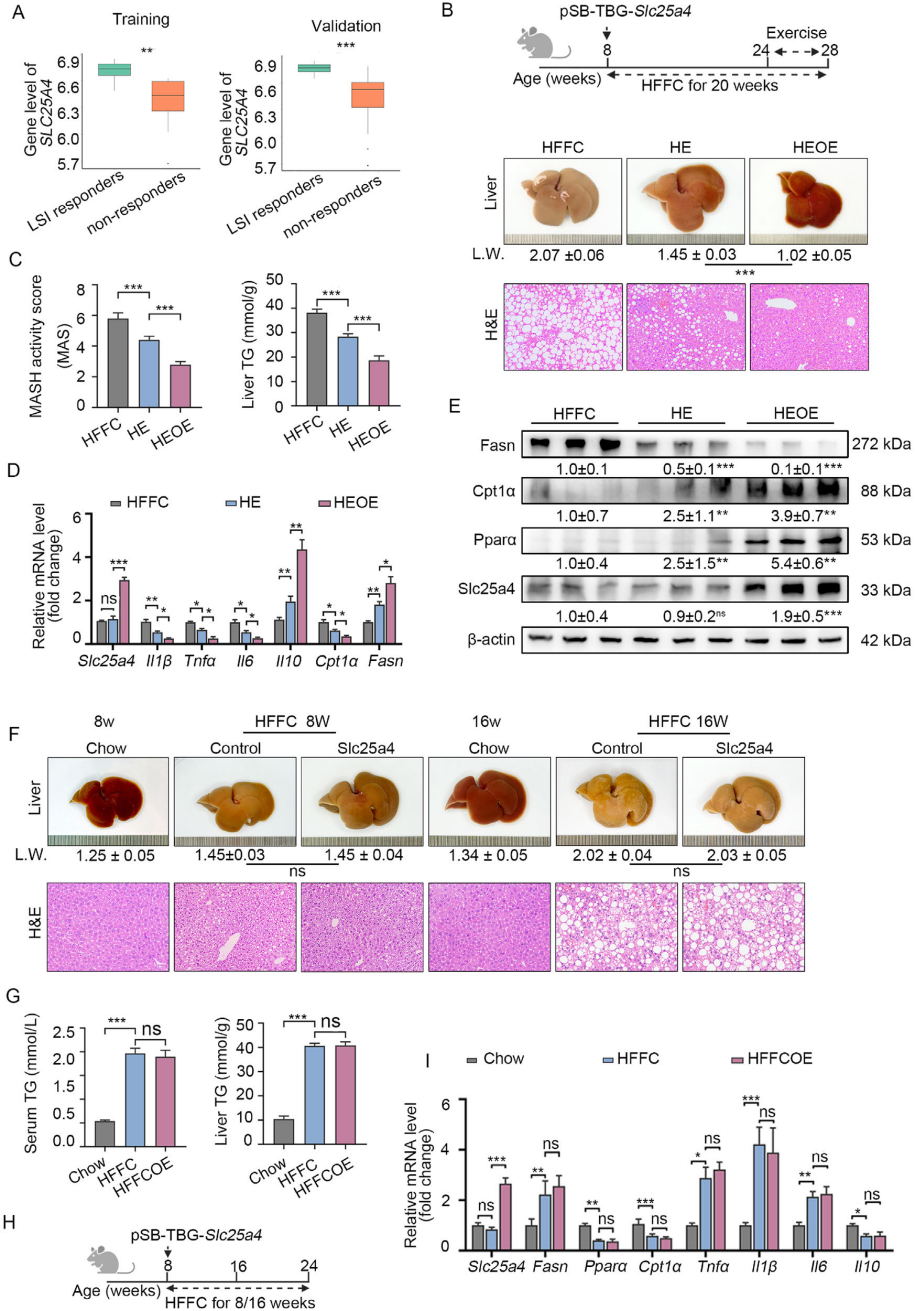
Hepatocyte‐specific expression of Slc25a4 significantly enhanced exercise‐mediated improvement of MASLD. (A) Expression levels of *Slc25a4* in LSI responders and non‐responders in the training and validation cohorts. LSI: lifestyle intervention. (B) Macroscopic and microscopic (H&E, original magnification, ×200) appearance of livers from HFFC mice (*n* = 6), HE (*n* = 6) and HEOE mice (*n* = 6). (C) MASH activity score (MAS) and liver Triglyceride (TG) content in the livers of three groups of mice. (D) mRNA levels of genes encoding Il1β, Tnfα, Il6, Il10, Cpt1α, Fasn and Slc25a4 in livers of three groups of mice. (E) Protein levels of Fasn, Cpt1α, Pparα and Slc25a4 in livers of three groups of mice. (F) Macroscopic and microscopic (H&E, original magnification, ×200) appearance of livers from mice maintained on the chow diet (*n* = 6) or HFFC (*n* = 6) or hepatocyte‐specific expression of *Slc25a4* mice maintained on the HFFC diet for 8 weeks and 16 weeks (*n* = 6). (G) TG content of livers and serum from chow mice, HFFC mice and HFFCOE mice at 16 weeks. (H) Schematic diagram of HFFC treatment of hepatocyte‐specific expression of *Slc25a4* mice. *Slc25a4* mice were fed either a HFFC diet or chow diet for 8 weeks or 16 weeks. (I) mRNA level of genes encoding Fasn, Cpt1α, Pparα, Il1β, Tnfα, Il6, Il10 and Ant1 in the livers from chow mice, HFFC mice and HFFCOE mice at 16 weeks. HFFC: high‐fat, high‐fructose, and high‐cholesterol diet; HE: HFFC and exercise; HEOE: HE mice with *Slc25a4* overexpression; HFFCOE: HFFC‐fed mice injected with *Slc25a4*; H&E: Hematoxylin and Eosin. L.W., liver weight. Data represent mean ± standard error of the mean. *P < 0.05, **P < 0.01, and ***P < 0.001; ns, not significant (A: two‐tailed Student's t‐test; C, D, E, G and I: one‐way ANOVA followed by Tukey's post hoc test). Schematic diagrams were created with BioRender.com. Original blots can be found in Supporting Information.

### 3.2 Macrophage Depletion Abolishes the Ability of Slc25a4 to Alleviate MASLD During Exercise

Considering the complex immune microenvironment in MASLD, we next employed single‐cell RNA sequencing (scRNA‐seq) to analyze the cellular landscape and immune profile in mice treated with HFFC, HFFC following exercise (HE) or HE mice with liver‐specific overexpression of *Slc25a4* (HEOE). Through unsupervised clustering and classical marker gene annotation, we identified nine main cell types in the liver including hepatocytes, cholangiocytes, T/NK cells, macrophages, dendritic cells (DCs), hepatic stellate cells (HSCs), endothelial cells, neutrophils, and B cells (Figure 2A). Significant changes in the macrophage population distribution were observed across three groups of mice (Figure 2A). Using canonical markers (*Ccr2* and *Cd163*), we classified the five macrophage clusters into a pro‐inflammatory phenotype (M1‐like; clusters 4 and 9) and an anti‐inflammatory phenotype (M2‐like; clusters 6, 13, and 14) (Figure S6). Upon *Slc25a4* overexpression, pro‐inflammatory macrophages (M1‐like) decreased by over 70%, while anti‐inflammatory/resident macrophages (M2‐like) increased by over 70% (Figure 2B). M1‐like macrophages exhibited elevated expression of genes encoding pro‐inflammatory cytokines/chemokines (*Tnfα*, *Cxcl1*, *Cxcl2*, *Il1β*) and infiltration‐related genes (*Ccr2*), whereas M2‐like macrophages displayed increased expression of anti‐inflammatory genes encoding *Il10*, *Cd163*, *Adipoq* and liver resident macrophage‐related genes (*Clec4f*) (Figure 2C).

**FIGURE 2:**
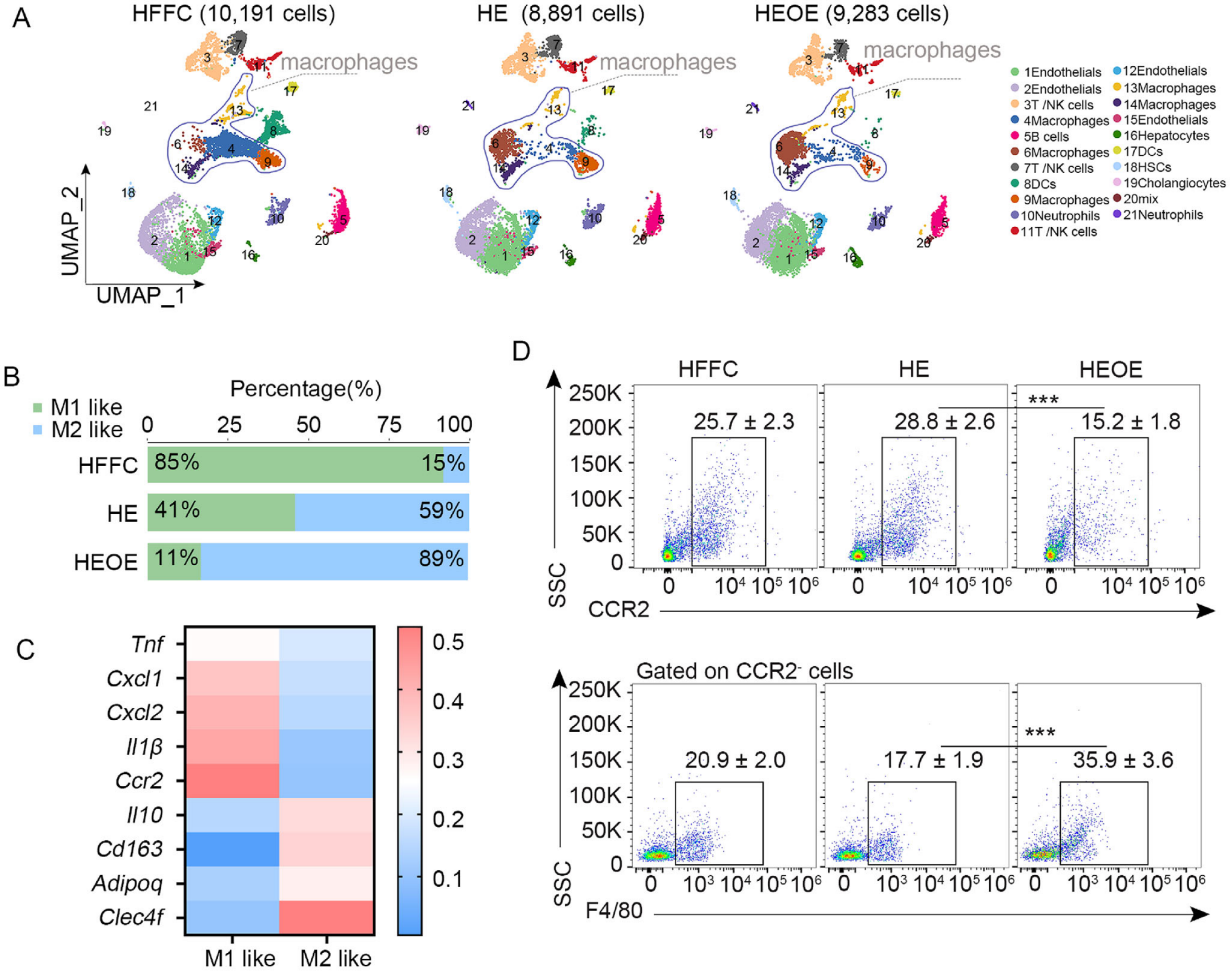
Hepatocyte‐specific Slc25a4 overexpression reshapes hepatic macrophage landscape during exercise. (A) scRNA analysis of livers from HFFC mice, HE mice, and HEOE mice. (B) Ratio of M1‐like cells or M2‐like cells to macrophages in livers of 3 groups of mice. (C) Levels of genes encoding M1‐like or M2‐like markers in liver of 3 groups of mice. (D) Ratios of F4/80^+^/CCR2^+^ in livers of three groups of mice, as revealed by flow cytometry. HFFC: high‐fat, high‐fructose, and high‐cholesterol diet; HE: HFFC and exercise; HEOE: HE mice with *Slc25a4* overexpression. Data represent mean ± standard error of the mean. *P < 0.05, **P < 0.01, and ***P < 0.001; ns, not significant (D: one‐way ANOVA followed by Tukey's post hoc test).

Flow cytometry confirmed that Clec4f^+^ macrophages (Kupffer cells, KC) are the major macrophages of the liver and are gradually exhausted in the development of MASLD (Figure S7). We speculate that overexpressed *Slc25a4* in hepatocytes enhances the response of MASLD to exercise by restoring the KC pool. Specifically, hepatocyte‐specific overexpression of *Slc25a4* restored exhausted Clec4f^+^ macrophages while decreasing Ccr2^+^ macrophages (Figure 3A,B). Flow cytometry confirmed that *Slc25a4* overexpression reduced hepatic infiltration of CCR2^+^ macrophages, while increasing F4/80^+^ macrophages in the livers of HEOE mice (Figure 2D). In the CCR2^−^ gate, a significant decrease in Clec4f^+^Tim4^+^ macrophage population was noted in HFFC mice, while a marked increase in Clec4f^+^Tim4^+^ macrophage levels was detected in HEOE mice (Figure 3C). Considering the roles of macrophages on steatosis and inflammation, we analyzed the polarization status of macrophages in HEOE mice. As expected, levels of M2‐like KCs (CD163^+^Clec4f^+^) were significantly increased in HEOE mice (Figure 3D,E).

**FIGURE 3:**
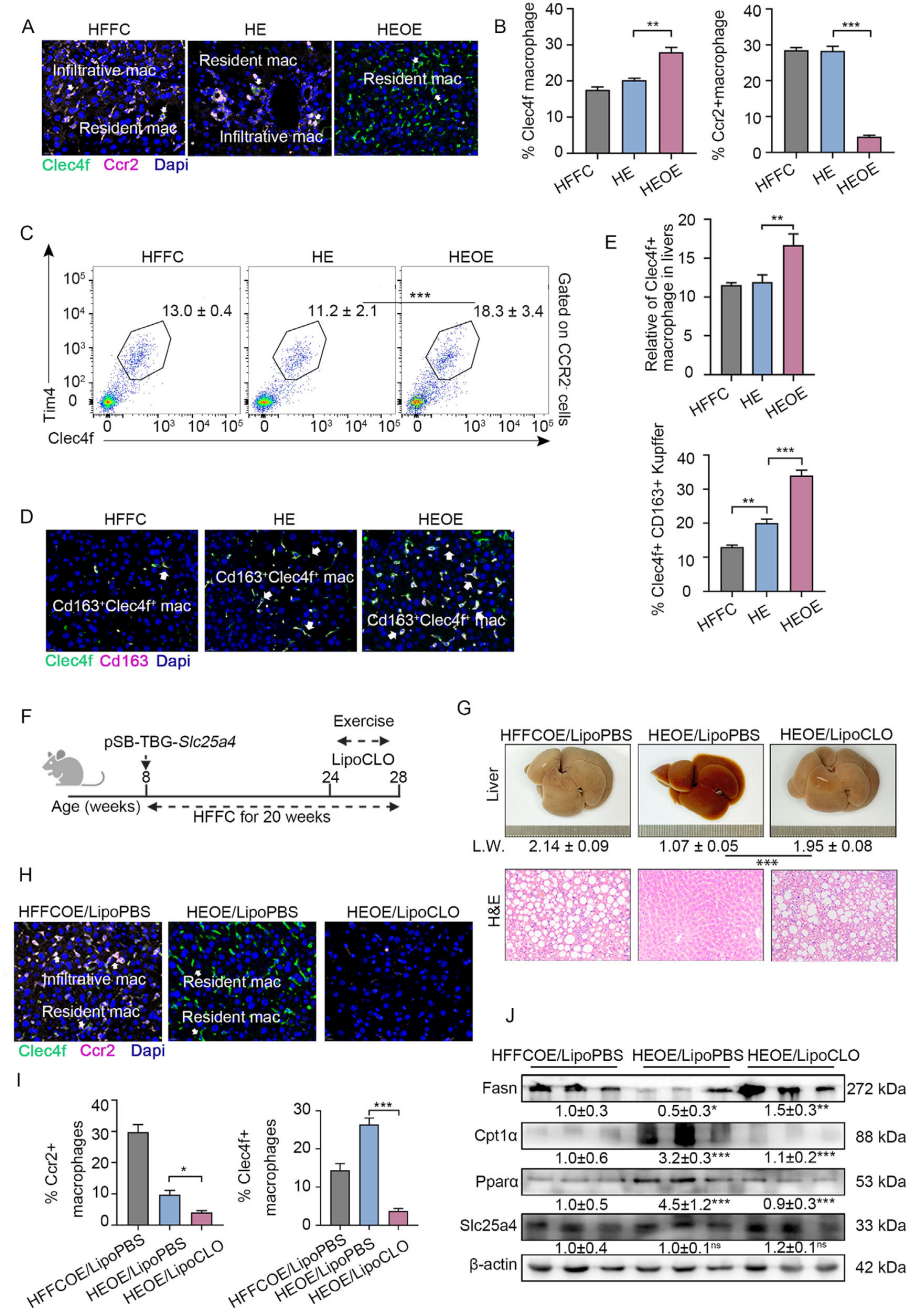
Hepatocyte‐specific expression of *Slc25a4* augmented macrophage recruitment in exercised mice. (A‐B) Immunostaining of Ccr2 and Clec4f markers in livers of three groups of mice. The arrow represents Ccr2 or Clec4f positive cells. (C) Ratios of Tim4^+^Clec4f^+^ in livers of three groups of mice, as revealed by flow cytometry. (D‐E) Immunostaining of Cd163 and Clec4f markers in livers of three groups of mice. The arrow represents Cd163^+^Clec4f^+^ cells. (F) Schematic of the experimental design: *Slc25a4*‐overexpressing mice on HFFC diet with 4‐week exercise training and/or clodronate liposome‐mediated macrophage depletion (LipoCLO). (G) Macroscopic and microscopic (H&E, original magnification, ×200) appearance of livers from HFFCOE/LipoPBS mice (*n* = 6), HEOE/LipoPBS mice (*n* = 6) and HEOE/LipoCLO mice (*n* = 6). (H‐I) Immunostaining of Ccr2 and Clec4f markers in livers of three groups of mice. The arrow represents Ccr2 or Clec4f positive cells. (J) Protein levels of Fasn, Cpt1α, Pparα and Slc25a4 in livers of three groups of mice. HFFC: high‐fat, high‐fructose, and high‐cholesterol diet; HE: HFFC and exercise; HEOE: HE mice with *Slc25a4* overexpression; HFFCOE: HFFC‐fed mice injected with *Slc25a4*. LipoPBS: Control Liposomes (PBS); LipoCLO: Clodronate Liposomes. L.W., liver weight. Data represent mean ± standard error of the mean. *P < 0.05, **P < 0.01, and ***P < 0.001; ns, not significant (B, C, E, I and J: one‐way ANOVA followed by Tukey's post hoc test). Schematic diagram was created with BioRender.com. Original blots can be found in Supporting Information.

To test whether macrophages mediate the role of Slc25a4 in driving the benefits of exercise, we depleted macrophages in the livers of HEOE mice (Figure 3F). Macrophage depletion counteracted the role of *Slc25a4* overexpression in alleviating hepatic steatosis and inflammation in HEOE mice (Figure 3G–I). Consistent with this observation, FAO‐related proteins including Cpt1α and Pparα were decreased, while the rate‐limiting enzyme of DNL, Fasn, was increased in the livers of HEOE mice with depleted macrophages (Figure 3J). Furthermore, we also treated the HFFC model with clodronate liposomes. Strikingly, clodronate liposomes attenuated hepatic steatosis and inflammation in HFFC mice, accompanied by corresponding downregulation of lipogenic and inflammatory genes—an effect most likely mediated by the depletion of M1‐like macrophages, as discussed below (Figure S8). Given that the single‐cell atlas reveals substantially divergent macrophage expression programs among HFFC, HE, and HEOE contexts, our results highlight the necessity of defining the functionally predominant macrophage phenotype that governs the outcome in each setting.

Together, hepatocyte‐specific overexpression of *Slc25a4* enhances the efficacy of exercise by restoring resident macrophage/KC pools.

### 3.3 Slc25a4 Alleviates Hepatic Steatosis and Inflammation by Facilitating the Replenishment of Infiltrative Macrophages that Differentiate into the Resident Macrophage Niche

Fatty acid stimulation promoted expression of genes encoding M1‐like markers including *Tnfα*, *Il6*, *Ptgs2*, and *Ccl5*, while reducing M2‐like marker genes encoding *Mgl2*, *Il10*, *Mgl1*, and *Arg1* (Figure S9), suggesting that lipid accumulation is potentially involved in macrophage‐mediated enhancement of exercise benefits. Given the distribution between liver‐resident macrophages and circulating macrophages, we speculated that differentiation of circulating macrophages into resident macrophages is potentially impaired by the high‐fat environment. Single‐cell analysis revealed four major macrophage subsets in the liver: *Ly6c^+^Ccr2^+^* infiltrating monocytes/macrophages, *Adgre1* ^+^Ly6c^−^ mature resident macrophages, *Clec4f^+^Timd4^−^* transitional macrophages, and a specialized *Clec4f^+^Timd4^+^* Kupffer cell population (Figure 4A). Notably, *Clec4f^+^Timd4^+^* macrophages in the HFFC group were exhausted, accompanied by the recruitment of circulating macrophages (*Ly6c^+^Ccr2^+^* macrophages) (Figure 4A). In HEOE mouse livers, *Clec4f^+^Timd4^+^* macrophages represented the predominant subset (85.3% of total macrophages). In contrast, HFFC mice exhibited a significant reduction in the *Clec4f^+^Timd4^+^* macrophage subset (to 8.8%), with *Ly6c^+^Ccr2^+^* macrophages representing the dominant population, comprising 41.6% of the total (Figure 4B). We speculate that *Slc25a4* overexpression enhances exercise responsiveness by facilitating the conversion of infiltrating macrophages into a resident, lipid‐clearing (*Clec4f^+^Timd4^+^*) phenotype. Trajectory inference demonstrated a monocyte‐to‐resident macrophage differentiation continuum, originating from *Ccr2^+^Ly6c^+^* cells and terminating in *Clec4f^+^Timd4^+^* KCs (Figure 4C). We next employed CCR2 monoclonal antibody (mAb) to block macrophage recruitment (Figure 4D). As anticipated, CCR2 neutralization led to a significant reduction in the Clec4f^+^ macrophage population in the livers of HEOE mice (Figure 4E), indicating that circulating macrophages are the major source of *Clec4f^+^Timd4^+^* KCs. Phenotypically, CCR2 neutralization exacerbated hepatic steatosis in the livers of HEOE mice (Figure 4F,G). Liver triglyceride content was significantly elevated in HEOE mice following CCR2 neutralization (Figure 4H). Enhanced DNL (increased Fasn) and impaired FAO (decreased Cpt1α) were observed in HEOE mice following CCR2 neutralization (Figure 4I,J). Together, Slc25a4 overexpression alleviates hepatic steatosis and inflammation by facilitating the replenishment of monocytes into the resident macrophage niche.

**FIGURE 4:**
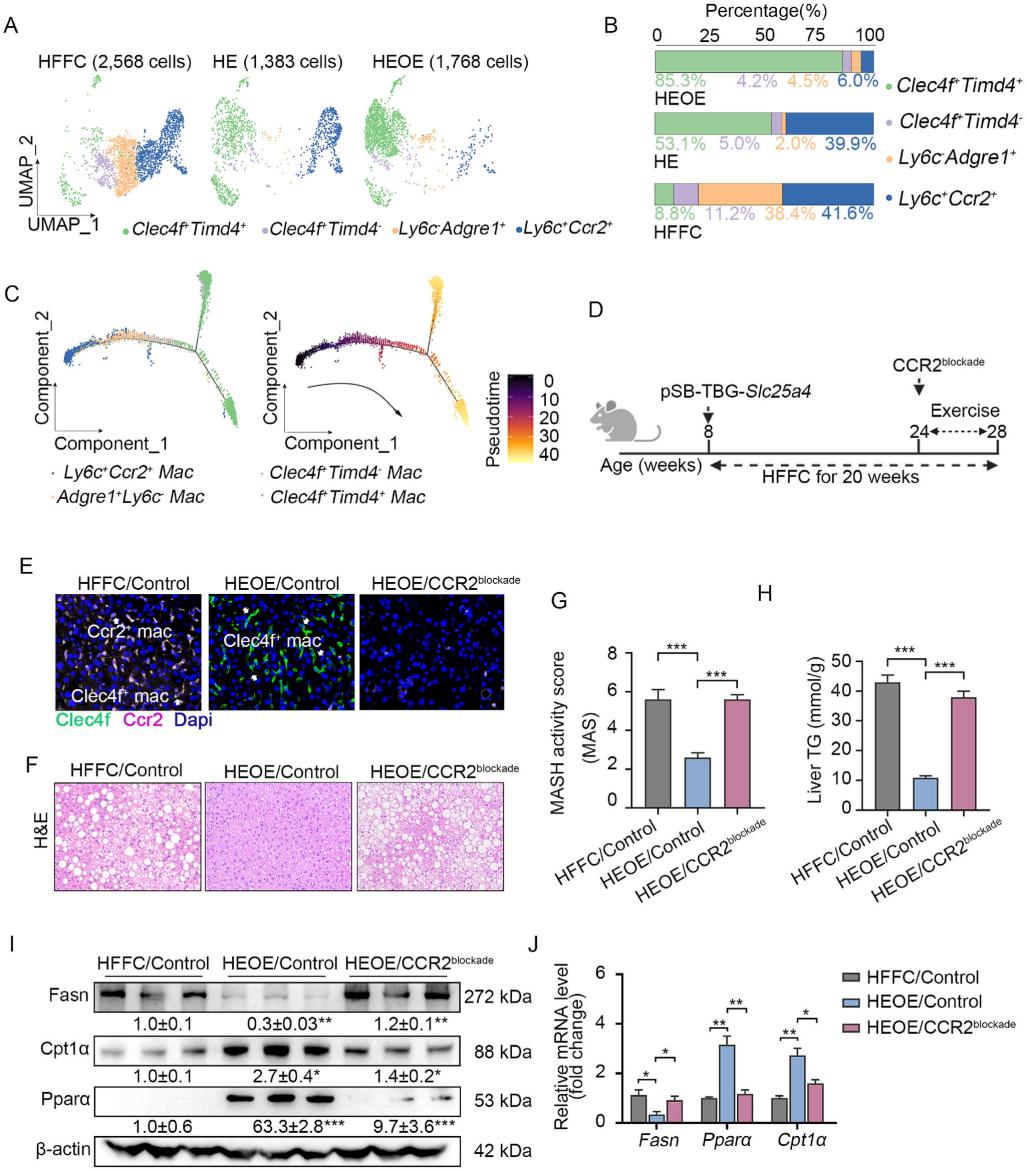
CCR2 blockade eliminates the beneficial effects of exercise in *Slc25a4*‐overexpressing mice. (A) scRNA‐seq identifies distinct macrophage subpopulations altered by diet‐induced steatosis and rescued by hepatocyte‐specific *Slc25a4* overexpression. (B) Proportions of four hepatic macrophage subsets in livers of three groups of mice. (C) Monocyte‐to‐macrophage differentiation trajectories identified by pseudotime analysis in livers of three groups of mice. (D) Schematic of the experimental design: *Slc25a4* mice on HFFC diet with 4‐week combined exercise training and CCR2 blockade. (E) Immunostaining of Ccr2 and Clec4f markers in livers of three groups of mice. The arrow represents Ccr2^+^ or Clec4f^+^ cells. (F) Microscopic (H&E, original magnification, ×200) appearance of livers from HFFC/Control (*n* = 6), HEOE/Control (*n* = 6) and HEOE/CCR2^blockade^ (*n* = 6). (G‐H) MASH activity score (MAS) and liver TG content in the livers of three groups of mice. (I) Protein levels of Fasn, Cpt1α and Pparα in livers of three groups of mice. (J) mRNA levels of genes encoding Fasn, Cpt1α and Pparα in livers of three groups of mice. HFFC: high‐fat, high‐fructose, and high‐cholesterol diet; HE: HFFC and exercise; HEOE: HE mice with *Slc25a4* overexpression. Data represent mean ± standard error of the mean. *P < 0.05, **P < 0.01, and ***P < 0.001; ns, not significant (G, H, I, and J: one‐way ANOVA followed by Tukey's post hoc test). Schematic diagram was created with BioRender.com. Original blots can be found in Supporting Information.

### 3.4 Clec4f^+^Tim4^+^P2ry12^+^ Eating Macrophages Prevented Aberrant Accumulation of Lipid‐Laden Hepatocytes in the Liver

We next investigated how *Clec4f^+^* macrophages are responsible for reduced hepatic steatosis and inflammation in HEOE mice. Functional enrichment analysis revealed distinct molecular signatures across macrophage subpopulations. *Clec4f^+^Timd4^+^* macrophages exhibited increased proliferation (E2F transcription factor, E2F targets) and tissue repair pathways (transforming growth factor‐β, TGFβ signaling). *Clec4f^+^Timd4^−^* macrophages showed enhanced expression of lipid metabolism‐related genes (adipogenesis and fatty acid metabolism). In contrast, *Ccr2^+^Ly6c^+^* and *Adgre1^+^Ly6c^−^* macrophages displayed increased activity in inflammatory (TNFα signaling via NFκB) and metabolic (Oxidative phosphorylation) pathways (Figure 5A and Figure S10). Among the four macrophage subpopulations, *Clec4f^+^Timd4^+^* macrophages, induced by Slc25a4 overexpression, represent the predominant subset in the livers of HEOE mice. Sub‐clustering of *Clec4f^+^Timd4^+^* macrophages via dimensionality reduction revealed a dominant cluster (cluster 1) that constitutes the majority of this KC population (Figure 5B). Cluster 1 of *Clec4f^+^Timd4^+^* macrophages exhibited high expression of *P2ry12*, *Cd163*, *Gas6*, and *Mertk*, consistent with enhanced find‐me signaling (*P2ry12*) and efferocytic capacity (*Gas6 and Mertk*) (Figure 5C,D). Cluster 1, characterized by high co‐expression of *Clec4f*, *Tim4*, and *P2ry12*, was designated as the lipid‐eating macrophage cluster.

**FIGURE 5:**
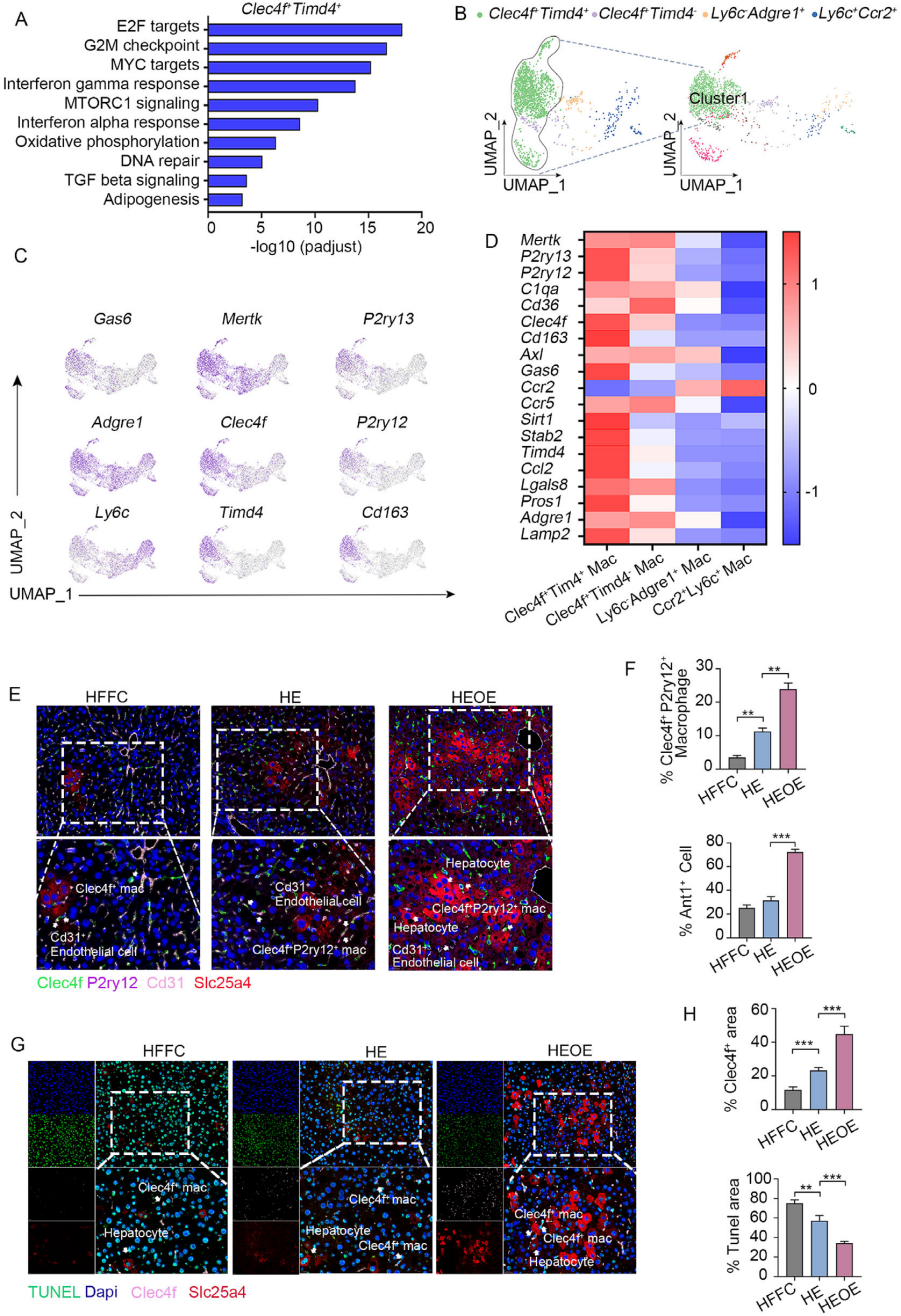
Recruitment of *Clec4f^+^Timd4^+^P2ry12^+^* eating macrophages promotes removal of lipid‐laden hepatocytes. (A) Enriched pathways in *Clec4f^+^Timd4^+^* macrophages. (B‐C) Re‐clustering of *Clec4f^+^Timd4^+^* macrophages with different characteristics. (D) Distinct gene profiles in four groups of *Clec4f^+^Timd4^+^*, *Clec4f^+^Timd4^−^*, *Adgre1^+^Ly6c^−^* and *Ccr2^+^Ly6c^+^* macrophages. (E‐F) Quantitative analysis of Clec4f^+^P2ry12^+^ eating macrophages and Slc25a4‐positive hepatocytes and Cd31^+^endothelial cells in three groups of mice, as revealed by immunostaining. The arrow represents Clec4f^+^P2ry12^+^ positive cells, Slc25a4‐positive hepatocytes and Cd31^+^endothelial cells. (G‐H) Immunostaining of TUNEL, Slc25a4 and Clec4f markers in livers of three groups of mice. HFFC: high‐fat, high‐fructose, and high‐cholesterol diet; HE: HFFC and exercise; HEOE: HE mice with *Slc25a4* overexpression. Data represent mean ± standard error of the mean. *P < 0.05, **P < 0.01, and ***P < 0.001; ns, not significant (F and H: one‐way ANOVA followed by Tukey's post hoc test).

Multiplex immunohistochemistry (mIHC) confirmed enriched Clec4f^+^P2ry12^+^ efferocytic macrophages in the livers of HEOE mice (Figure 5E,F). Co‐localization between *Slc25a4*‐overexpressing hepatocytes and recruited Clec4f^+^P2ry12^+^ efferocytic macrophages was observed in the livers of HEOE mice (Figure 5E). Terminal deoxynucleotidyl transferase dUTP nick end labeling (TUNEL) staining confirmed that lipid‐eating macrophages actively phagocytose apoptotic, lipid‐laden hepatocytes, thereby preventing their pathological accumulation in the liver (Figure 5G,H). In conclusion, *Slc25a4*‐overexpressing hepatocytes orchestrate the targeted recruitment of *Clec4f^+^P2ry12^+^* efferocytic macrophages to remove lipid‐laden dying hepatocytes.

### 3.5 Slc25a4 Triggers ATP Release via PANX1 Channels, Thereby Recruiting Lipid‐Eating Macrophages

We next investigated the mechanism by which *Slc25a4*‐overexpressing hepatocytes recruit lipid‐eating macrophages. As revealed by scRNA‐Seq, *P2ry12* is highly expressed in eating macrophages. P2RY12 is a purinergic receptor that is activated by extracellular nucleotides. Targeted metabolomics revealed that *Slc25a4*‐overexpressing primary hepatocytes released a high amount of ATP to extracellular space (Figure 6A). Overexpression of *Slc25a4* in hepatocytes robustly promoted ATP release, an effect reversed by *Slc25a4* knockdown (Figure 6B,C). Co‐culture and transwell migration assays showed that *Slc25a4*‐overexpressing hepatocytes, or their supernatant, enhanced macrophage migration, an effect abolished by *Slc25a4* knockdown or ATP depletion (Figure 6D). Furthermore, ATP depletion in the supernatant completely reduced the ability of the *Slc25a4*‐overexpressing hepatocytes to recruit lipid‐eating macrophages (Figure 6D). Supernatant from *Slc25a4*‐overexpressing hepatocytes upregulated anti‐inflammatory (*Nr4a1*, *Pbx1*), wound‐healing (*Areg*, *Ptgs2*), and efferocytosis‐related (*P2ry12*) genes in RAW264.7 cells (Figure 6E), indicating that lipid‐eating macrophages are M2‐like in nature. A major mechanism for ATP release from cells is via plasma membrane heptameric Pannexin 1 (PANX1) channels [27]. Overexpression of *SLC25A4* increased protein levels of PANX1 in liver cells, while its knockdown reduced them (Figure 6F). ATP assays revealed that silencing *PANX1* abolished the ability of Slc25a4 overexpression to increase extracellular ATP, indicating that Slc25a4 promotes ATP release via the PANX1 channel (Figure 6G).

**FIGURE 6:**
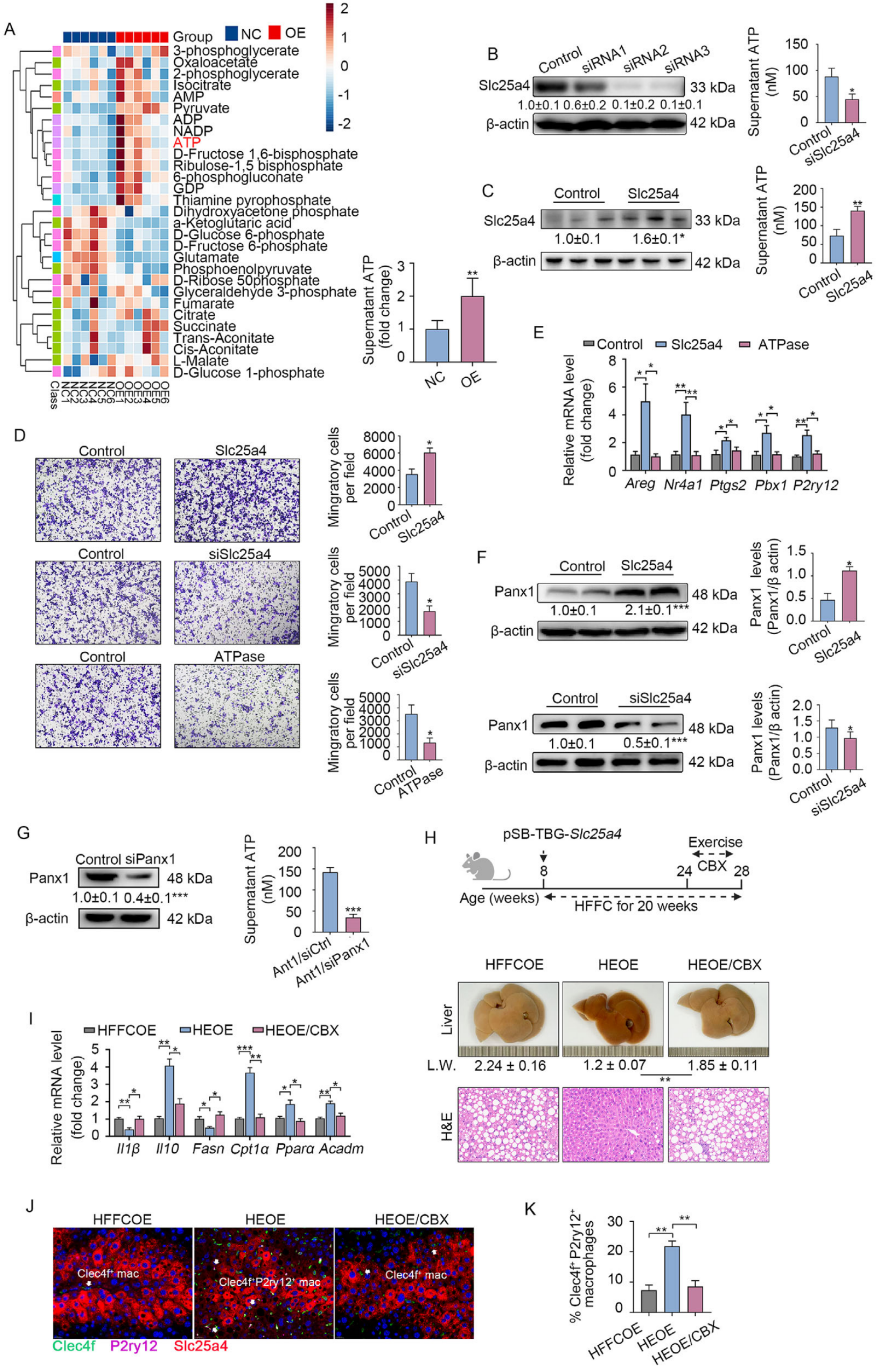
Slc25a4‐positive hepatocytes release high levels of ATP through PANX1 to recruit eating macrophages for phagocytosis. (A) Levels of metabolites including ATP in the culture supernatant of primary hepatocytes transduced with empty vector (NC) or *Slc25a4*‐overexpressing vector (OE), as revealed by targeted metabolomic analysis. (B) *Slc25a4* knockdown reduced ATP release. (C) Overexpression of *Slc25a4* increased ATP release. (D) Macrophage chemotaxis in response to changes in *Slc25a4* level or ATP concentrations. (E) mRNA levels of *Areg*, *Nr4a1*, *Ptgs2*, *Pbx1* and *P2ry12* respond to changes in *Slc25a4* or ATP. (F) Protein level of Panx1 under *Slc25a4* overexpression or si*Slc25a4*. (G) Knockdown of Panx1 blocked ATP release. (H) Macroscopic and microscopic (H&E, original magnification, ×200) appearance of livers from HFFCOE mice (*n* = 6), HEOE (*n* = 6) and HEOE/Carbenoxolone (CBX) mice (*n* = 6). (I) mRNA level of genes encoding Il1β, Il10, Fasn, Cpt1α, Pparα and Acadm in livers of three groups of mice. (J‐K) Immunostaining of P2ry12 and Clec4f and the ratios of Clec4f^+^P2ry12^+^ cells in livers of three groups of mice. The arrow represents Clec4f and P2ry12 positive macrophages. HEOE: HE mice with *Slc25a4* overexpression; HFFCOE: HFFC‐fed mice injected with *Slc25a4*. NC: negative control; OE: Slc25a4 overexpression. L.W., liver weight. Data represent mean ± standard error of the mean. *P < 0.05, **P < 0.01, and ***P < 0.001; ns, not significant (B, C, D, F and G: two‐tailed Student's *t*‐test; E, I and K: one‐way ANOVA followed by Tukey's post hoc test). Schematic diagram was created with BioRender.com. Original blots can be found in Supporting Information.

We next employed carbenoxolone (CBX) to block hepatic Panx1 channels, thereby inhibiting ATP release in mice (Figure 6H). Panx1 inhibition diminished *Slc25a4* overexpression‐mediated improvements in exercise and led to recurrence of MASLD (Figure 6H). Mechanistically, blocking Panx1 increased the expression of lipogenic genes (*Fasn*) and inflammatory cytokines/chemokines (*Il1β*), while reducing the expression of FAO‐related genes (*Acadm*, *Pparα* and *Cpt1α*) (Figure 6I). Similarly, inhibition of Panx1 impaired the ability of *Slc25a4*‐overexpressing hepatocytes to recruit Clec4f^+^P2ry12^+^ eating macrophages (Figure 6J,K). In conclusion, Slc25a4 overexpression in hepatocytes triggers ATP release via PANX1 channels, thereby recruiting macrophages to the liver.

### 3.6 GDF15 Signaling Between Eating Macrophages and Slc25a4‐Positive Hepatocytes is Required for the Alleviation of Hepatic Steatosis

We next investigated how Clec4f^+^P2ry12^+^ macrophages eat lipid‐laden hepatocytes and thereby improve hepatosteatosis. Based on single‐cell transcriptomic data, we systematically mapped the hepatic intercellular communication network across HFFC, HE, and HEOE groups. Figure 7A illustrates a progressive intensification of interactions between hepatocytes and non‐parenchymal cells following interventions, reaching maximum density in HEOE mice. While hepatocytes broadly engaged with various stromal and immune populations, the most prominent amplification of outgoing signals was specifically directed toward Clec4f^+^Tim4^+^ (lipid‐eating) macrophages (Figure 7A). Subsequent ligand–receptor analysis pinpointed that hepatocyte Slc25a4 overexpression primarily augmented the GDF15 signaling axis targeting this specific macrophage subset. Co‐culture with *Gdf15*‐overexpressing RAW264.7 macrophages markedly decreased lipid content in AML12 cells (Figure S11A). Consistent with previous observations in the mouse cell line, stimulation with recombinant GDF15 increased the protein levels of CPT1α and PPARα while decreasing those of FASN in the human cell line (THLE‐2 cells) (Figure S11B). These in vitro data indicated that GDF15 signaling in macrophages is capable of significantly suppressing lipid accumulation in hepatocytes. We next disrupted the communication between lipid‐eating macrophages and hepatocytes in the livers of HEOE mice (Figure 7B). Knocking down *Gdf15* in lipid‐eating macrophages attenuated the beneficial effects of *Slc25a4* overexpression on improving hepatic lipid accumulation (Figure 7C). As expected, hepatic lipid content was significantly increased following ablation of *Gdf15* in HEOE mice (Figure S12A). Consistent with this phenotype, knocking down *Gdf15* significantly reduced expression of FAO‐associated proteins (Cpt1α and Pparα), while up‐regulating expression of lipogenic Fasn (Figure 7D).

**FIGURE 7:**
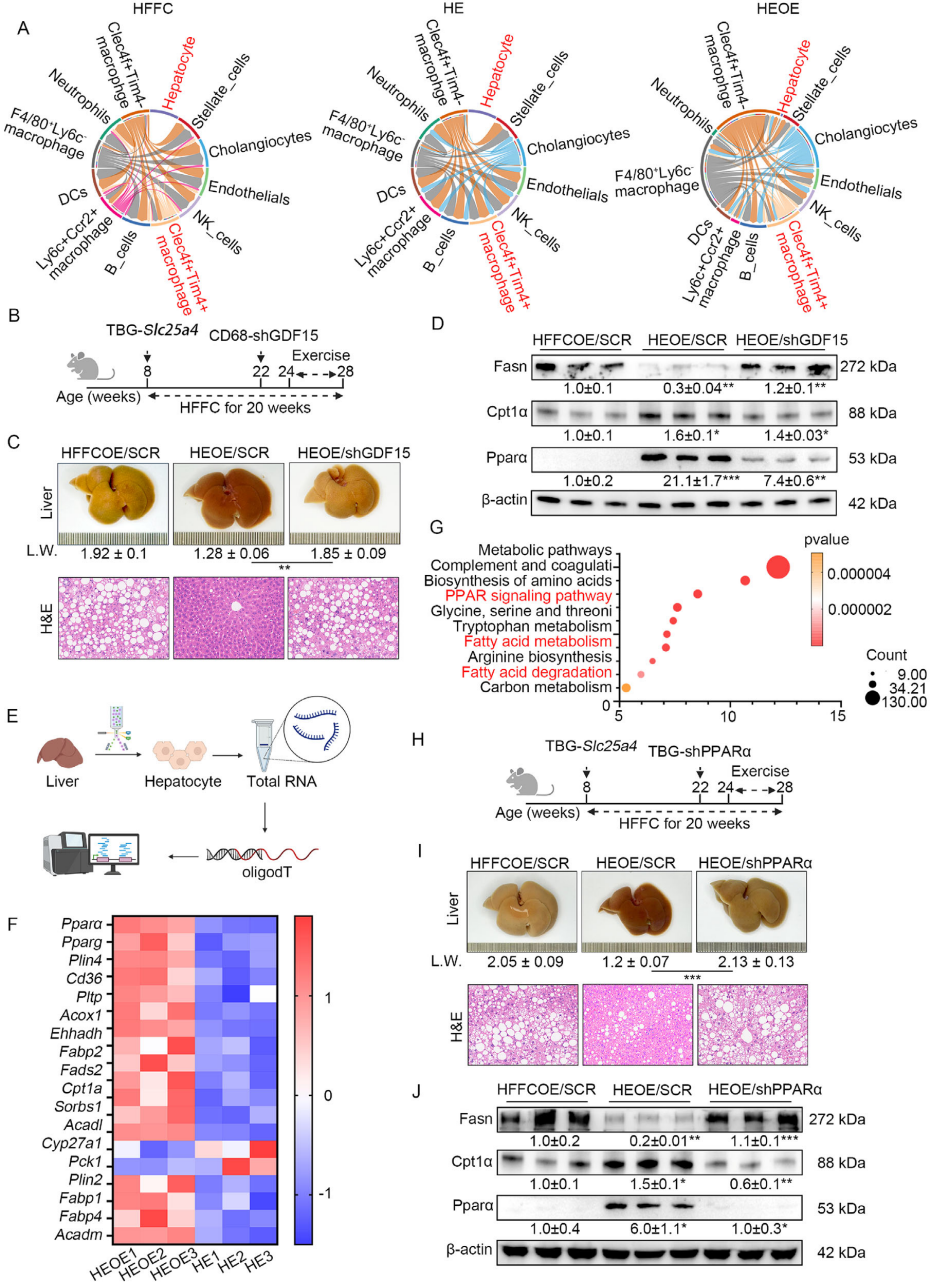
GDF15 mediated communication between eating macrophages and hepatocytes improves MASLD by activating PPARα signaling in hepatocytes. (A) Intercellular GDF signaling network visualized by chord diagram across different types of cells in the livers of three groups of HFFC, HE and HEOE mice. (B) Schematic of experimental design: hepatocyte‐specific *Slc25a4*‐overexpressing mice underwent 16 weeks of HFFC diet followed by 4 weeks of treadmill exercise with or without macrophage‐specific *Gdf15* knockdown. (C) Macroscopic and microscopic (H&E, original magnification, ×200) appearance of livers from HFFCOE/SCR mice (*n* = 6), HEOE/SCR mice (*n* = 6) and HEOE/CD68‐shGDF15 mice (n = 6). (D) Protein levels of Fasn, Cpt1α, and Pparα in livers of 3 groups of mice. (E) Workflow of hepatocyte isolation and RNA sequencing. (F) Heatmaps representing the upregulated and downregulated genes. (G) Enrichment analysis of differentially expressed genes. (H) Schematic diagram: *Slc25a4* mice were fed a HFFC diet for 16 weeks, followed by a 4‐week intervention with exercise and TBG‐shPPARα. (I) Macroscopic and microscopic (H&E, original magnification, ×200) appearance of livers from HFFCOE/SCR mice (n = 6), HEOE/SCR mice (*n* = 6) and HEOE/shPPARα mice (*n* = 6). (J) Protein levels of Fasn, Cpt1α and Pparα in livers of 3 groups of mice. HFFCOE: HFFC‐fed mice injected with *Slc25a4*. HEOE: HE mice with *Slc25a4* overexpression. H&E: Hematoxylin and Eosin. SCR: scramble. L.W., liver weight. Data represent mean ± standard error of the mean. *P < 0.05, **P < 0.01, and ***P < 0.001; ns, not significant (D and J: one‐way ANOVA followed by Tukey's post hoc test). Schematic diagrams were created with BioRender.com. Original blots can be found in Supporting Information.

We further evaluated the effect of GDF15 signaling activation in macrophages on the transcriptome in Slc25a4^high^ hepatocytes. *Slc25a4*‐overexpressing hepatocytes were isolated from the livers of HEOE mice via flow cytometry (Figure 7E). RNA‐Seq analysis revealed a significant increase in fatty acid degradation and fatty acid transport in Slc25a4^high^ ‐mCherry^+^ cells (Figure 7F). Specifically, activation of PPAR signaling, fatty acid catabolism and fatty acid degradation were observed in Slc25a4^high^‐mCherry^+^ cells (Figure 7G). We speculate that lipid‐eating macrophages, through GDF15 ligand, interact with hepatocytes, thereby activating the PPAR signaling and attenuating the lipid accumulation in hepatocytes. To test this speculation, we knocked down *Pparα* in the livers of HEOE mice (Figure 7H). Indeed, *Pparα* knockdown impaired the ability of *Slc25a4* overexpression to drive FAO and reduce hepatic lipid accumulation (Figure 7I, Figure S12B). Decreased Cpt1α and Pparα and increased Fasn were observed in HEOE/shPPARα mice (Figure 7J). Together, lipid‐eating macrophages, by releasing GDF15, activate PPARα signaling and FAO in hepatocytes, reducing hepatic lipid accumulation.

## 4 Discussion

Lifestyle intervention is the first‐line treatment for MASLD [28]. A weight loss of 5% or more can significantly improve MASLD. However, some patients achieving significant weight loss (>5%) show no histological improvement [7]. These patients were defined as LSI non‐responders versus LSI responders. In this study, we observed that LSI responders accumulated more lipid‐eating macrophages that eliminate lipid‐laden hepatocytes, thereby alleviating hepatic steatosis.

The quest for the molecular trigger of this recruitment led us to the unexpected discovery of SLC25A4 in the liver. Although SLC25A4 is conventionally regarded as a muscle‐specific protein with low hepatic presence, our study reveals a fascinating ectopic gain‐of‐function scenario: its elevation in hepatocytes creates a specialized signaling conduit (SLC25A4‐PANX1‐ATP) that the liver inherently lacks. Critically, we found that other hepatic isoforms, such as SLC25A5, cannot compensate for this role, establishing SLC25A4 as an indispensable molecular switch for exercise sensitivity. While SLC25A4 is known to facilitate mitochondrial ADP/ATP transport and mediate uncoupling in skeletal muscle, its role in hepatic metabolic remodeling remains obscure [29, 30, 31, 32, 33]. Our data demonstrate that hepatic Slc25a4 overexpression synergizes with exercise to ameliorate MASLD. By bridging mitochondrial energy status with extracellular signaling, SLC25A4 acts as a potent sensitizer that translates physical activity into macrophage‐mediated disease regression, providing a mechanistic explanation for exercise resistance in non‐responding patients.

Exercise triggers hepatic accumulation of lipid‐eating macrophages that drive removal of lipid‐laden hepatocytes. Slc25a4^high^ hepatocytes recruit P2ry12^+^ lipid‐eating macrophages. P2RY12 is a purinergic receptor [34]. To explain this observation, we performed metabolomic analysis on the supernatant of Slc25a4^high^ hepatocytes. ATP, which is closely associated with the function of Slc25a4, significantly increased in the supernatant of Slc25a4^high^ hepatocytes. Macrophages recognize extracellular ATP released from damaged or dying cells through purinergic receptors such as P2X7 and P2Y2 [35]. Upon binding to ATP, macrophages are activated to engulf and clear cellular debris, thereby limiting inflammation, restraining aberrant cell proliferation, and promoting tissue repair [35]. PANX1 channels are essential for extracellular ATP release, and exercise promotes PANX1 channel opening, thereby enhancing ATP efflux [36]. As expected, blocking PANX1 impaired the release of ATP, which subsequently abrogated the capacity of *Slc25a4* overexpression to enhance exercise benefits. Thus, ATP release is not stochastic; *Slc25a4*, when overexpressed, purposefully channels ATP through PANX1 to execute its physiological functions. These ATP molecules act as find‐me signals, recruiting eating macrophages and enhancing their phagocytosis and anti‐inflammatory functions, thus accelerating the clearance of lipid‐laden and inflammation‐damaged hepatocytes.

Lipid‐eating macrophages are Clec4f‐positive, suggesting their potential origin from KCs. It has been reported that KCs progressively become exhausted as MASLD advances [37, 38, 39, 40]. Depletion of KCs is strongly associated with hepatic insulin resistance, indicating their important role in MASLD [41]. Macrophages recruited by *Slc25a4* overexpression are characterized by expression of *Clec4f* and *Tim4*, classic markers of KCs. We speculate that Slc25a4 overexpression enhances the renewal capacity of KCs or facilitates the transition between circulating macrophages and KCs, particularly in a MASLD context. CCR2 blockade confirmed that circulating macrophages also play a critical role in *Slc25a4* overexpression‐mediated responses to exercise. Phenotypically, in our initial single‐cell transcriptomic evaluation, hepatic macrophages were broadly categorized into M1‐like (pro‐inflammatory) and M2‐like (anti‐inflammatory/tissue‐remodeling) populations. It should be clarified that this operational M1/M2 stratification served solely as a preliminary functional screening to capture the overall immunological shift toward an anti‐inflammatory tone following MASLD improvement. M1 macrophages secrete pro‐inflammatory cytokines, driving local inflammation and hepatic steatosis, whereas M2‐polarized macrophages play an essential protective role in ameliorating hepatic steatosis and insulin resistance [42]. Consequently, macrophage depletion at the different disease stage may yield divergent outcomes depending on the predominant macrophage phenotype. Recognizing that this classical dichotomy simplifies the intricate macrophage spectrum, we further performed fine‐grained subclustering analysis to dissect their true cellular heterogeneity. Through this deep characterization, we confirmed that these lipid‐eating macrophages—which broadly fall into the M2‐like functional spectrum—exhibited prominent expression of the classic M2 marker CD163 as well as Mertk, Gas6, and Lgals8, hallmarks of efferocytosis. Efferocytosis is the process where phagocytes (like macrophages) engulf apoptotic cells, curbing harmful content release and sustaining tissue balance. In MASLD, enhanced efferocytosis helps clear lipid‐laden and apoptotic hepatocytes. In sum, this group of lipid‐eating macrophages is responsible for alleviating MASLD by clearing lipid‐laden hepatocytes.

GDF15 signaling is required for eating macrophages to remove lipid‐laden hepatocytes. GDF15 is a stress‐induced cytokine belonging to the transforming growth factor‐beta (TGF‐β) superfamily, which is primarily involved in regulating cellular responses to stress, inflammation, and metabolic homeostasis [43]. Acute exercise increased muscle gene expression and serum GDF15 levels in both diabetic and non‐diabetic populations [44]. Moreover, liver‐derived GDF15 improves obesity and hepatic steatosis by decreasing appetite and increasing FGF21 [45]. Our results indicate that elevated GDF15 secretion enhances the FAO response in hepatocytes, facilitating their clearance by eating macrophages. However, while our findings highlight the critical role of hepatic macrophage‐derived GDF15, its precise upstream regulatory mechanisms remain to be fully elucidated. Established literature indicates that macrophage GDF15 production is tightly governed by cellular stress and transport networks, such as TFEB‐mediated transcription during lysosomal stress and ARRB1‐regulated post‐translational maturation [46, 47]. To explore specific transcriptional drivers in our model, we integrated Gdf15 promoter motif prediction with transcription factor profiling in lipid‐eating macrophages, pinpointing four high‐priority candidates: ATF4, STAT1, KLF6, and DLC1. Although experimental validation of these specific targets is beyond the scope of the current study, this analysis establishes a highly targeted roadmap for our independent, follow‐up investigations into these complex upstream regulatory networks.

Peroxisome proliferator‐activated receptor (PPAR) is a ligand‐activated transcription factor with three subtypes: α, β/δ, and γ [48]. PPARα, highly expressed in the liver, plays a key role in exercise‐induced adaptations by enhancing fatty acid oxidation [49, 50]. The enrichment of fatty acid degradation and the PPAR signaling pathway, together with the differential expression of PPARα, prompted us to focus on PPARα, the core regulator of fatty acid metabolism. Rescue experiments further confirmed this finding. Interestingly, however, PPARγ was also significantly upregulated in the HEOE group. Unlike PPARα, PPARγ is a more potent regulator of macrophage M2 polarization, anti‐inflammatory responses, and insulin sensitization, acting through NF‐κB suppression and adiponectin upregulation [51, 52]. This concurrent upregulation of PPARγ likely represents a compensatory mechanism that acts in concert with PPARα, attenuating the lipotoxicity associated with enhanced fatty acid oxidation during MASLD progression. This protective crosstalk highlights the functional synergy of dual PPAR targeting, a strategy strongly validated by clinical evidence where combined activation (e.g., dual‐ or pan‐PPAR agonists like saroglitazar and lanifibranor) demonstrates superior therapeutic efficacy in MAFLD/MASH over single‐subtype agonists [53, 54]. Future studies employing PPARγ‐specific knockdown or use of selective PPARγ antagonists are warranted to dissect its individual contribution.

To contextualize our findings, several limitations should be noted. The 16‐20‐week diet‐induced MASH model was selected to capture peak metabolic dysregulation and active inflammation. As robust liver fibrosis typically requires extended dietary regimens (>24 weeks) or secondary insults (e.g., CCl4), evaluating advanced fibrosis independently was beyond the scope of this cohort. Nonetheless, by reversing the core metabolic and inflammatory drivers during this window, SLC25A4‐mediated signaling effectively halts the triggers of downstream fibrotic progression. Future studies employing extended dietary durations are planned to directly investigate the long‐term impact of SLC25A4 on advanced liver fibrosis.

## Author Contributions

Y.Q., L.L., and Y.Z.: acquisition of data, analysis and interpretation of data, and drafting the manuscript. Y.M., Z.S., B.H., and G.S.: data analysis and manuscript editing. W.Y. and B.S.: obtaining funding, study supervision, study concept, design, and revision and final approval of the manuscript.

## Funding

The study is funded by the National Natural Science Foundation of China (Grant Number 82370579).

## Ethics Approval

All clinical procedures were approved by the Ethics Committee of Tongji Hospital of Tongji University (Approval No. 2023‐001). All animal experiments were approved by Animal Ethical and Welfare Committee of Tongji Hospital of Tongji University (Ethics No.20230101‐20240630‐DW‐004).

## Patient Consent for Publication

Consent obtained directly from patient(s).

## Conflicts of Interest

The authors declare no conflicts of interest.

## Supporting information




**Supporting File 1**: advs78004‐sup‐0001‐SuppMat.docx.

**Supporting File 2**: advs78004‐sup‐0002‐SuppMat.pdf.

## Data Availability

The data that supports the findings of this study are available in the Supporting Information of this article.
