# Supplementary material for: SLC25A4high Hepatocytes Enhance Lifestyle Intervention Response in MASLD by Recruiting Eating Macrophages
Source: Adv Sci (Weinh). 2026 Oct 15:e78004. Online ahead of print. doi: 10.1002/advs.78004 (PMC13672689; doi:10.1002/advs.78004)
Supplement: Supplementary file 1 [file ADVS-9999-e78004-s001.pdf]

SLC25A4^{high} Hepatocytes Enhance Lifestyle Intervention Response in MASLD by Recruiting Eating Macrophages

Original Western Blots

Qian et al

Figure 1E

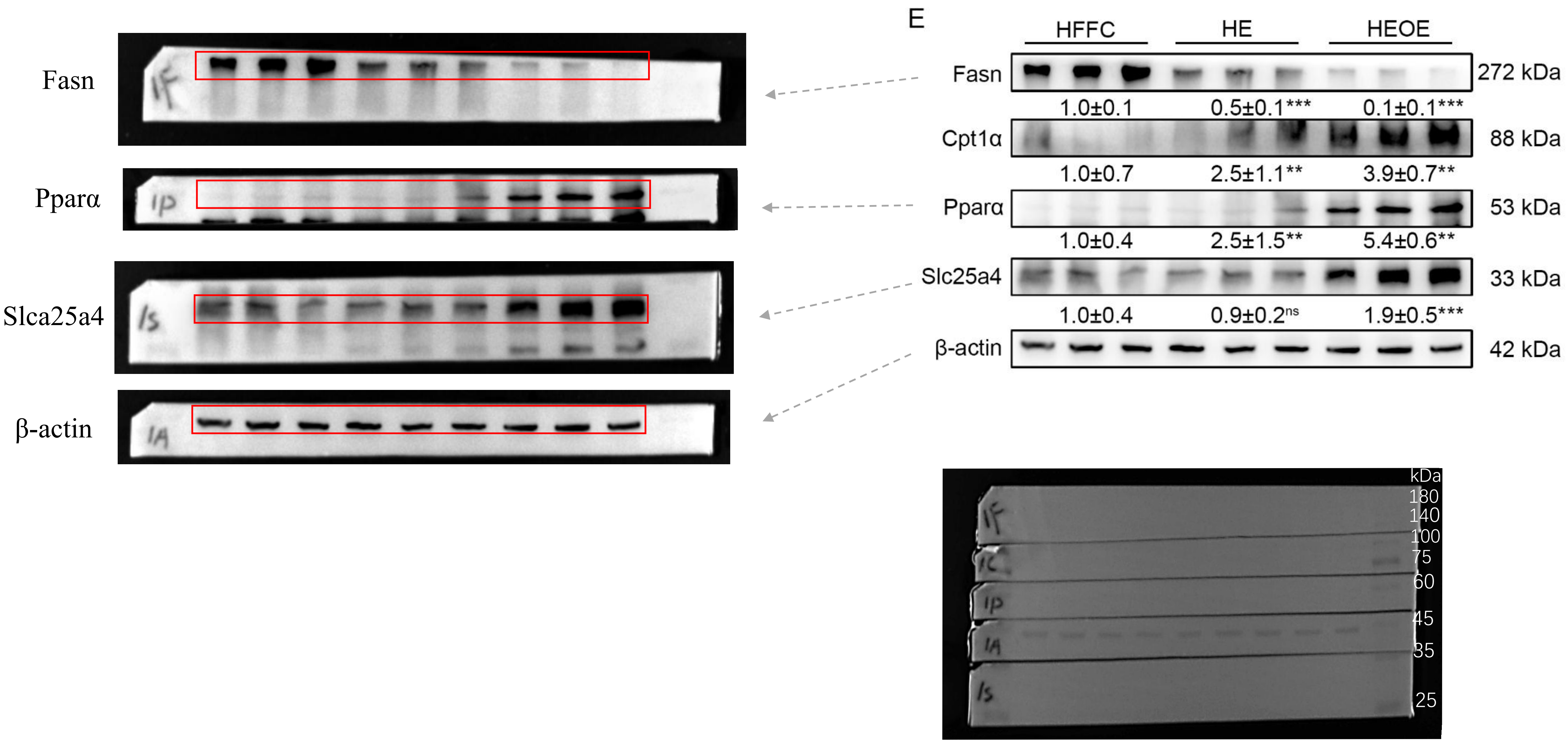
Fasn
Pparα
Slca25a4
β-actin
E
HFFC
HE
HEOE
Fasn
272 kDa
1.0±0.1
0.5±0.1***
0.1±0.1***
Cpt1α
88 kDa
1.0±0.7
2.5±1.1**
3.9±0.7**
Pparα
53 kDa
1.0±0.4
2.5±1.5**
5.4±0.6**
Slc25a4
33 kDa
1.0±0.4
0.9±0.2ns
1.9±0.5***
β-actin
42 kDa
kDa
180
140
100
75
60
45
35
25

Figure 1E

Cpt1α

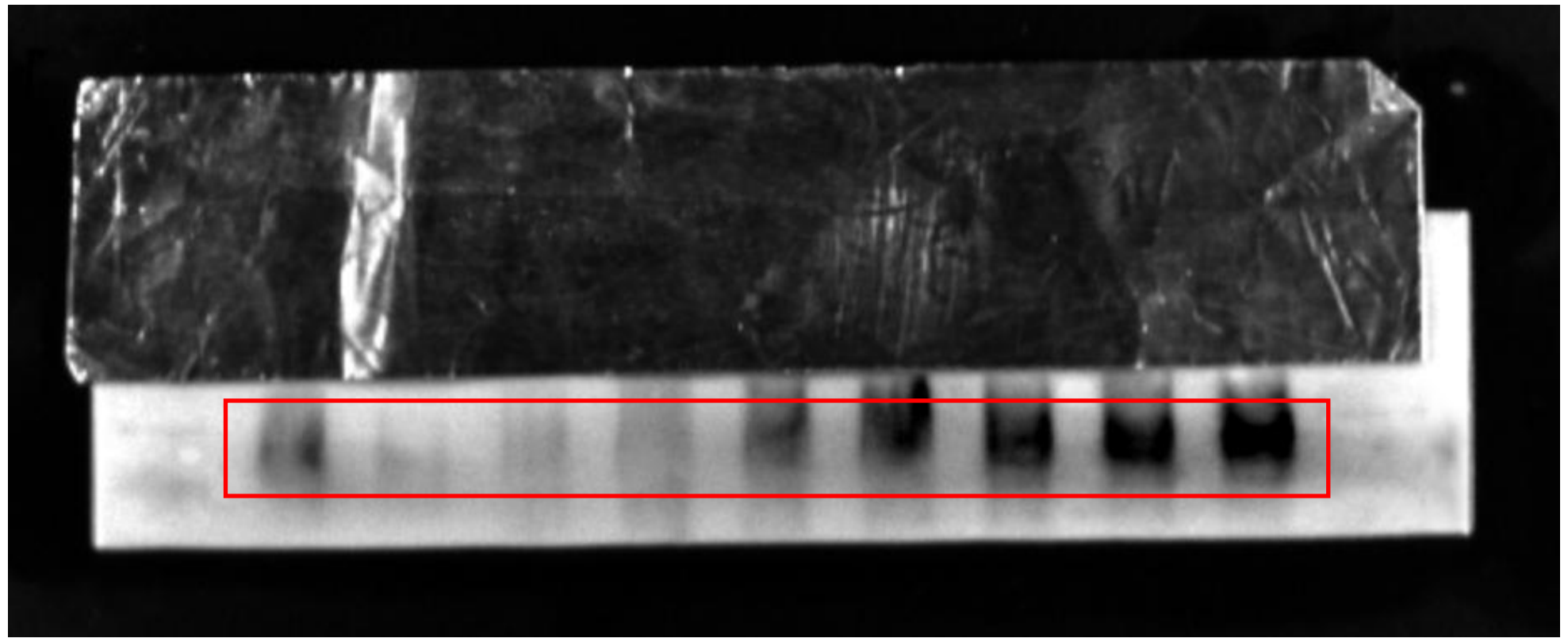

β-actin

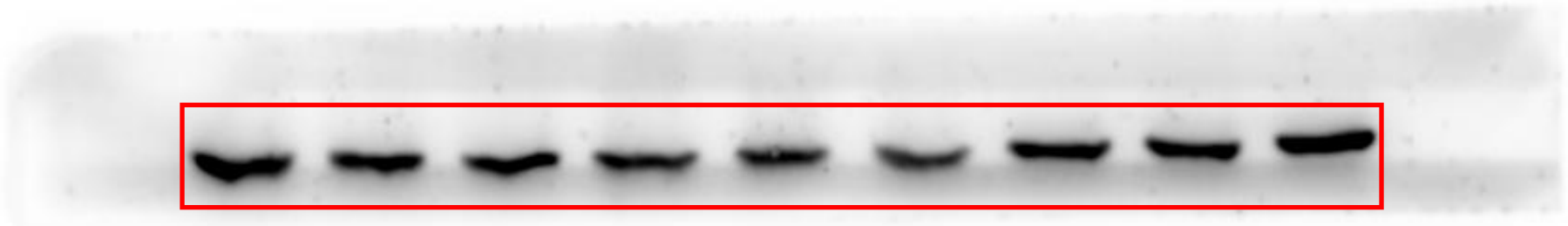

Slca25a4

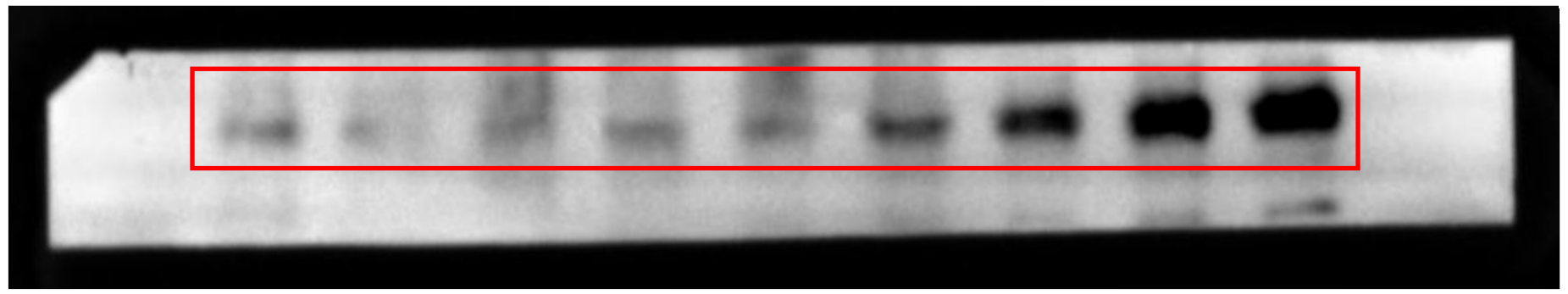

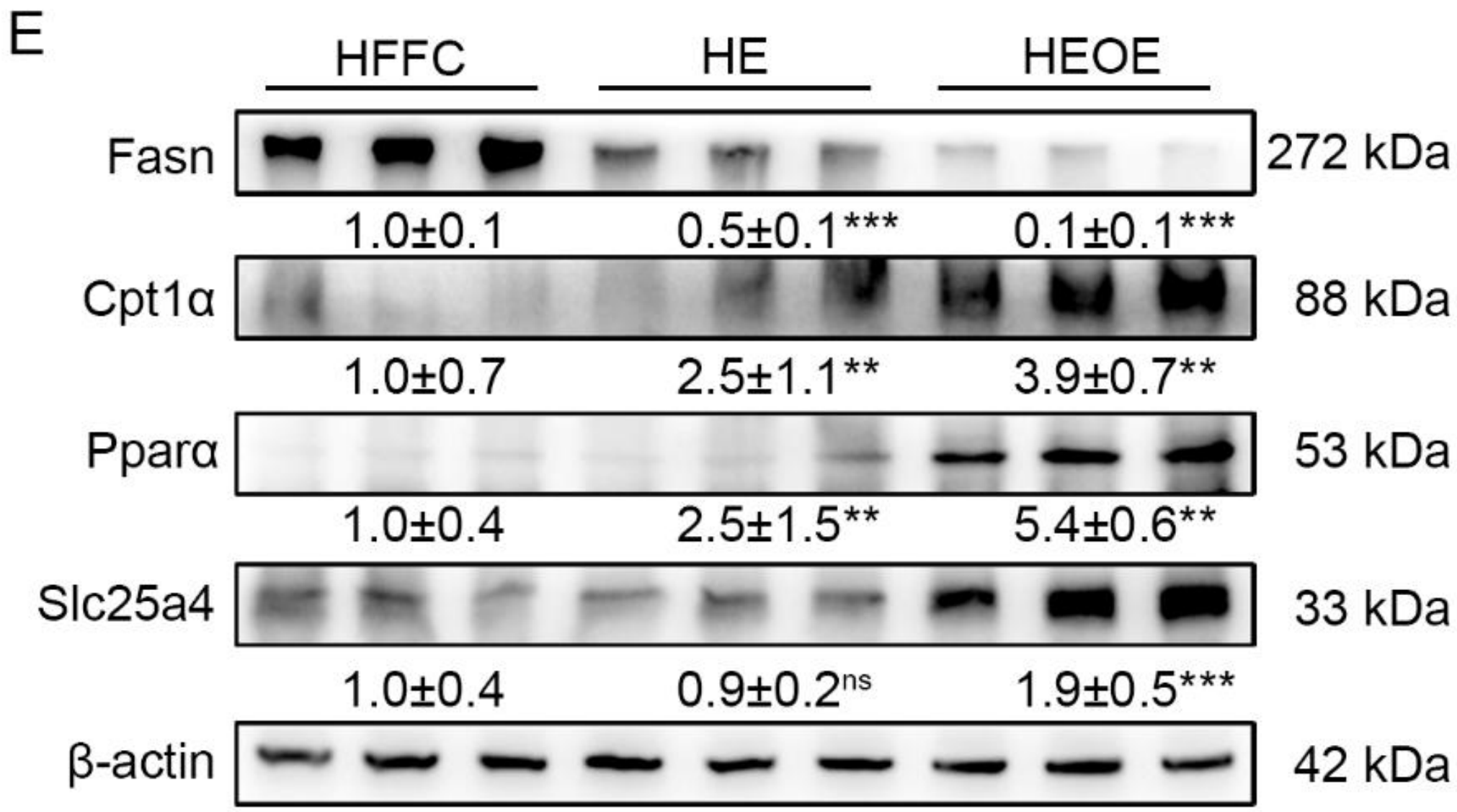

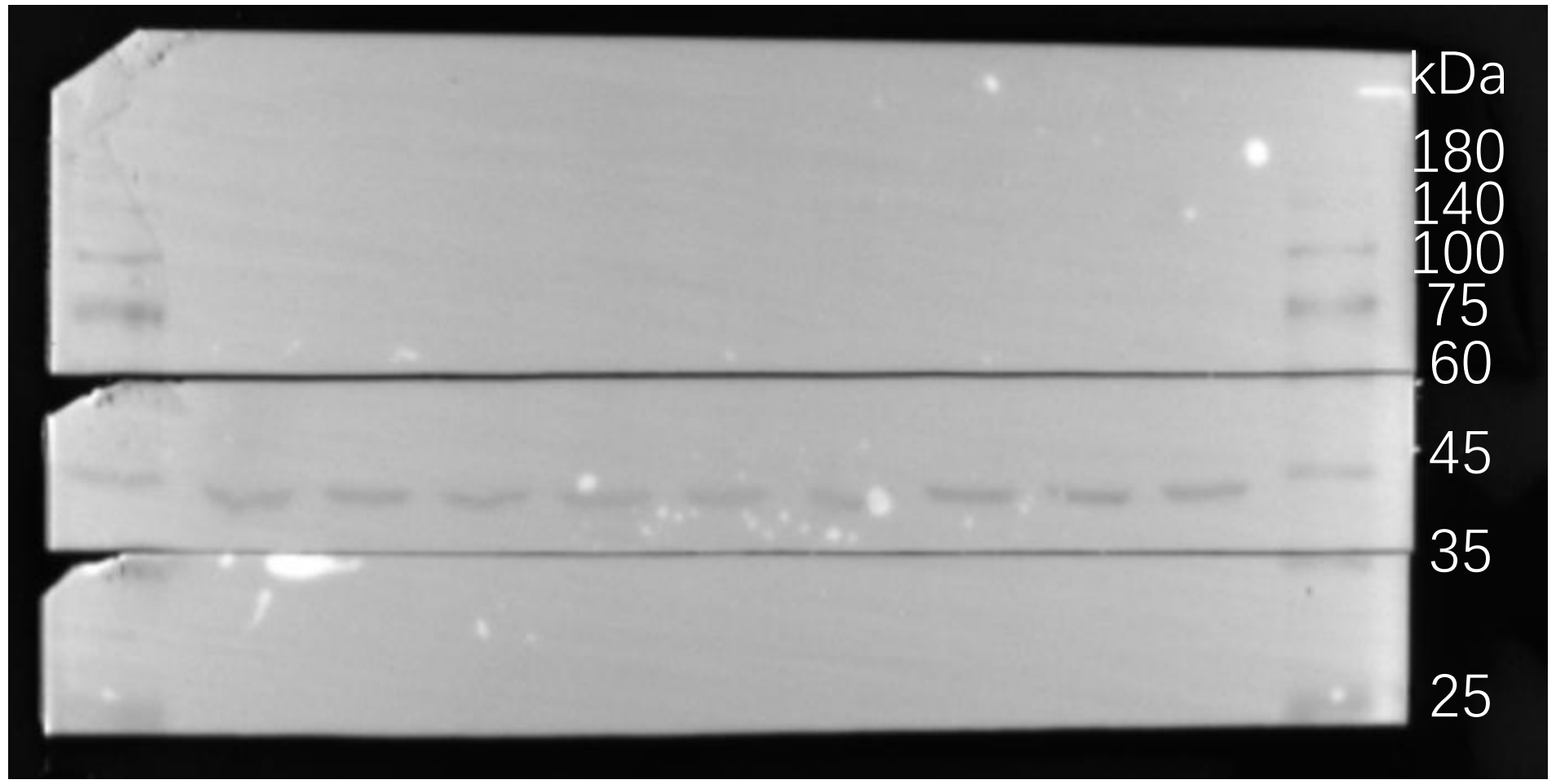

Figure 3J

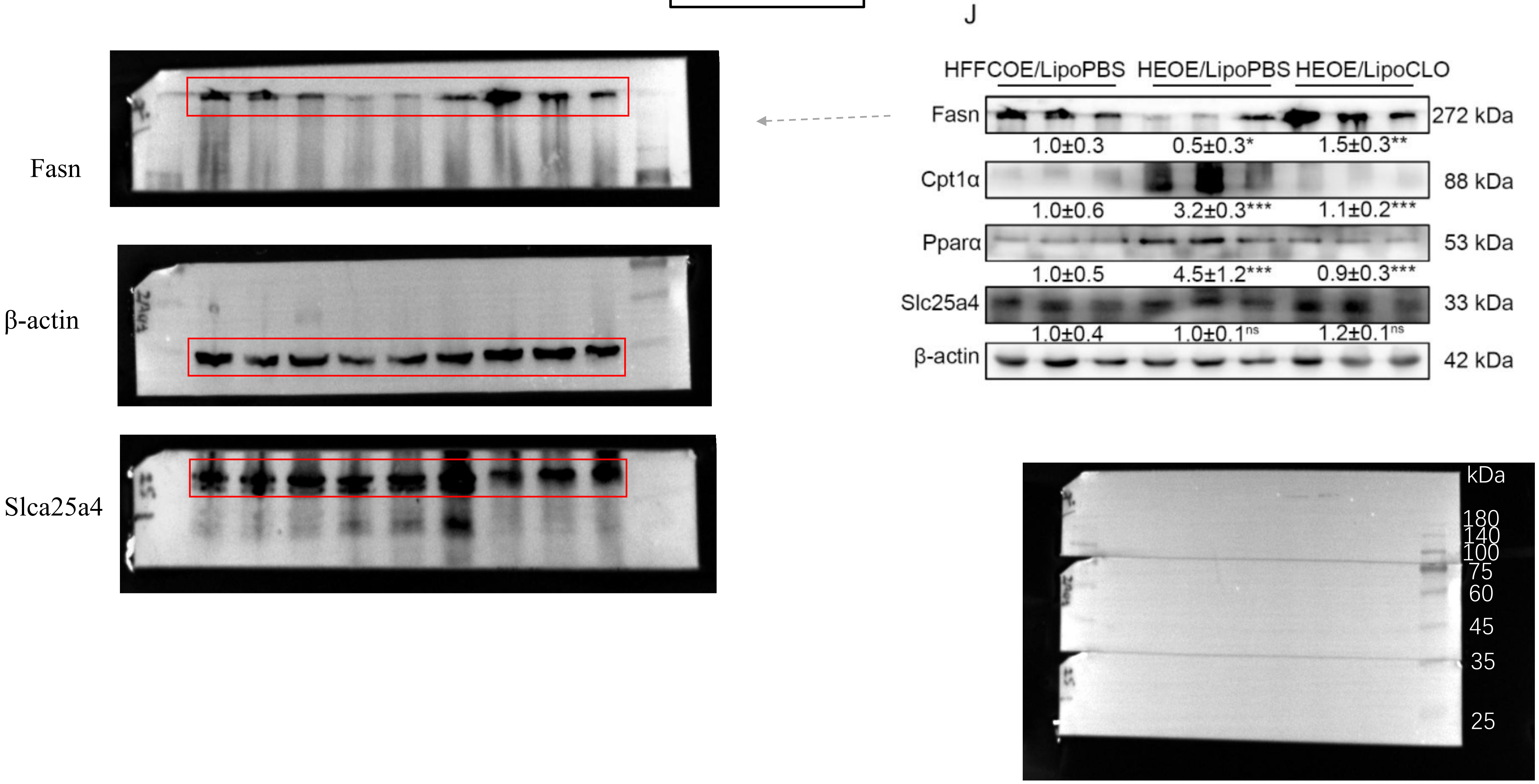

Fasn
β-actin
Slca25a4
J
HFFCOE/LipoPBS
HEOE/LipoPBS
HEOE/LipoCLO
Fasn
272 kDa
1.0±0.3
0.5±0.3*
1.5±0.3**
Cpt1α
88 kDa
1.0±0.6
3.2±0.3***
1.1±0.2***
Pparα
53 kDa
1.0±0.5
4.5±1.2***
0.9±0.3***
Slc25a4
33 kDa
1.0±0.4
1.0±0.1ns
1.2±0.1ns
β-actin
42 kDa
kDa
180
140
100
75
60
45
35
25

Figure 3J

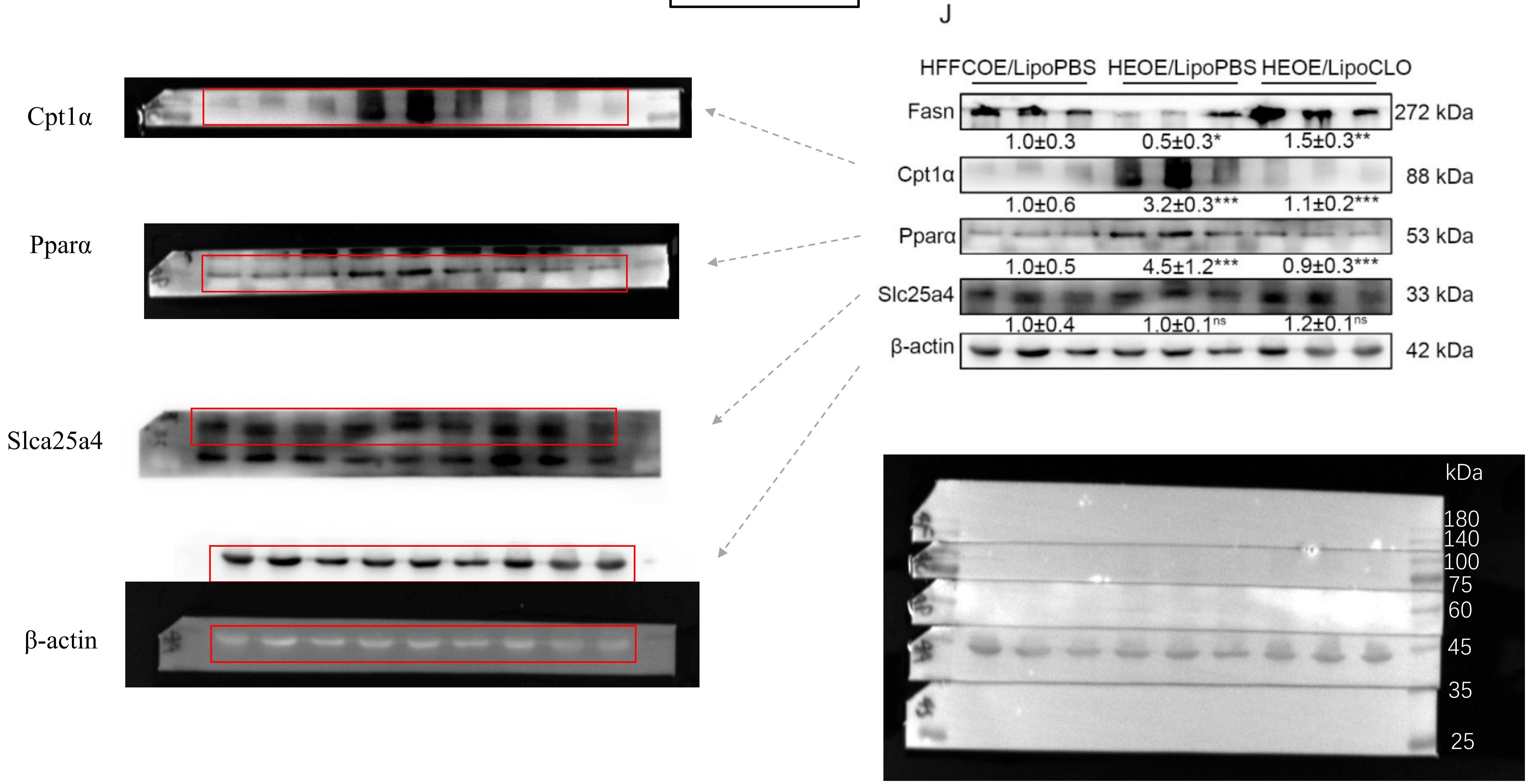

J
HFFCOE/LipoPBS
HEOE/LipoPBS
HEOE/LipoCLO
Fasn
272 kDa
1.0±0.3
0.5±0.3*
1.5±0.3**
Cpt1α
88 kDa
1.0±0.6
3.2±0.3***
1.1±0.2***
Pparα
53 kDa
1.0±0.5
4.5±1.2***
0.9±0.3***
Slc25a4
33 kDa
1.0±0.4
1.0±0.1ns
1.2±0.1ns
β-actin
42 kDa
Cpt1α
Pparα
Slca25a4
β-actin
kDa
180
140
100
75
60
45
35
25

Figure 4I

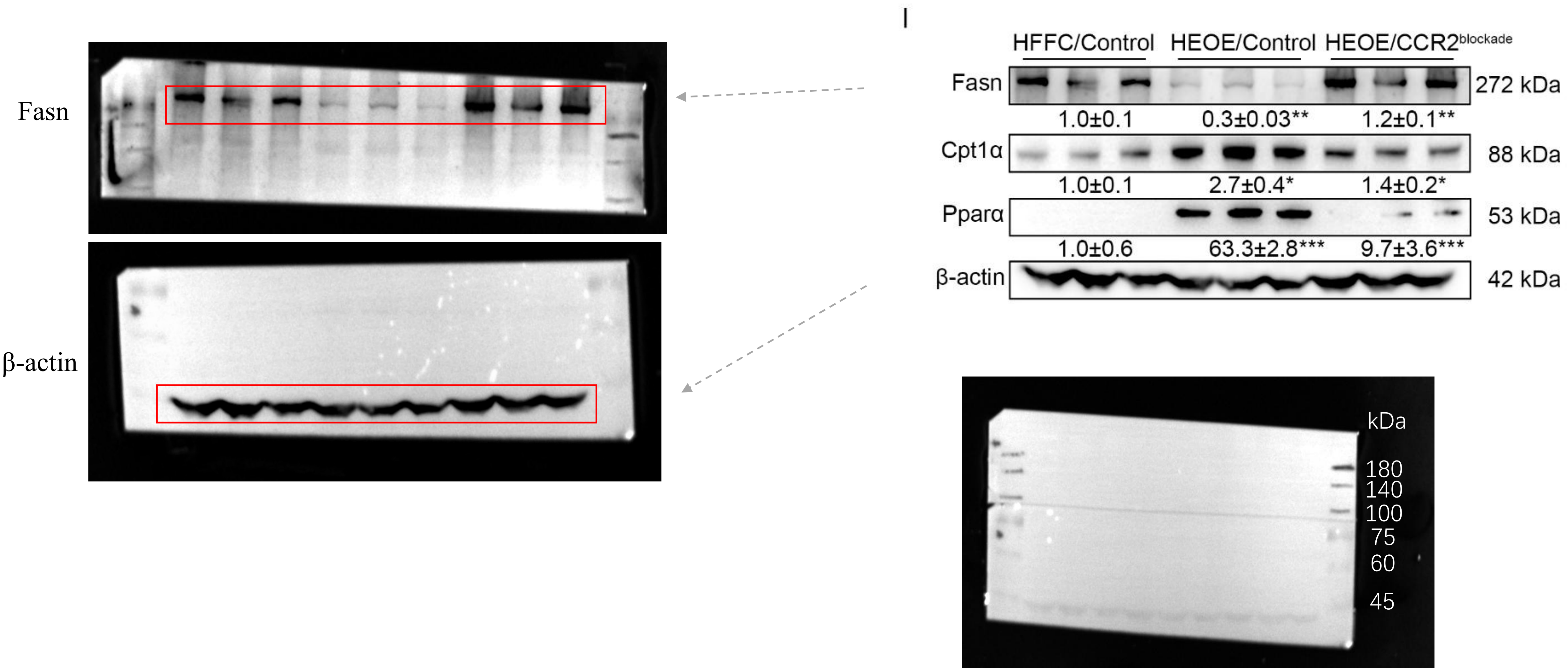

Fasn
β-actin
I
HFFC/Control
HEOE/Control
HEOE/CCR2blockade
Fasn
272 kDa
1.0±0.1
0.3±0.03**
1.2±0.1**
Cpt1α
88 kDa
1.0±0.1
2.7±0.4*
1.4±0.2*
Pparα
53 kDa
1.0±0.6
63.3±2.8***
9.7±3.6***
β-actin
42 kDa
kDa
180
140
100
75
60
45

Figure 4I

Cpt1α

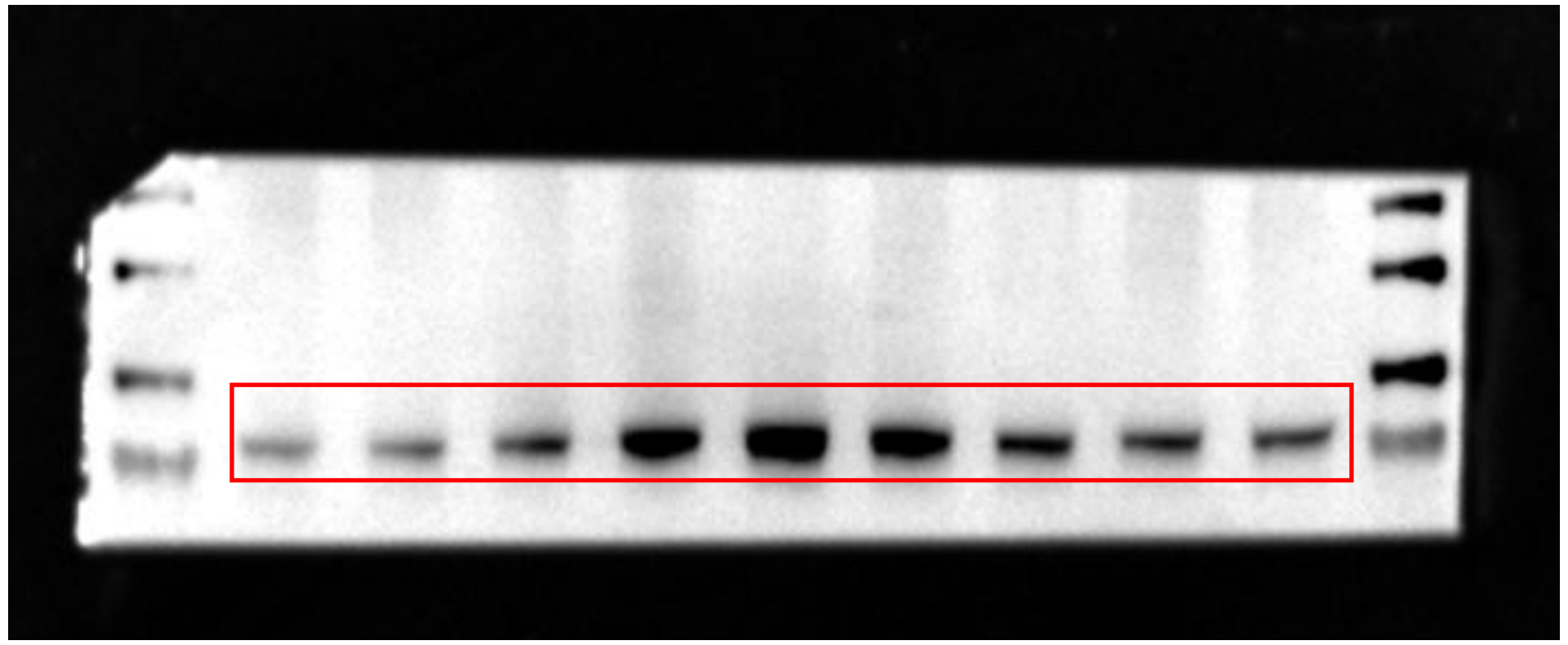

β-actin

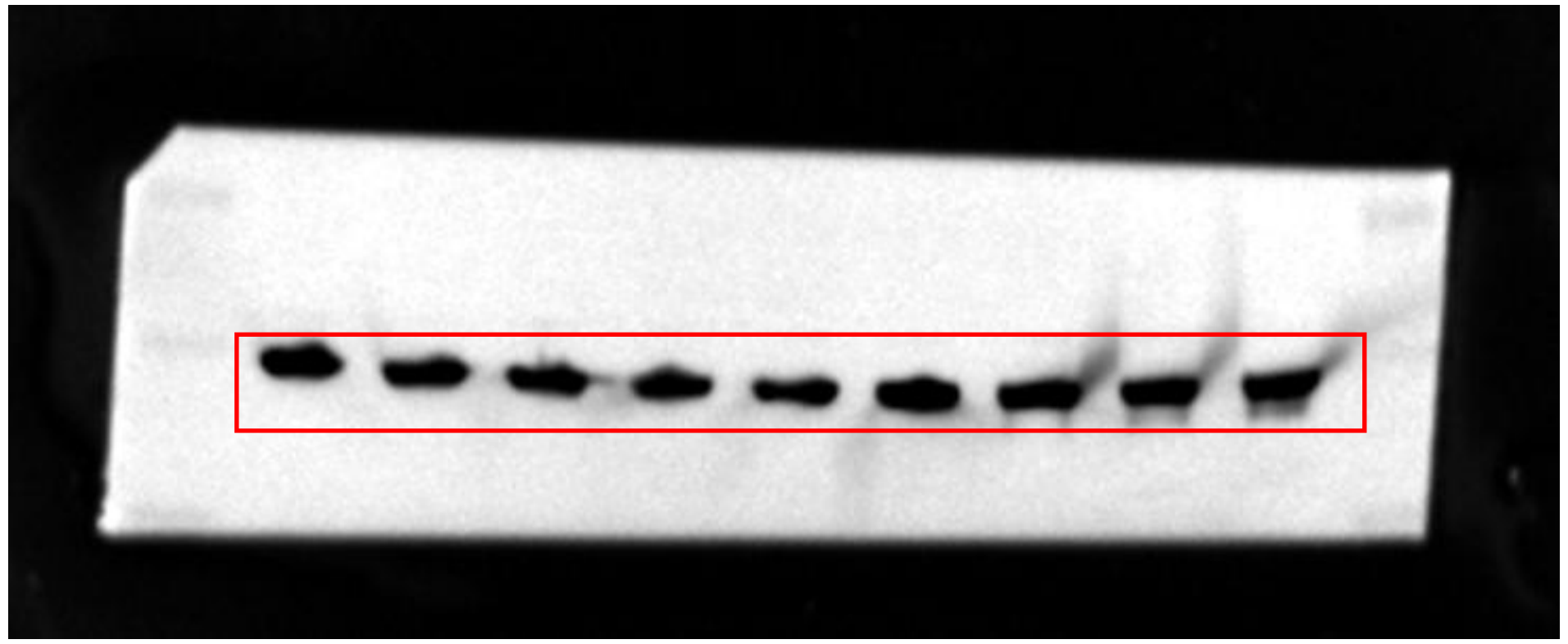

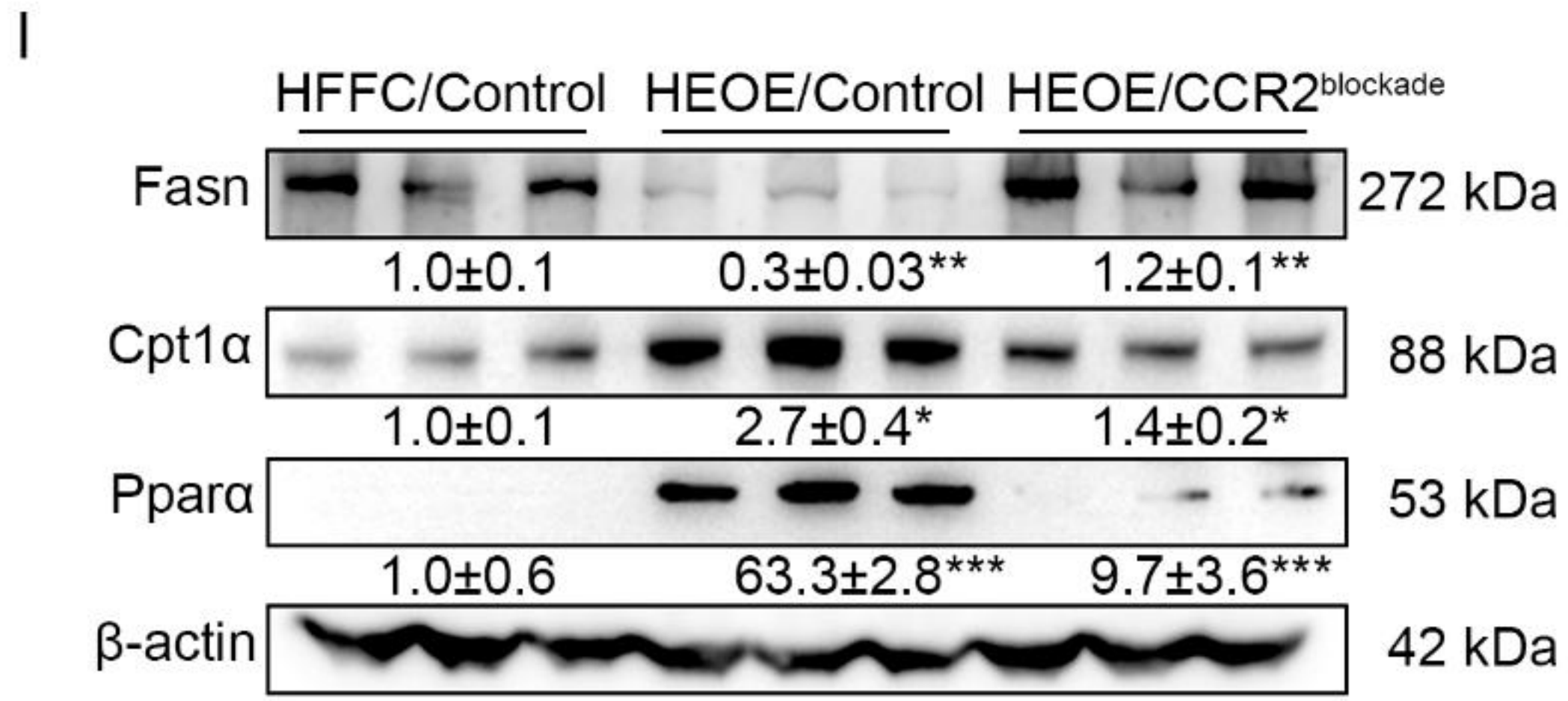

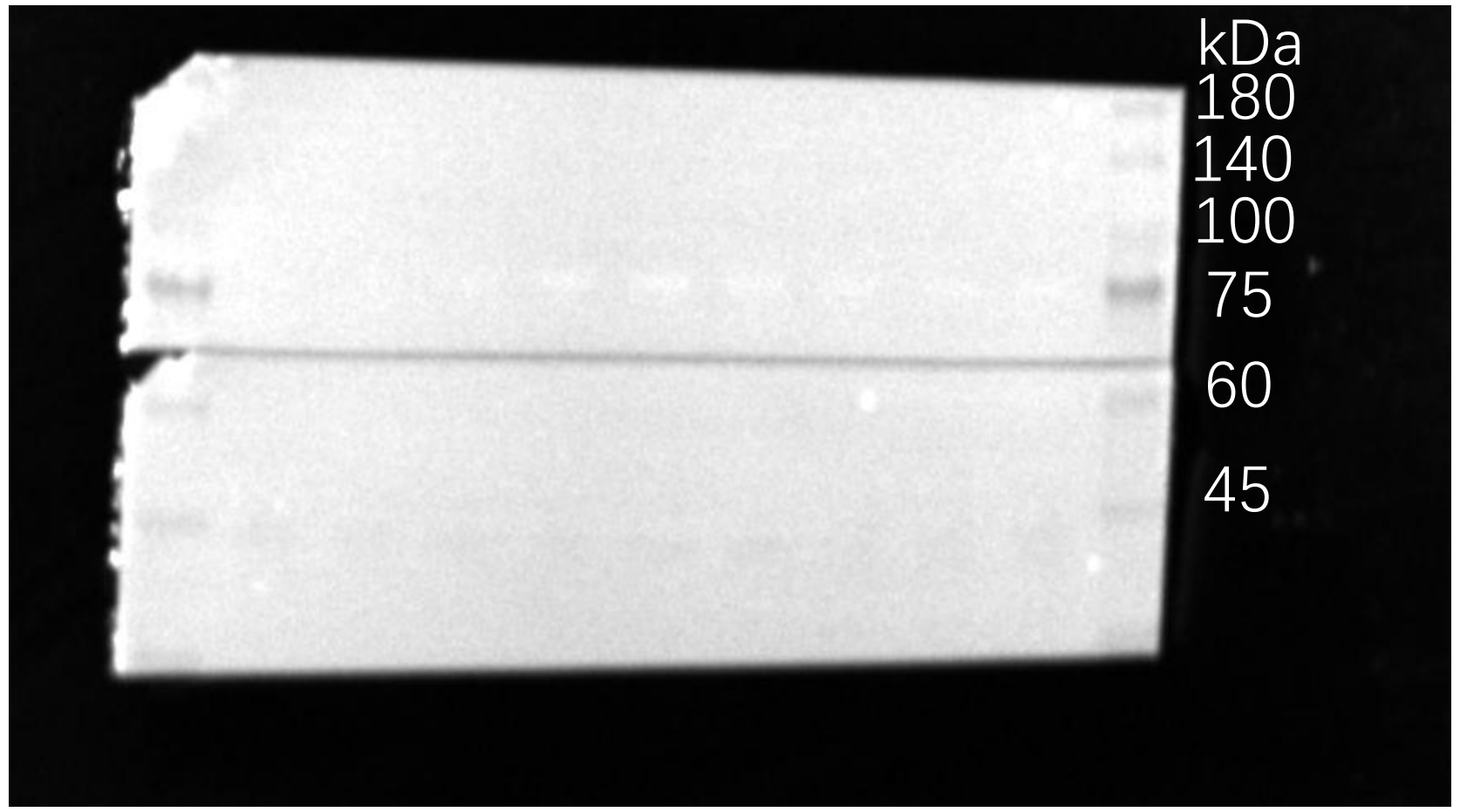

Figure 4I

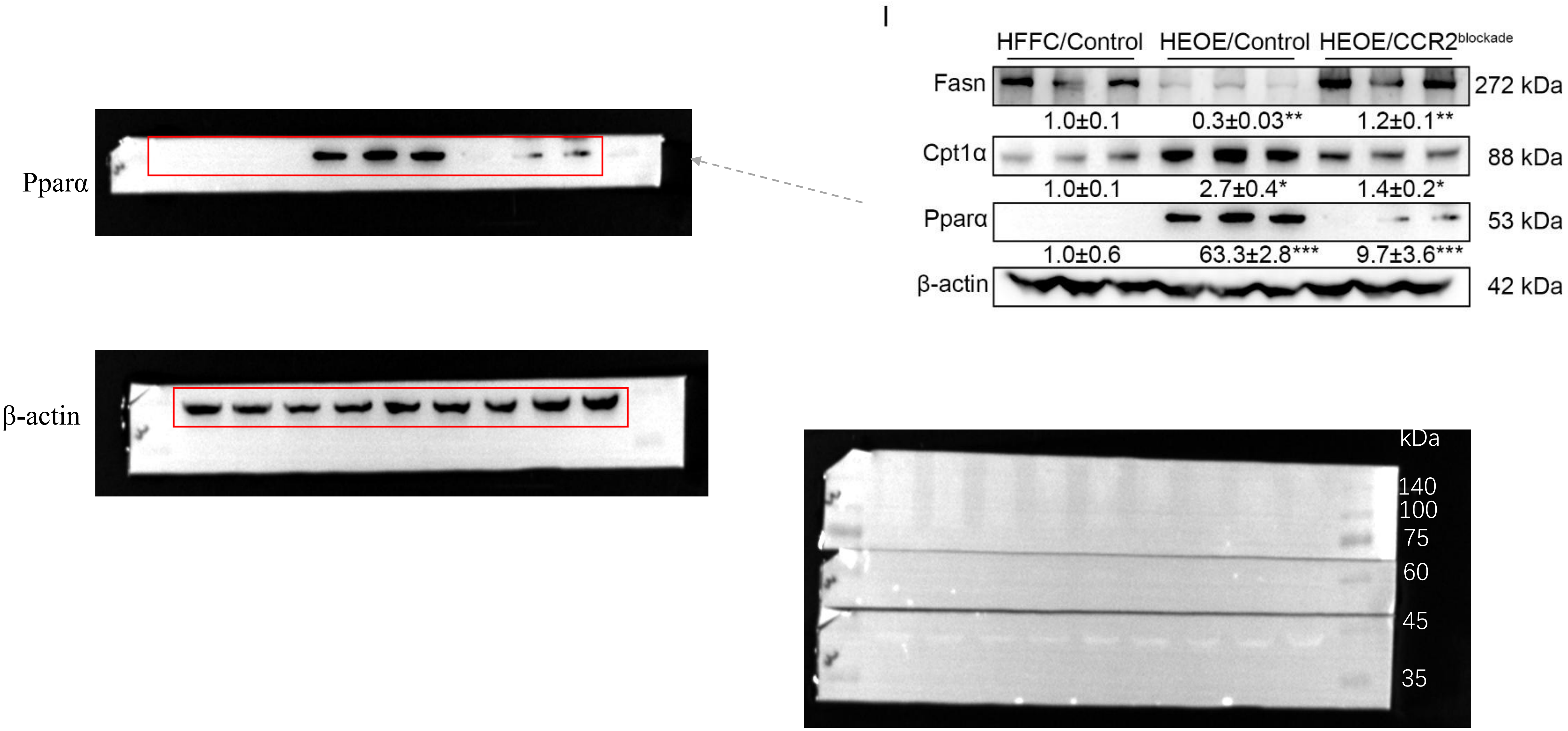

Pparα
β-actin
I
HFFC/Control
HEOE/Control
HEOE/CCR2blockade
Fasn
272 kDa
1.0±0.1
0.3±0.03**
1.2±0.1**
Cpt1α
88 kDa
1.0±0.1
2.7±0.4*
1.4±0.2*
Pparα
53 kDa
1.0±0.6
63.3±2.8***
9.7±3.6***
β-actin
42 kDa
kDa
140
100
75
60
45
35

Figure 6B

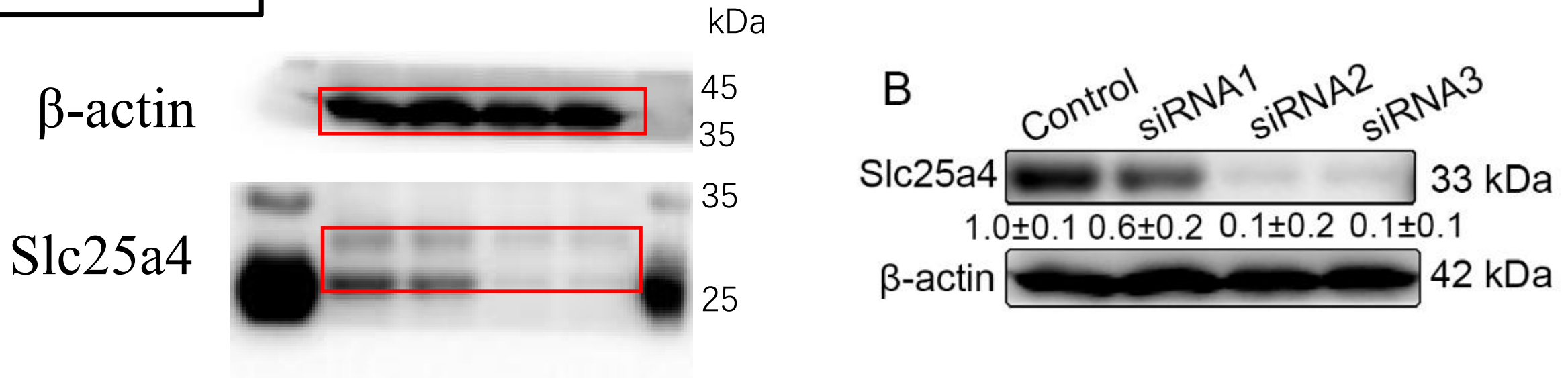

Figure 6C

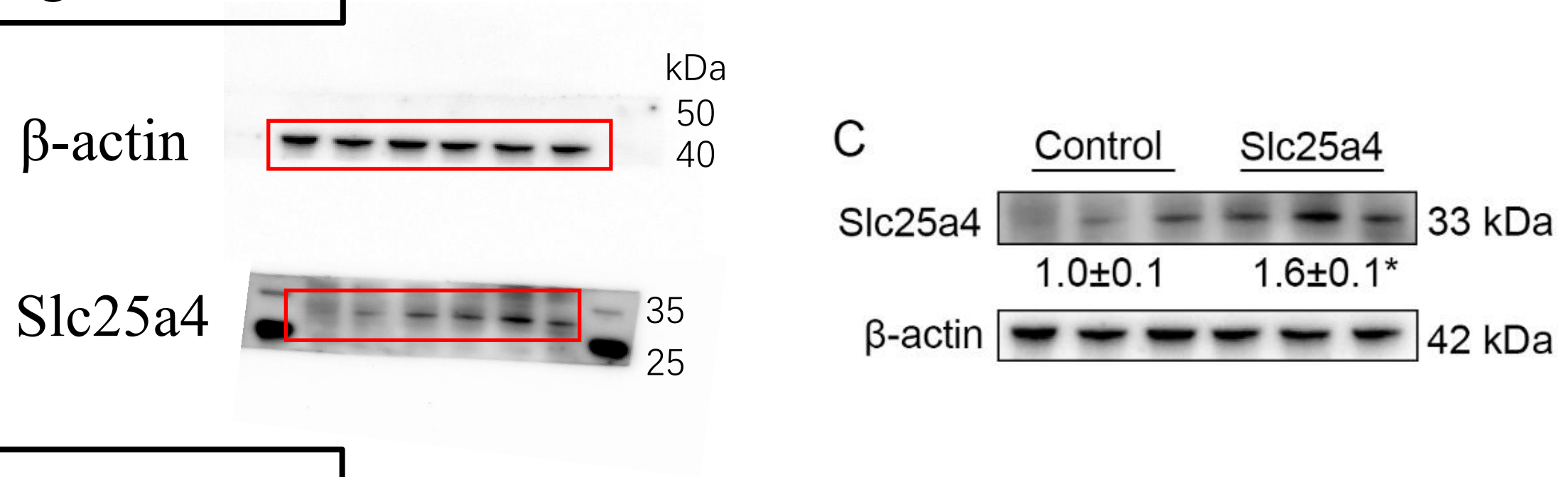

Figure 6F

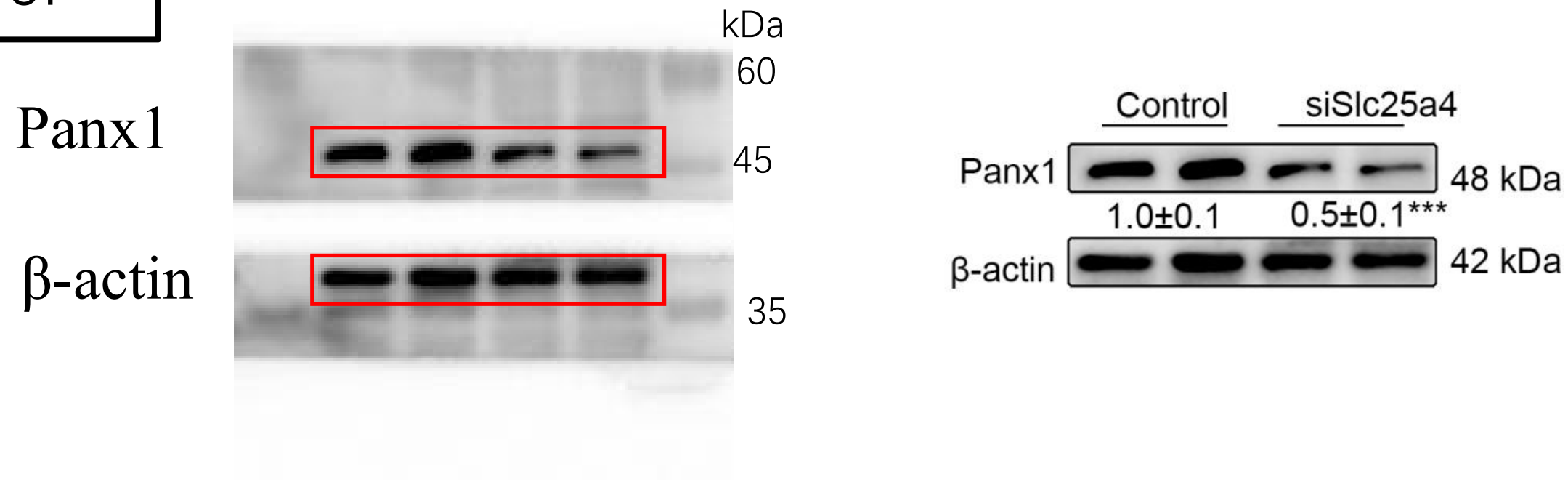

Figure 6F

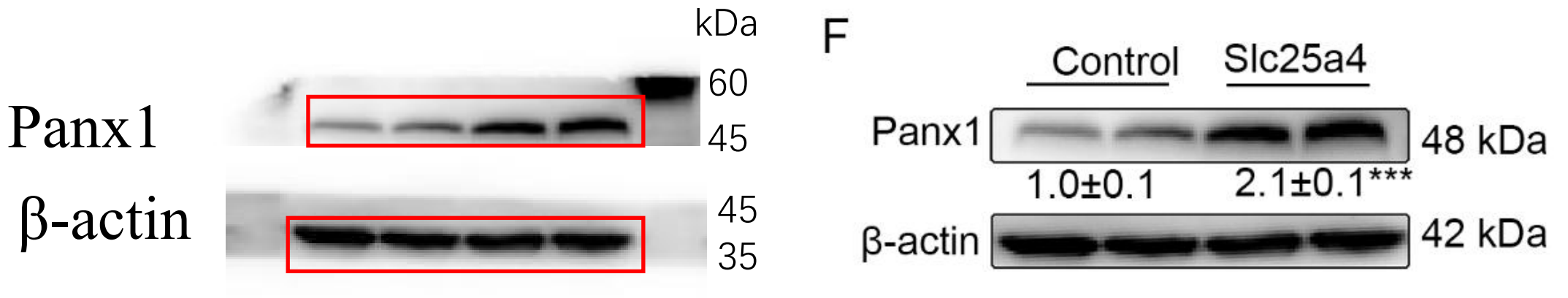

Figure 6G

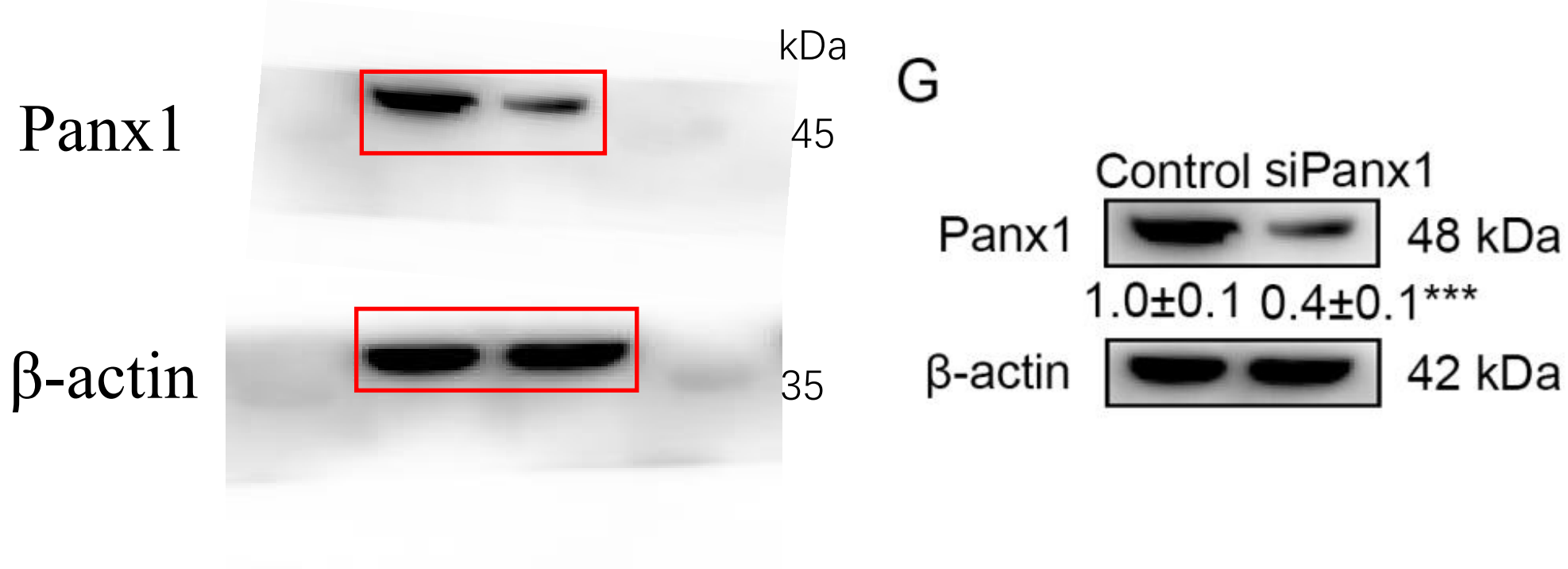

Figure 7D

Fasn

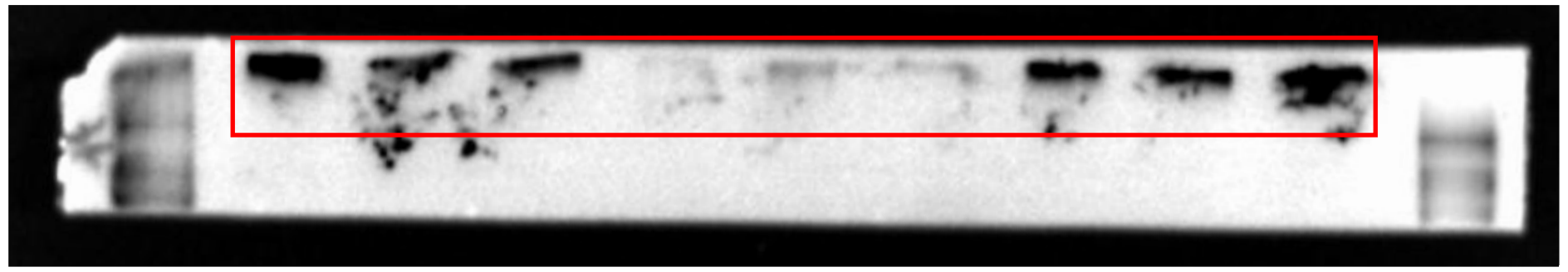

β-actin

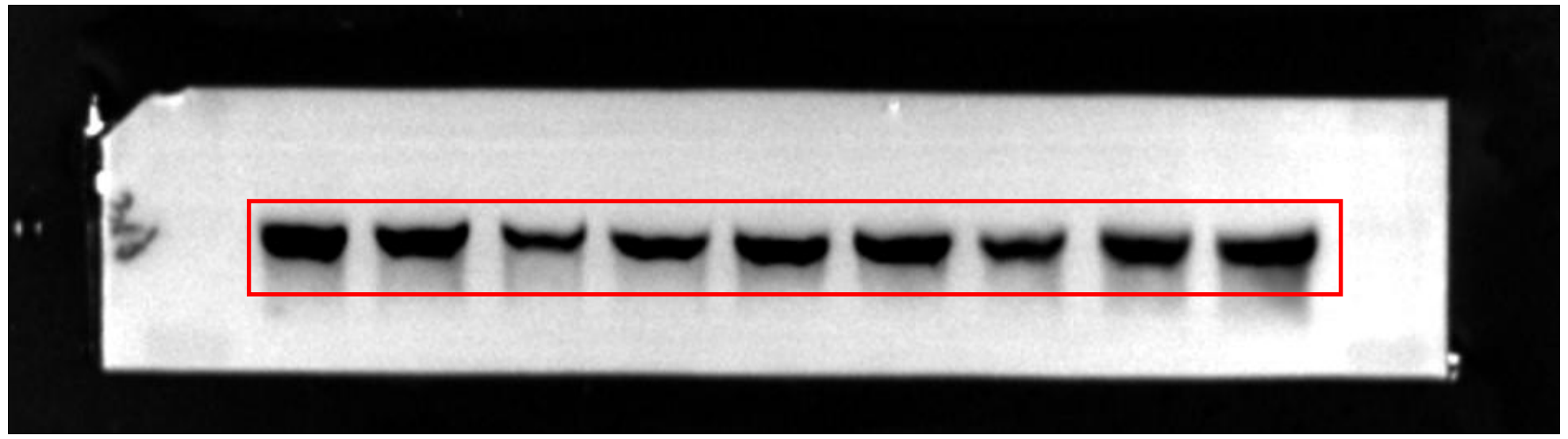

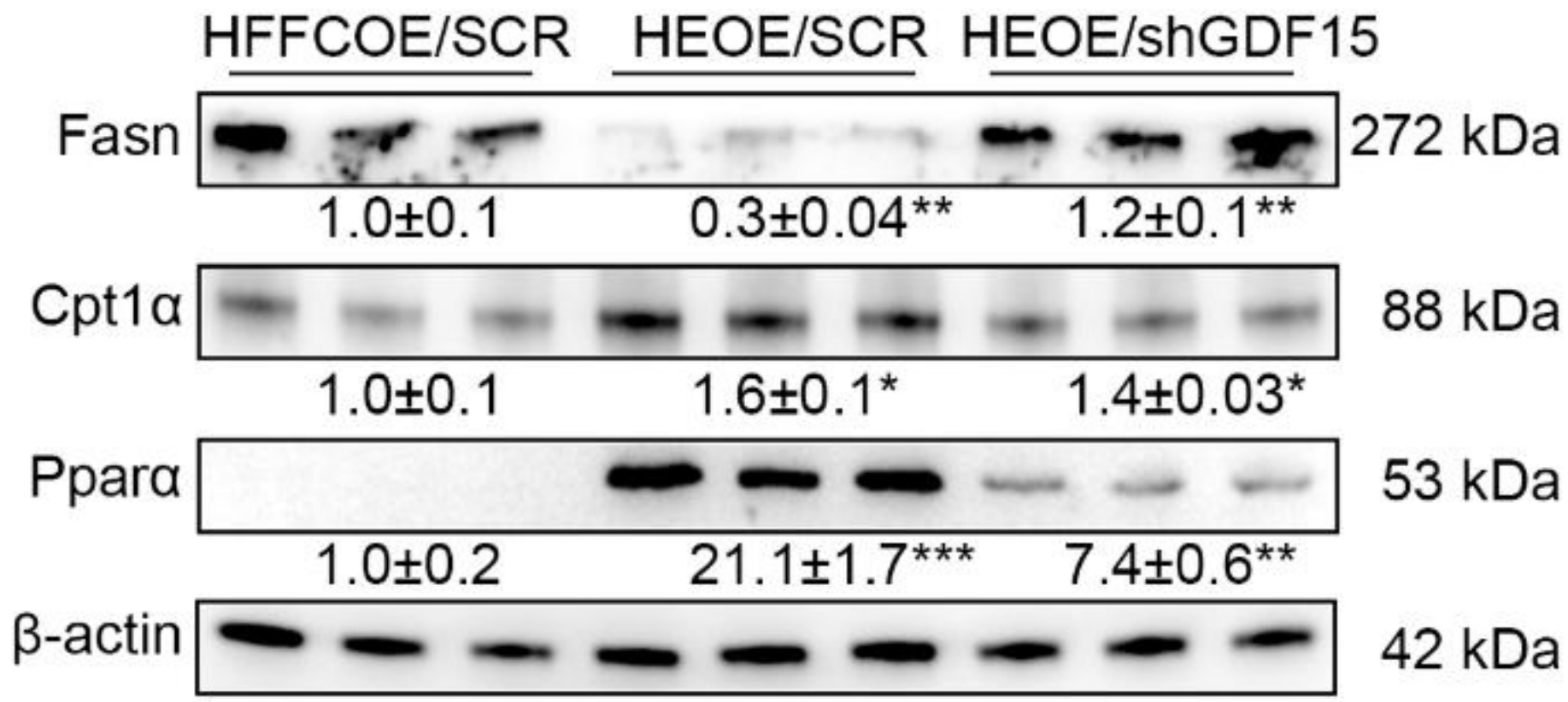

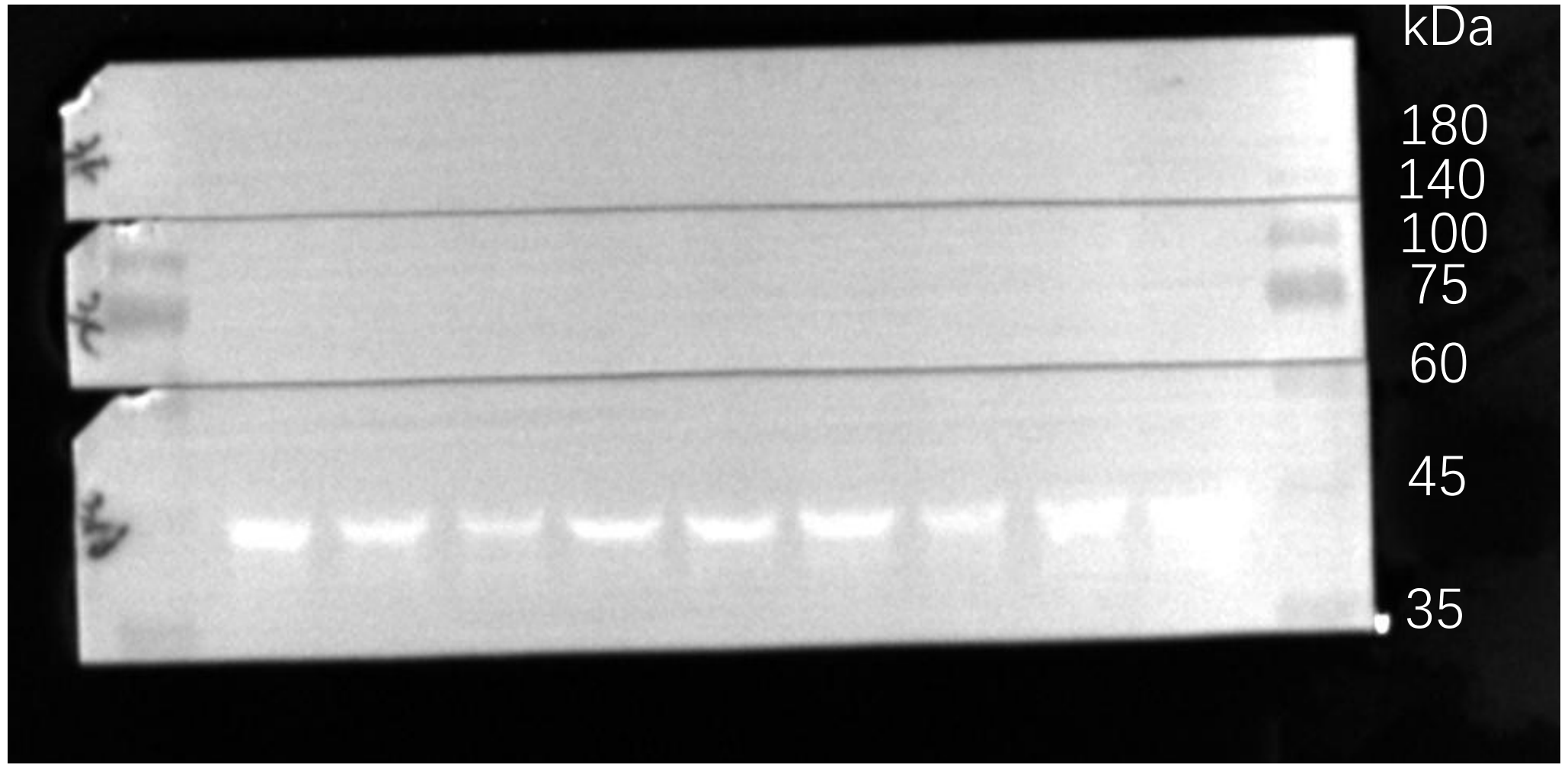

Figure 7D

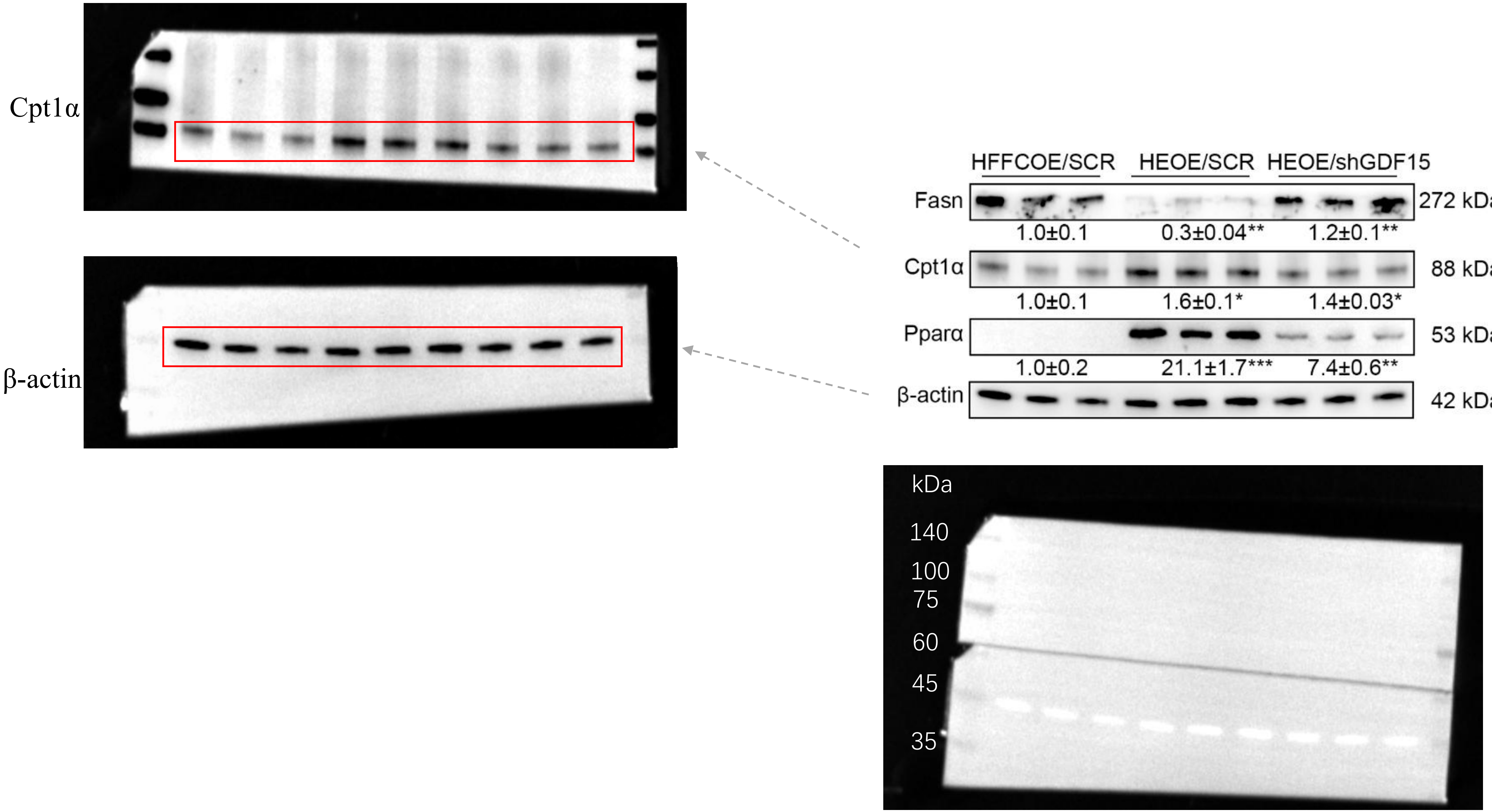

Cpt1α
β-actin
HFFCOE/SCR
HEOE/SCR
HEOE/shGDF15
Fasn
272 kDa
1.0±0.1
0.3±0.04**
1.2±0.1**
Cpt1α
88 kDa
1.0±0.1
1.6±0.1*
1.4±0.03*
Pparα
53 kDa
1.0±0.2
21.1±1.7***
7.4±0.6**
β-actin
42 kDa
kDa
140
100
75
60
45
35

Figure 7D

Pparα

β-actin

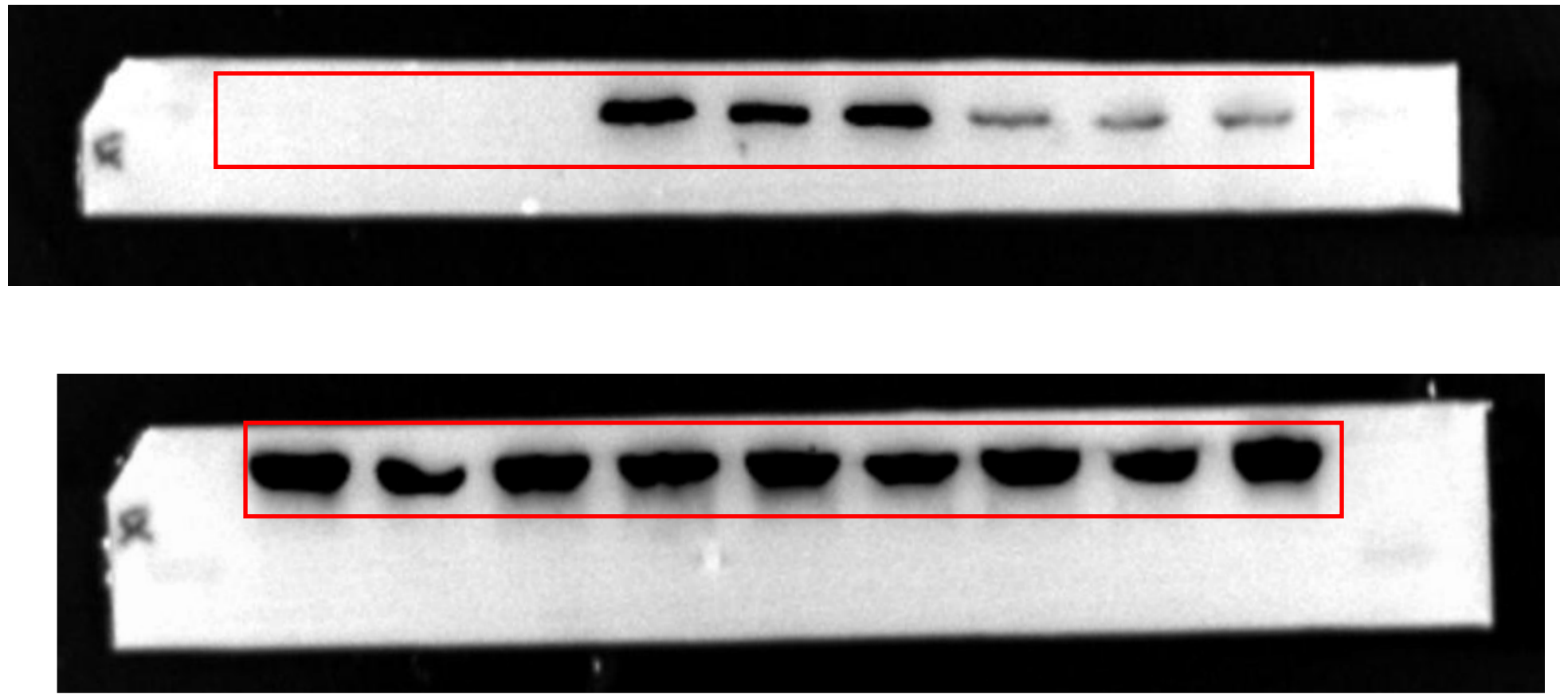

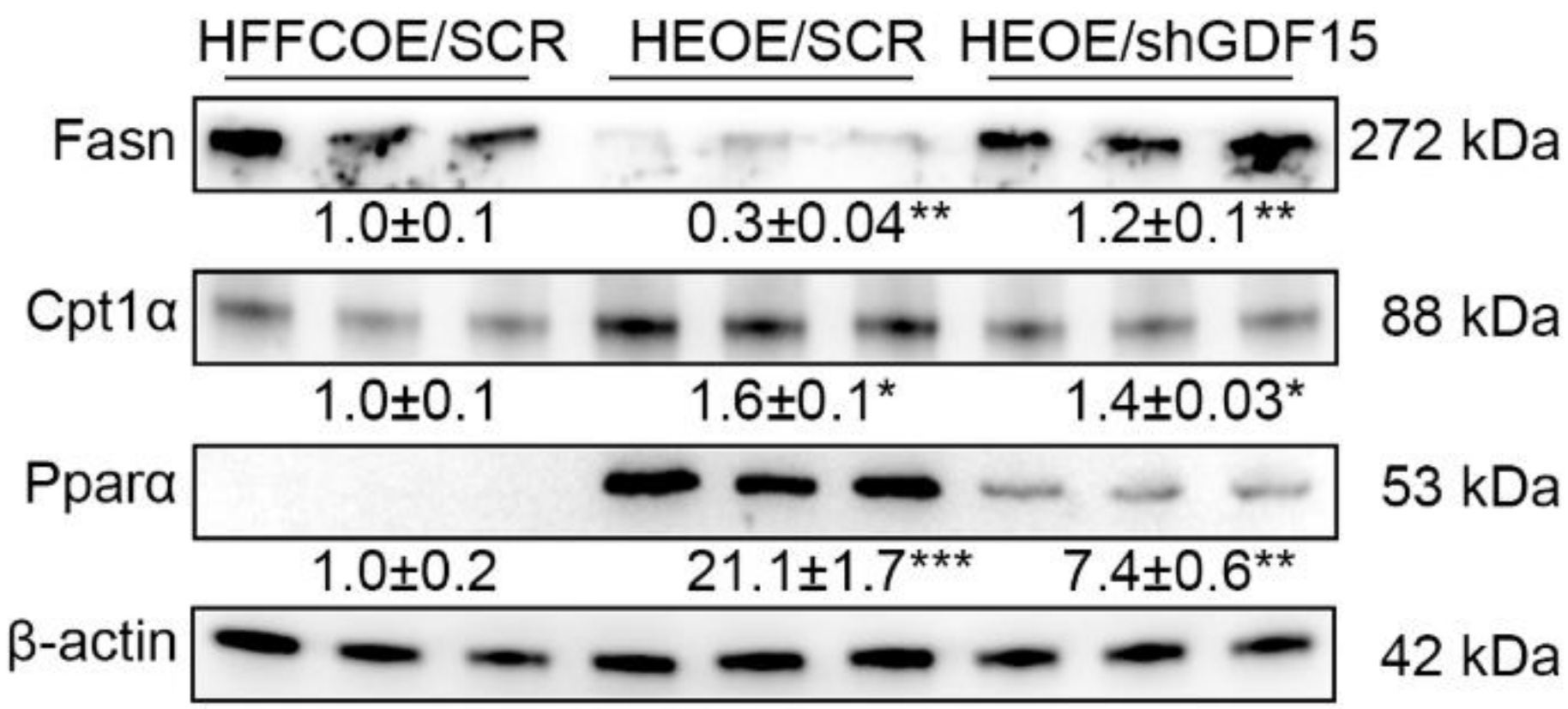

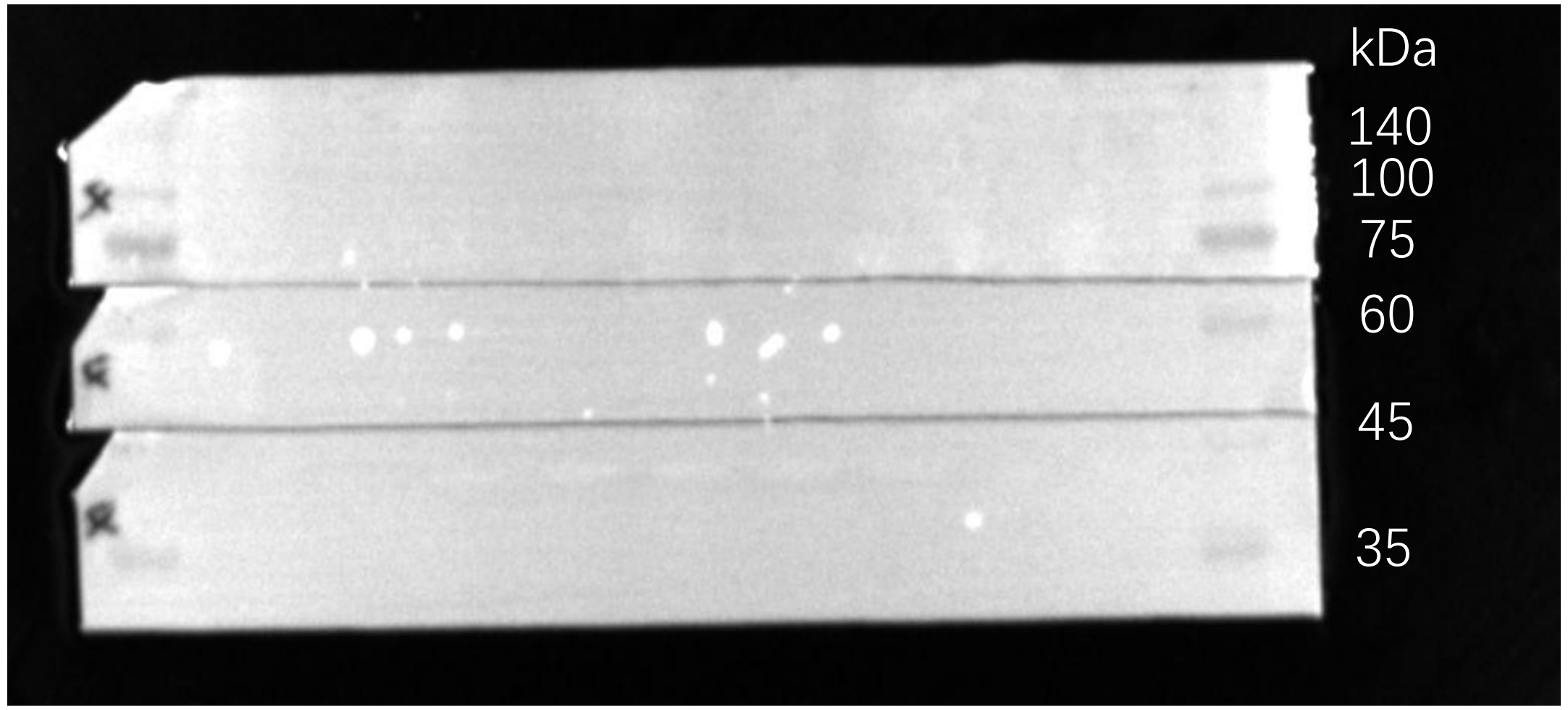

Figure 7J

Fasn

β-actin

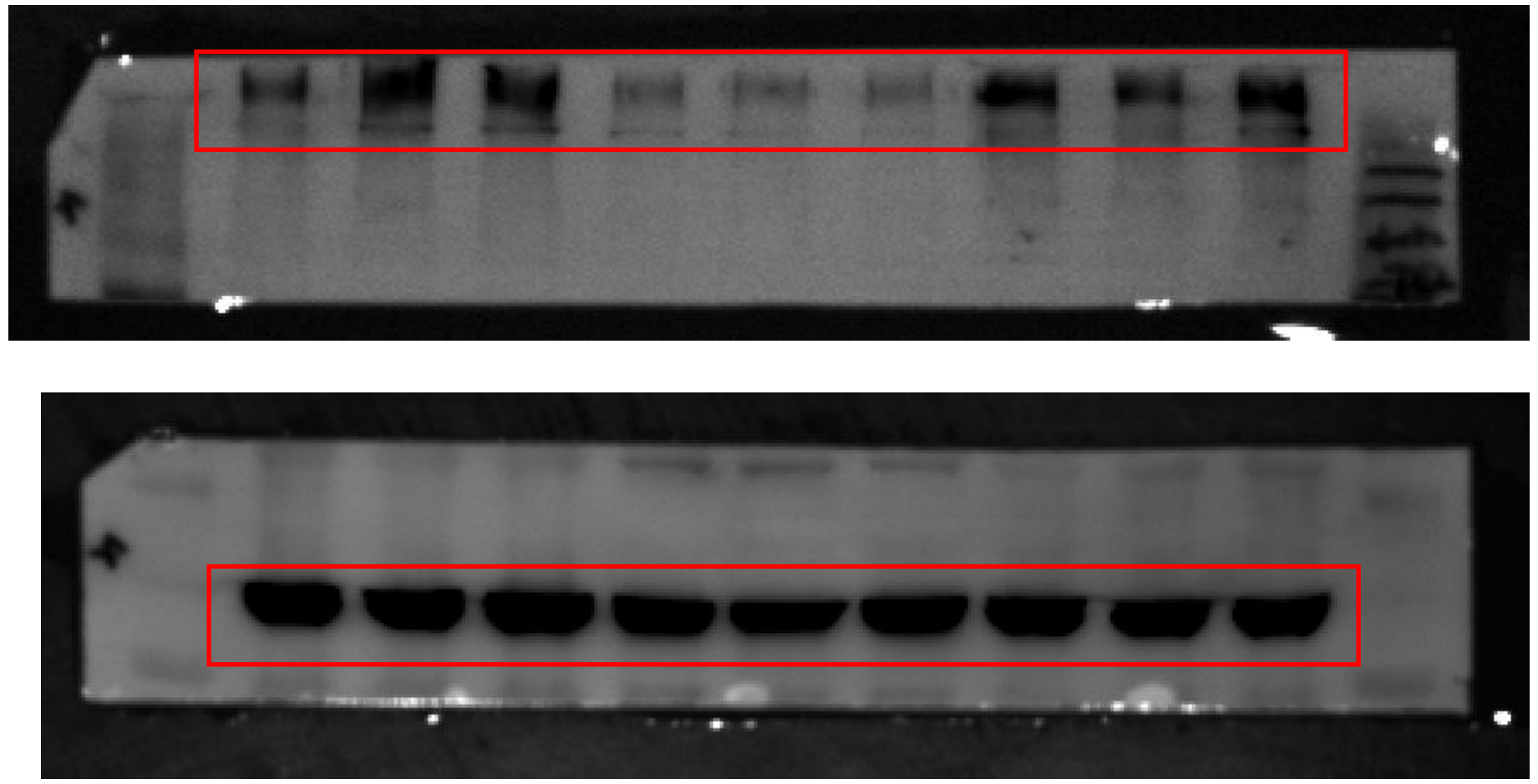

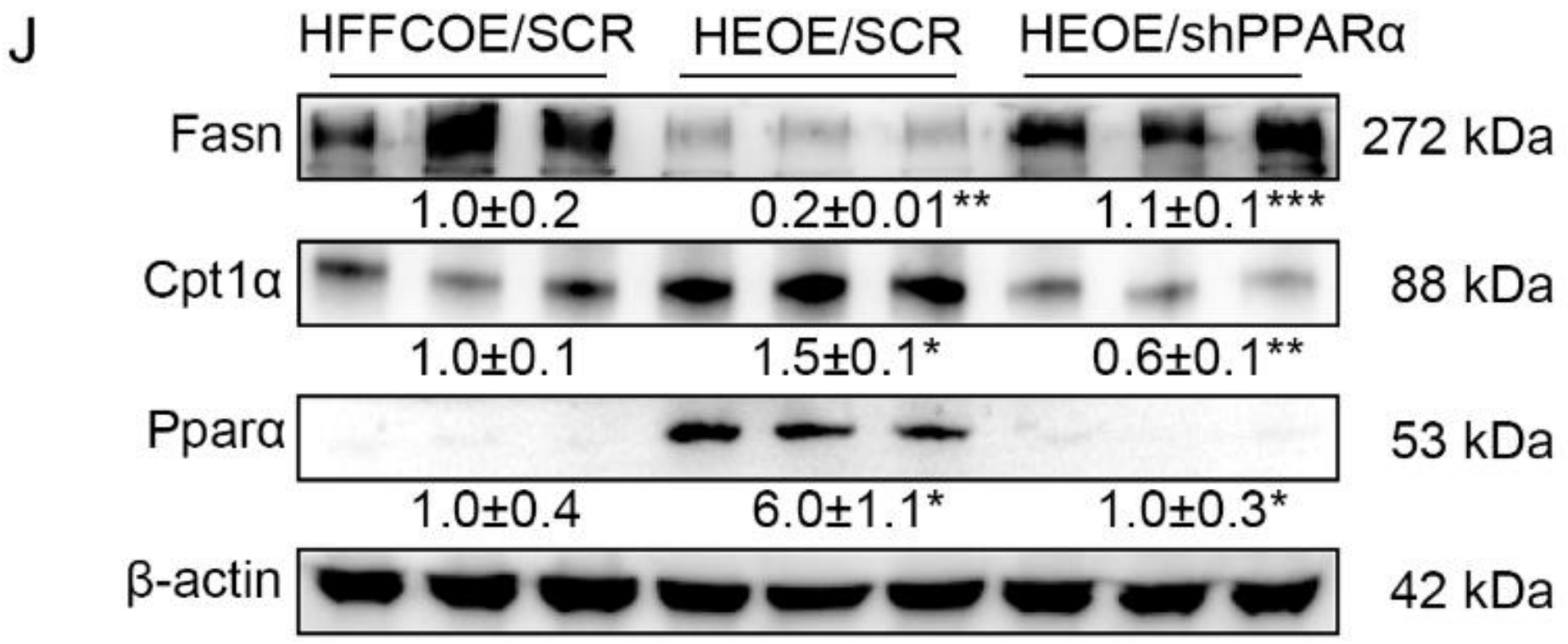

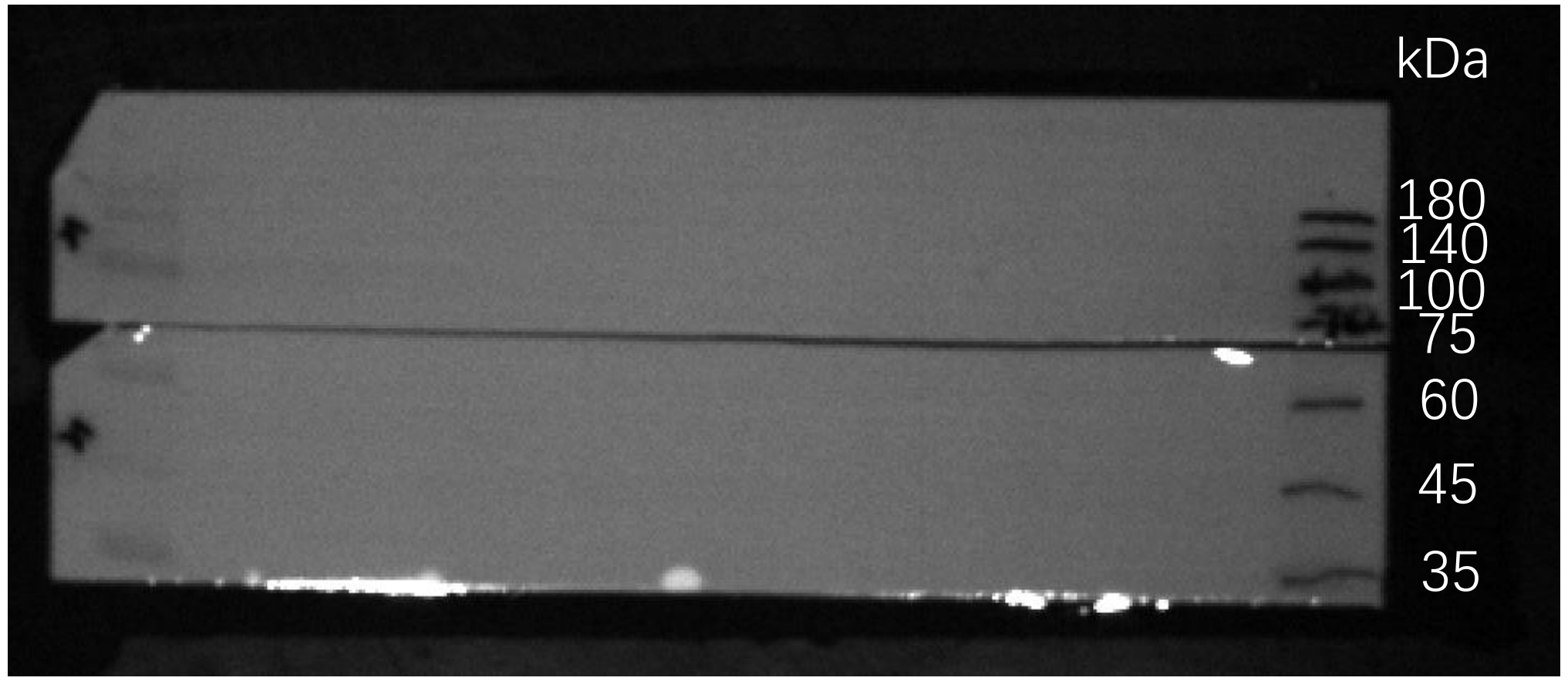

Figure 7J

Cpt1α

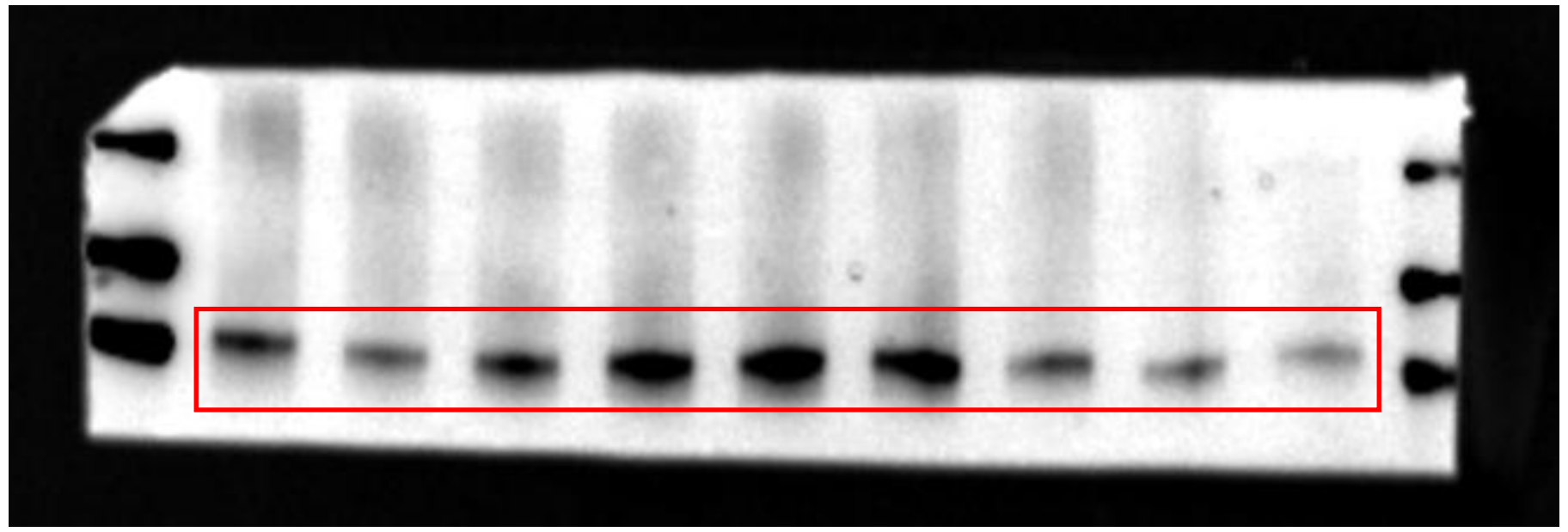

β-actin

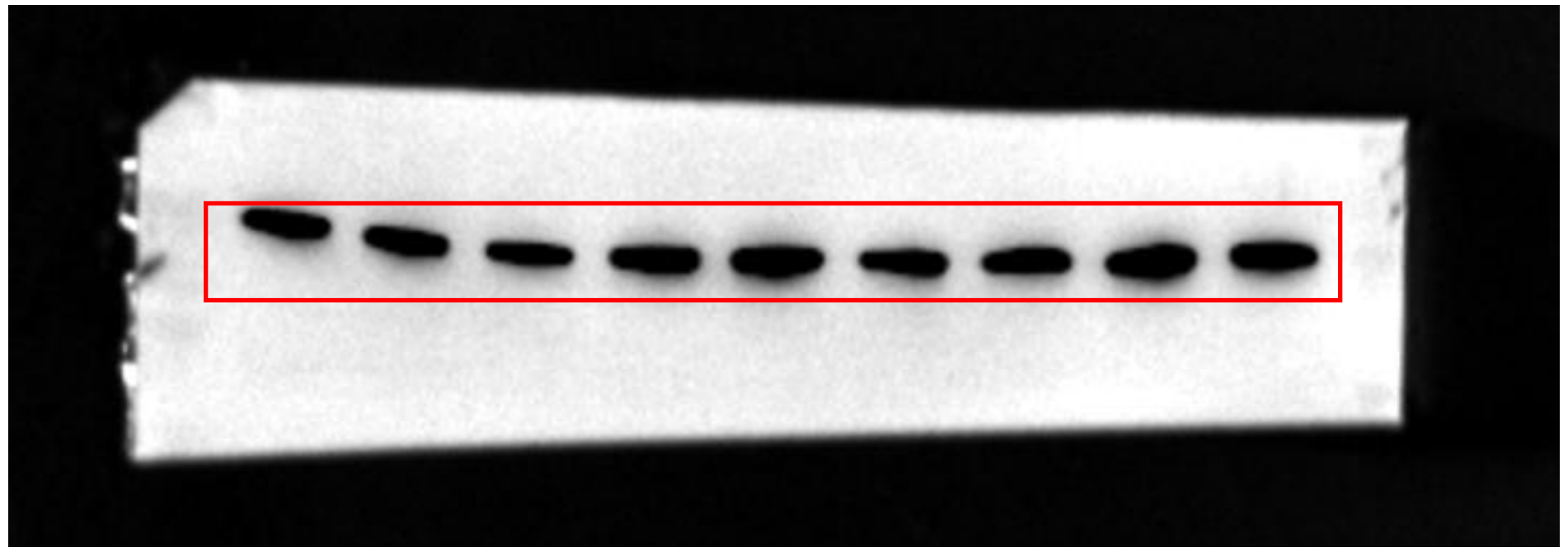

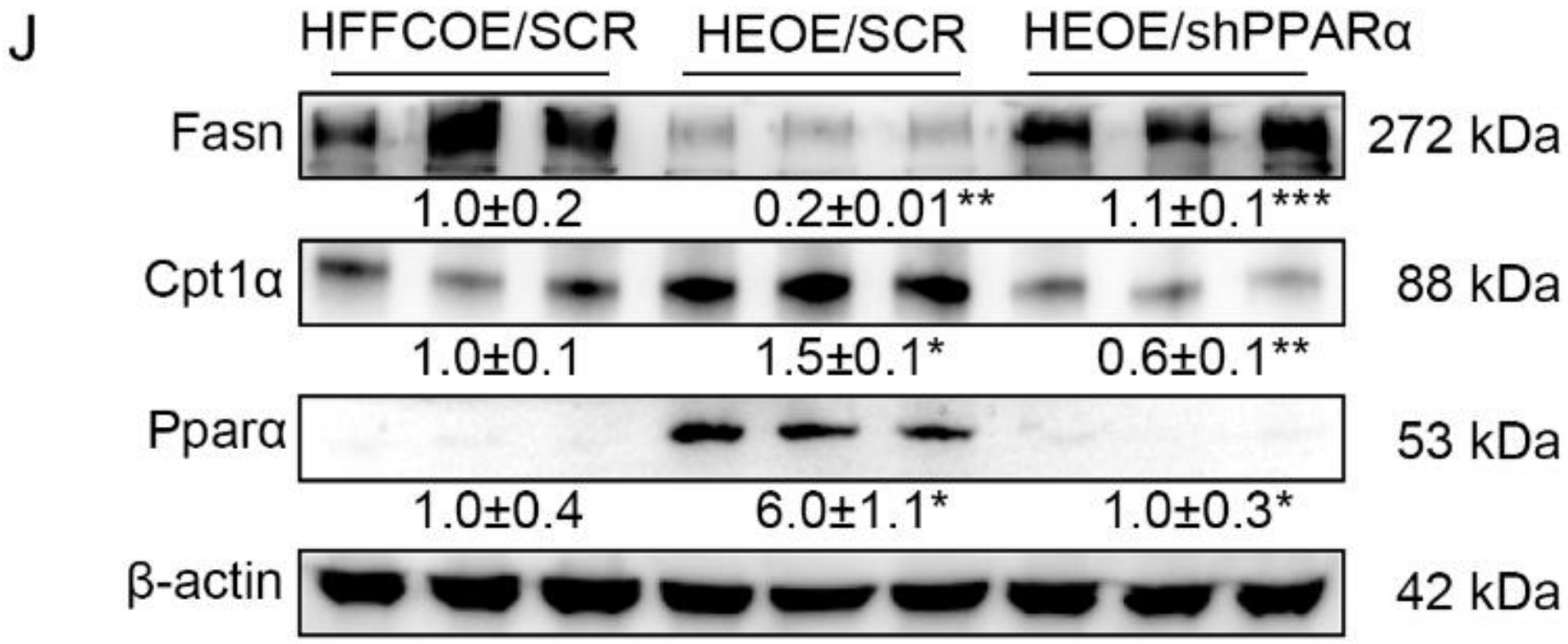

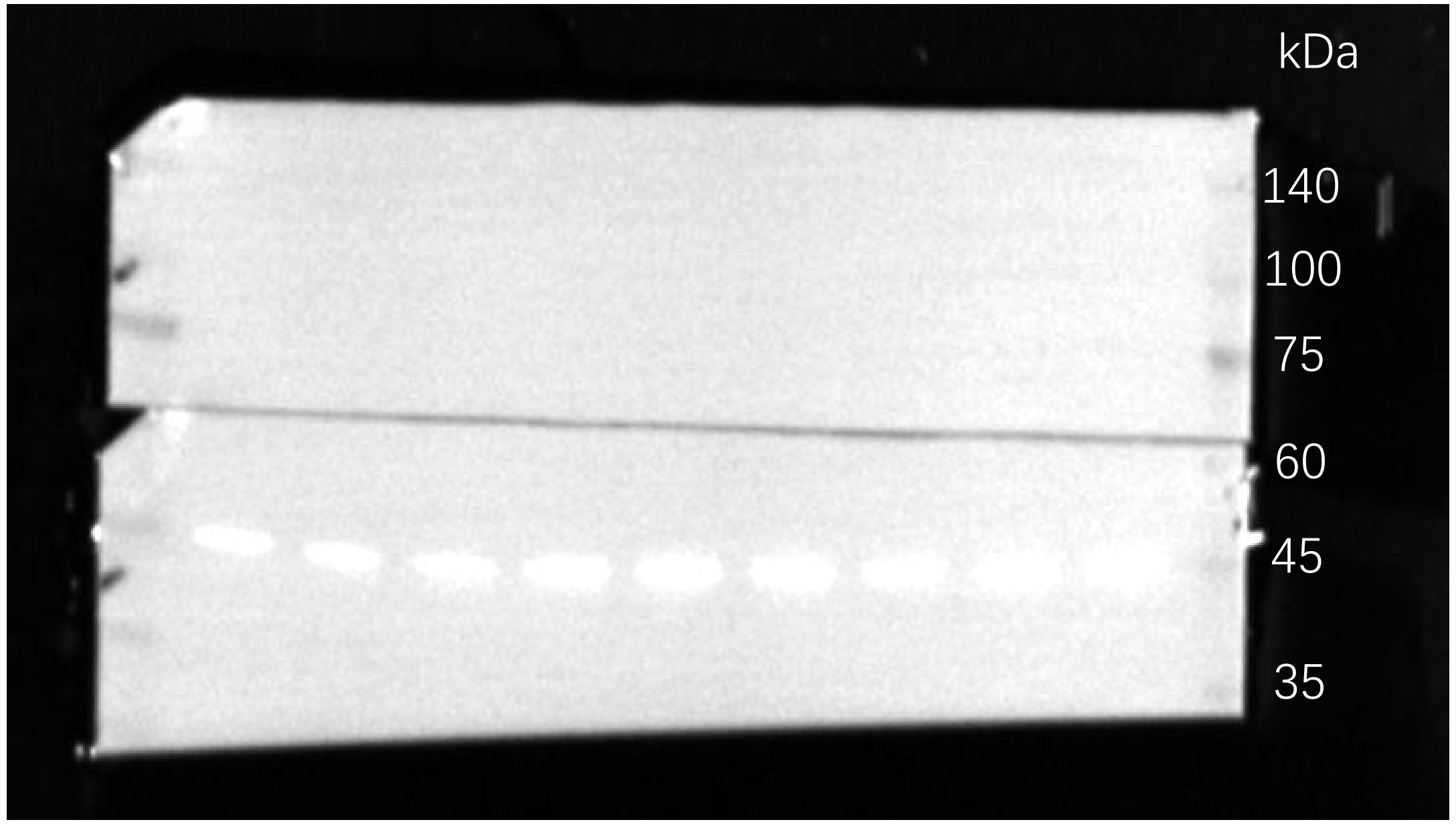

Figure 7J

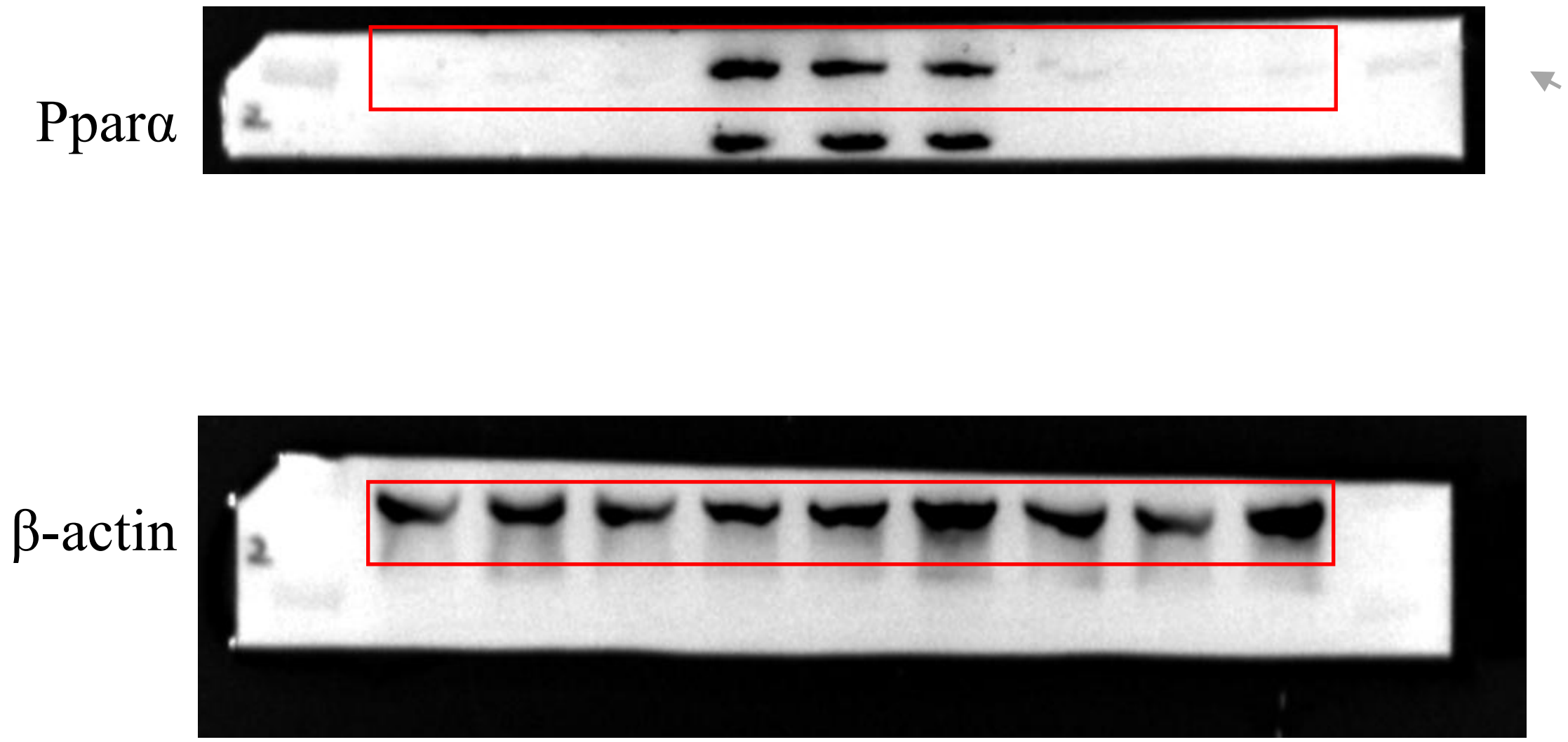

Pparα
β-actin

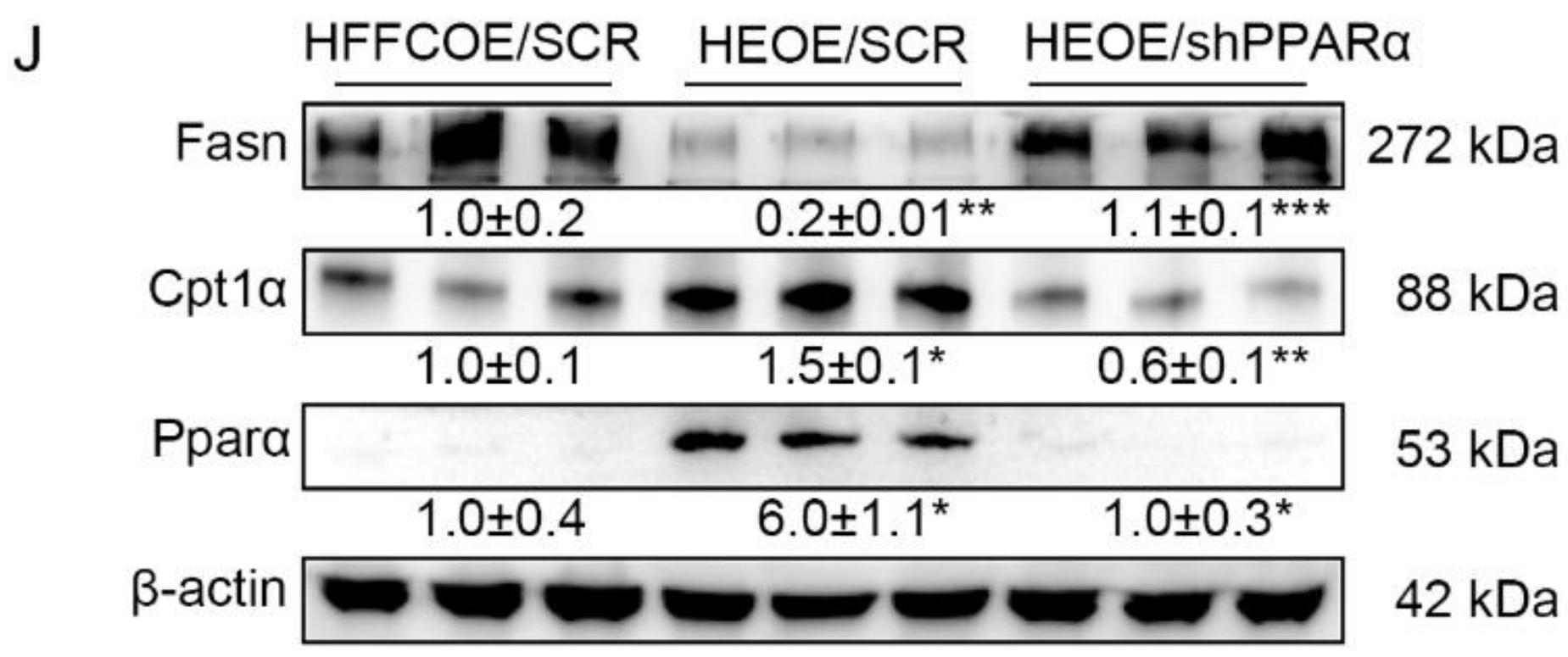

J
HFFCOE/SCR
HEOE/SCR
HEOE/shPPARα
Fasn
272 kDa
1.0±0.2
0.2±0.01**
1.1±0.1***
Cpt1α
88 kDa
1.0±0.1
1.5±0.1*
0.6±0.1**
Pparα
53 kDa
1.0±0.4
6.0±1.1*
1.0±0.3*
β-actin
42 kDa

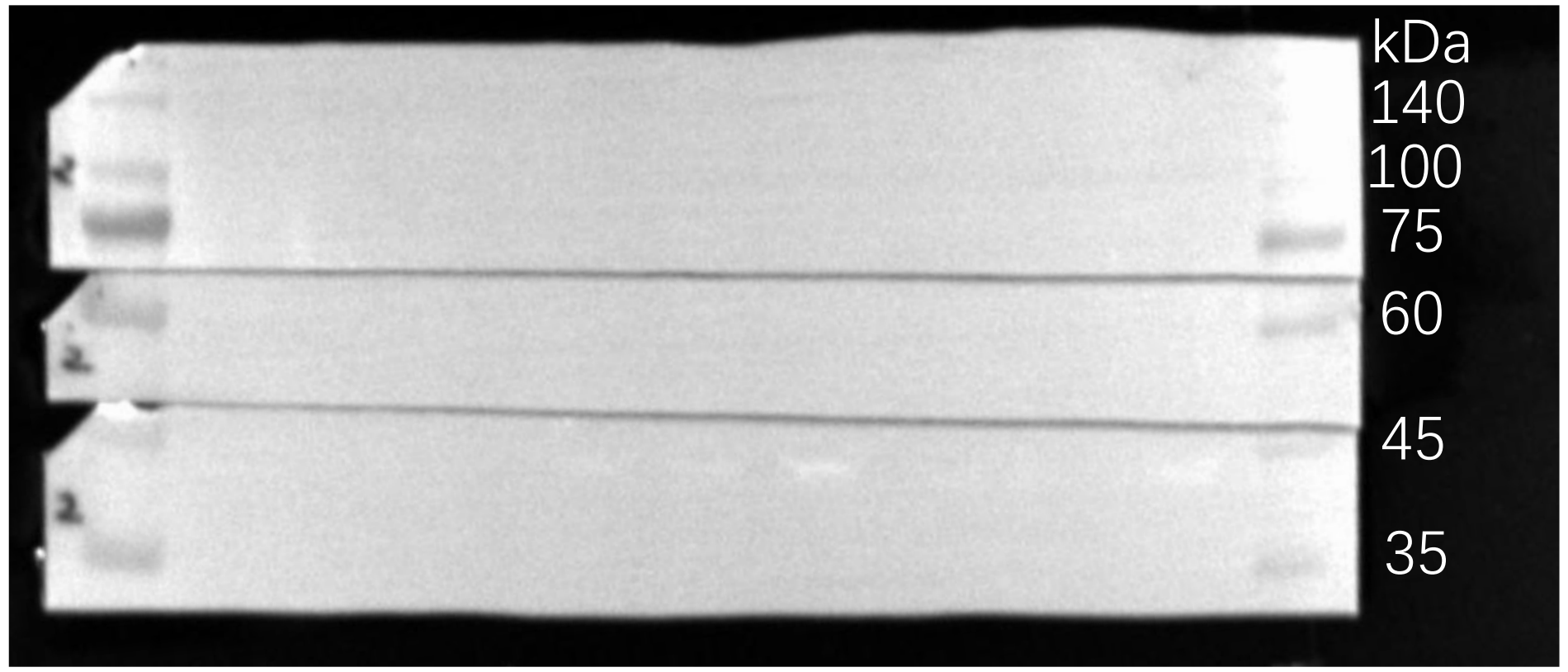

kDa
140
100
75
60
45
35

Figure S5D

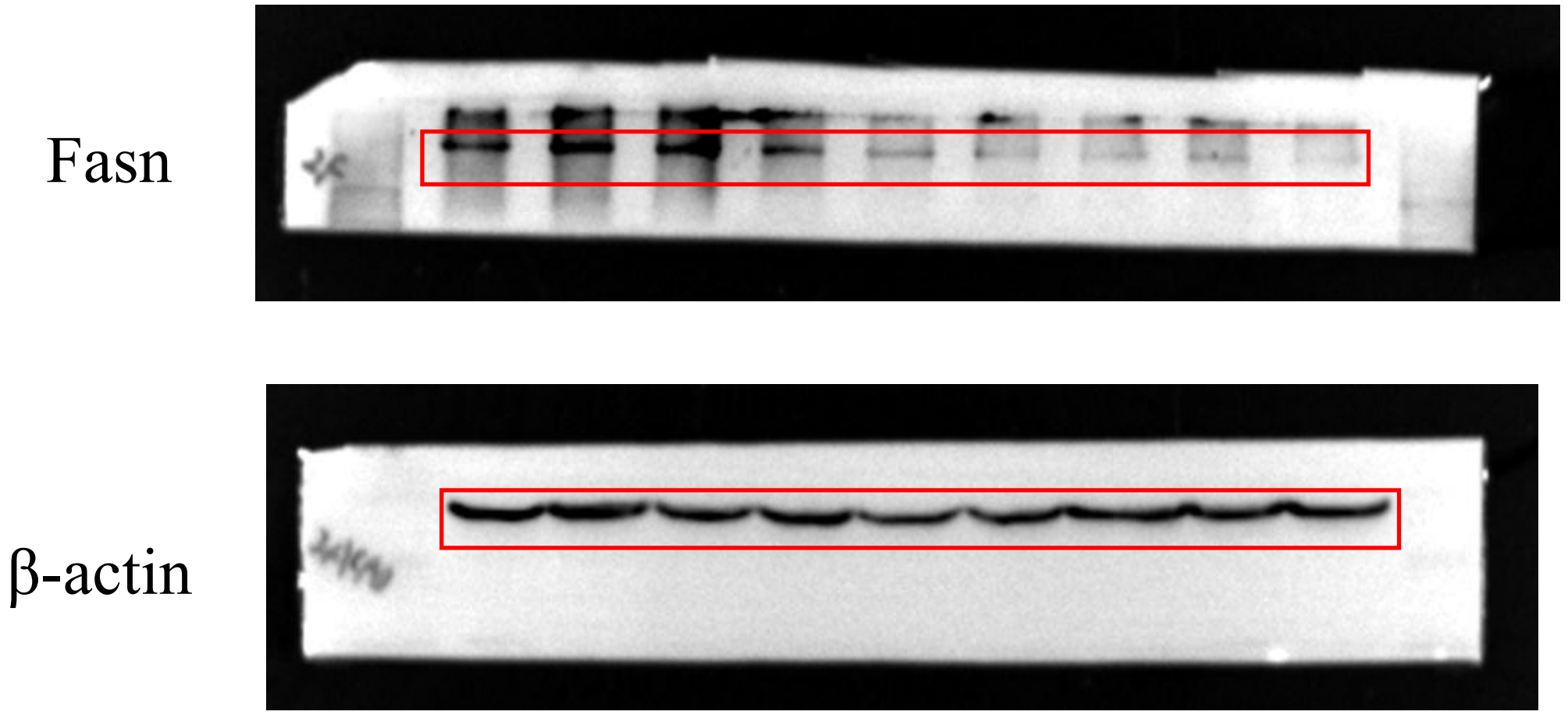
Fasn
β-actin

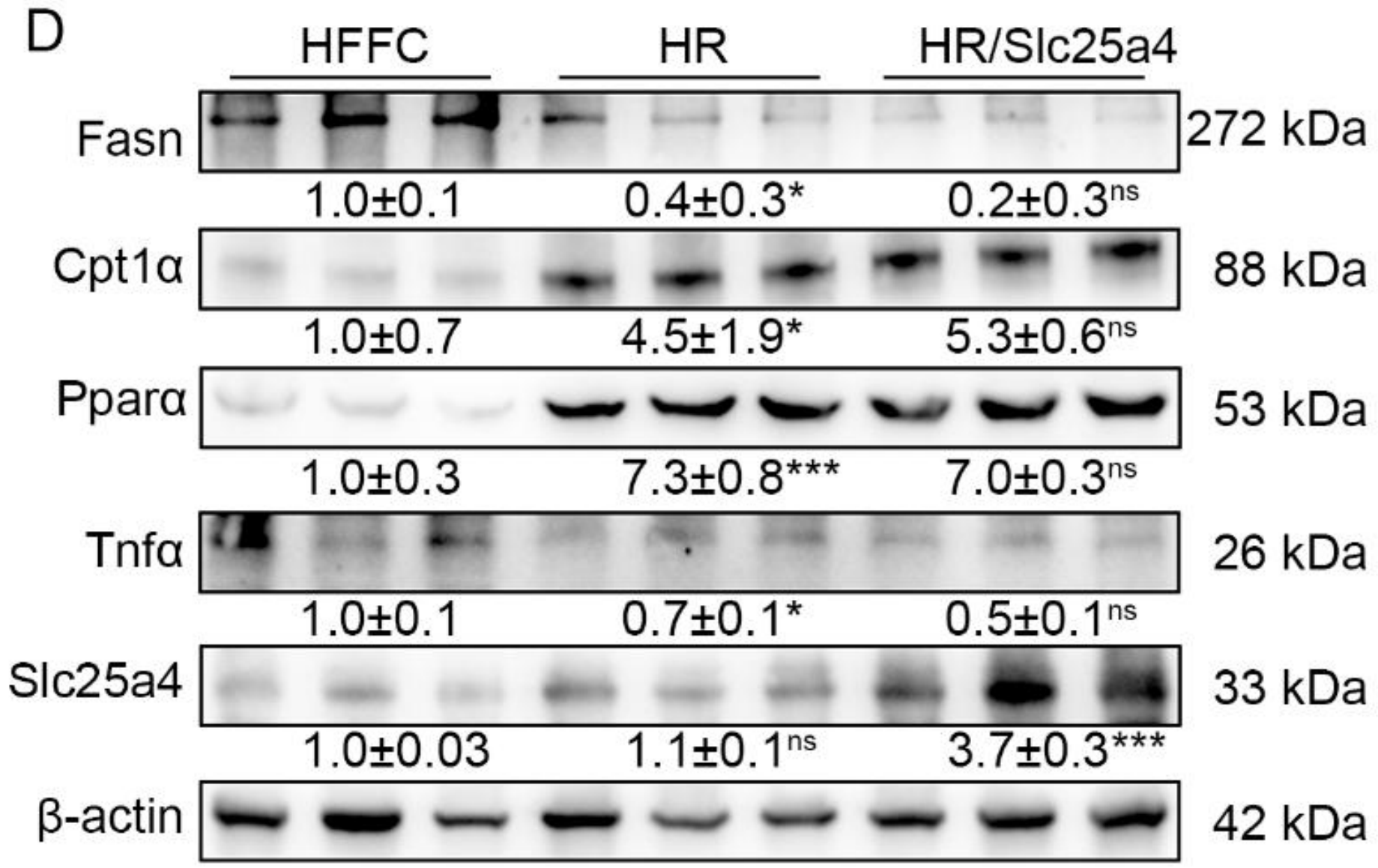
D
HFFC
HR
HR/Slc25a4
Fasn
272 kDa
1.0±0.1
0.4±0.3*
0.2±0.3ns
Cpt1α
88 kDa
1.0±0.7
4.5±1.9*
5.3±0.6ns
Pparα
53 kDa
1.0±0.3
7.3±0.8***
7.0±0.3ns
Tnfα
26 kDa
1.0±0.1
0.7±0.1*
0.5±0.1ns
Slc25a4
33 kDa
1.0±0.03
1.1±0.1ns
3.7±0.3***
β-actin
42 kDa

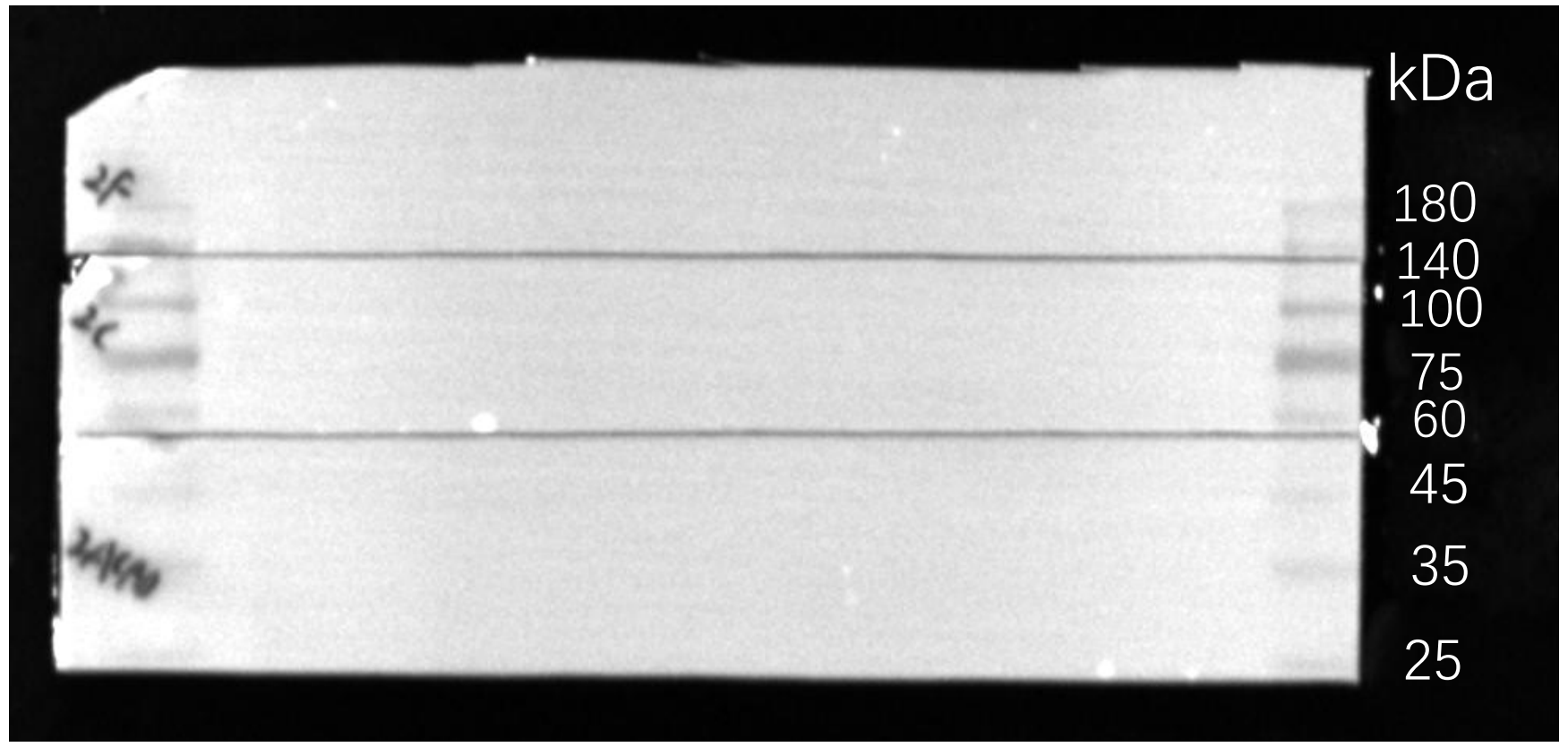
kDa
180
140
100
75
60
45
35
25

Figure S5D

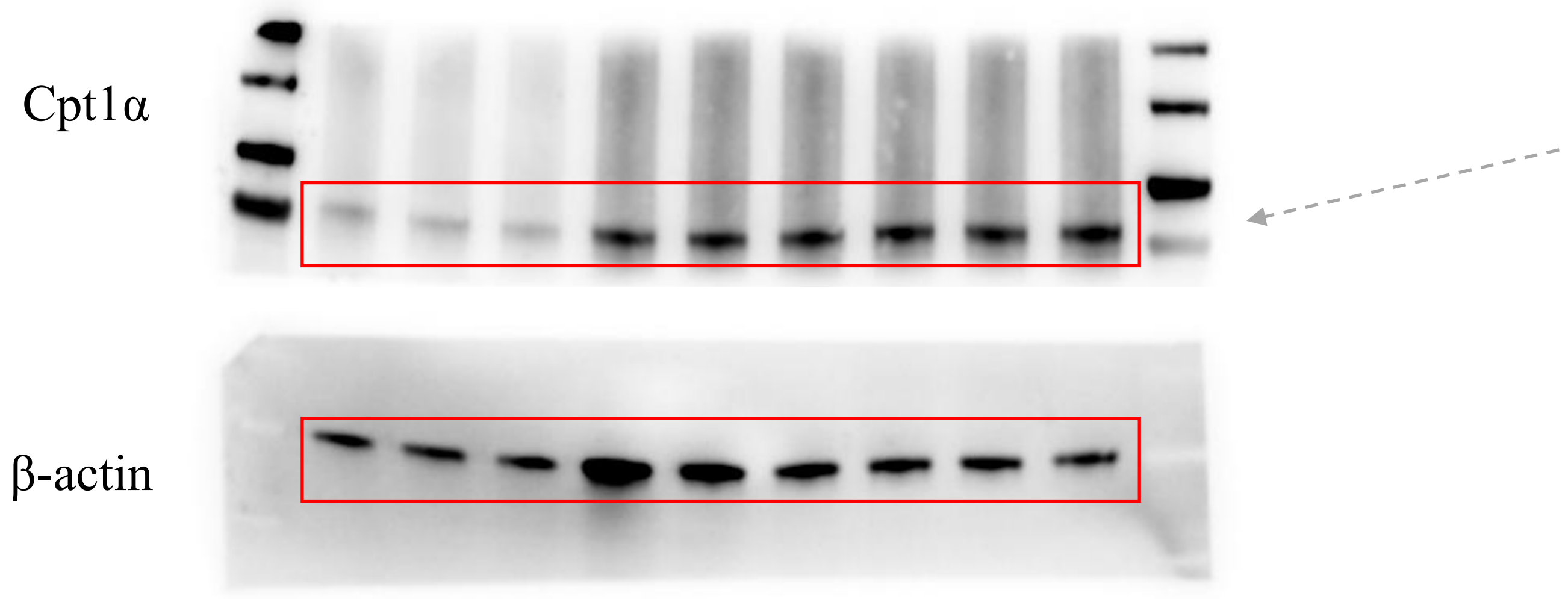
Cpt1α
β-actin

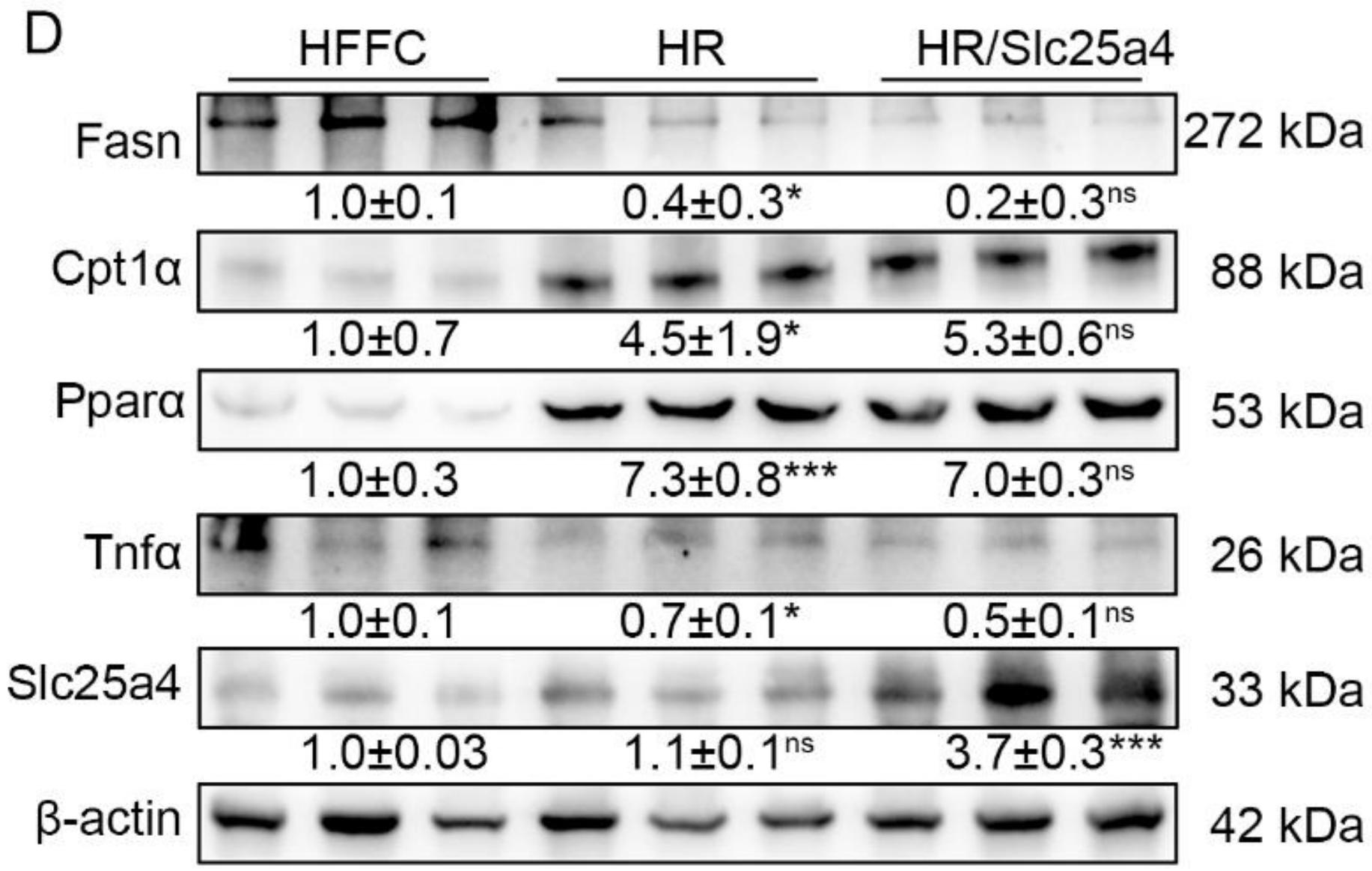
D
HFFC
HR
HR/Slc25a4
Fasn
272 kDa
1.0±0.1
0.4±0.3*
0.2±0.3ns
Cpt1α
88 kDa
1.0±0.7
4.5±1.9*
5.3±0.6ns
Pparα
53 kDa
1.0±0.3
7.3±0.8***
7.0±0.3ns
Tnfα
26 kDa
1.0±0.1
0.7±0.1*
0.5±0.1ns
Slc25a4
33 kDa
1.0±0.03
1.1±0.1ns
3.7±0.3***
β-actin
42 kDa

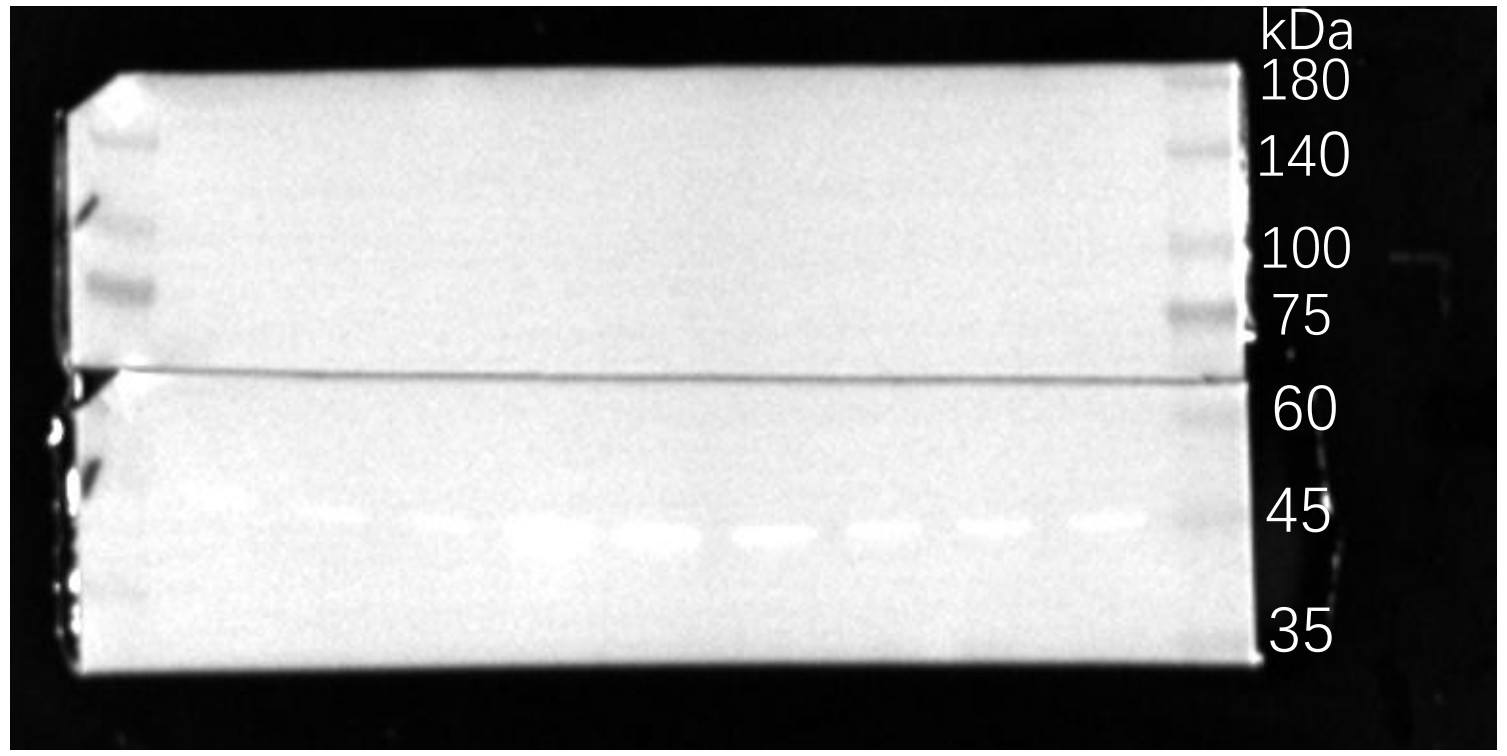
kDa
180
140
100
75
60
45
35

Figure S5D

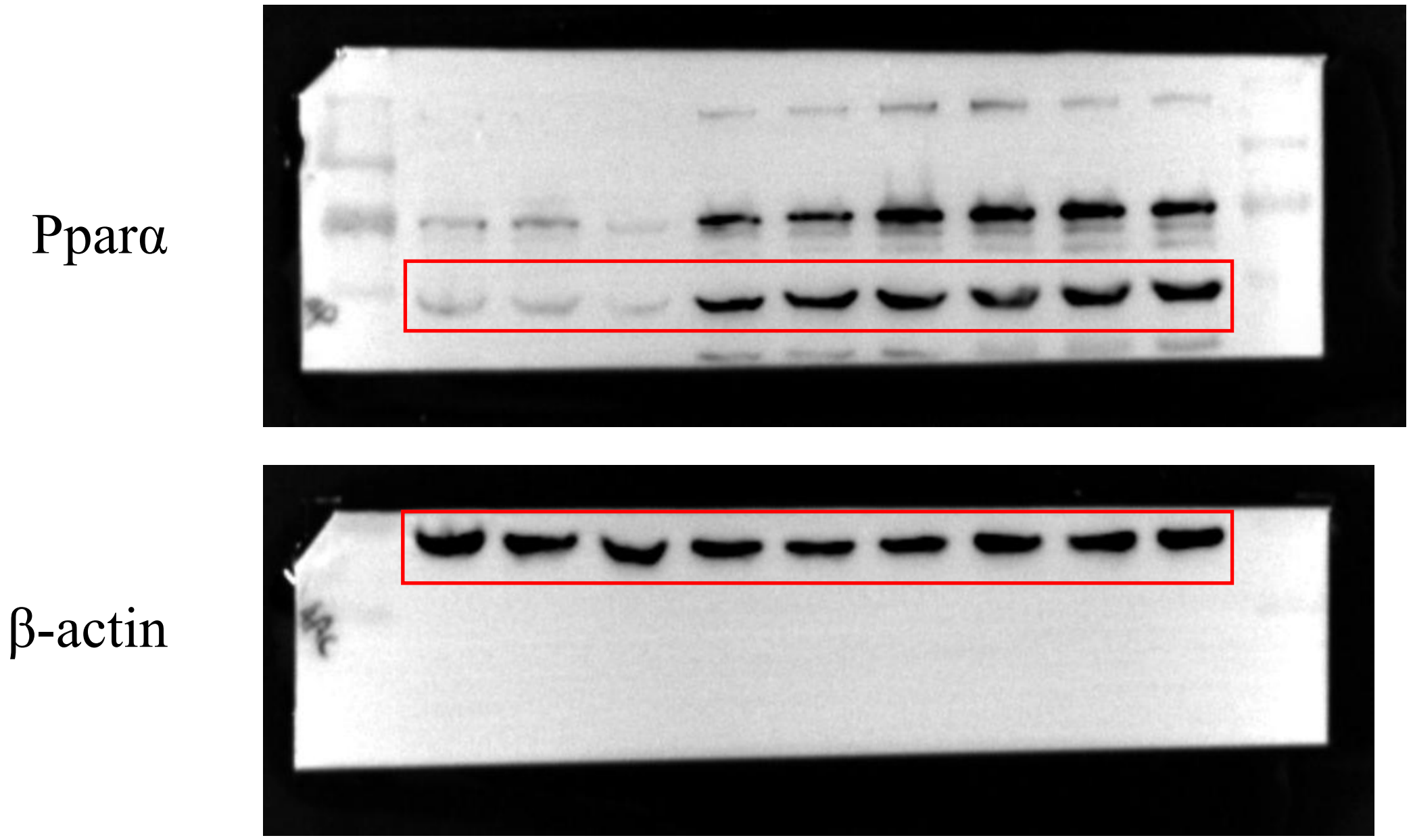
Pparα
β-actin

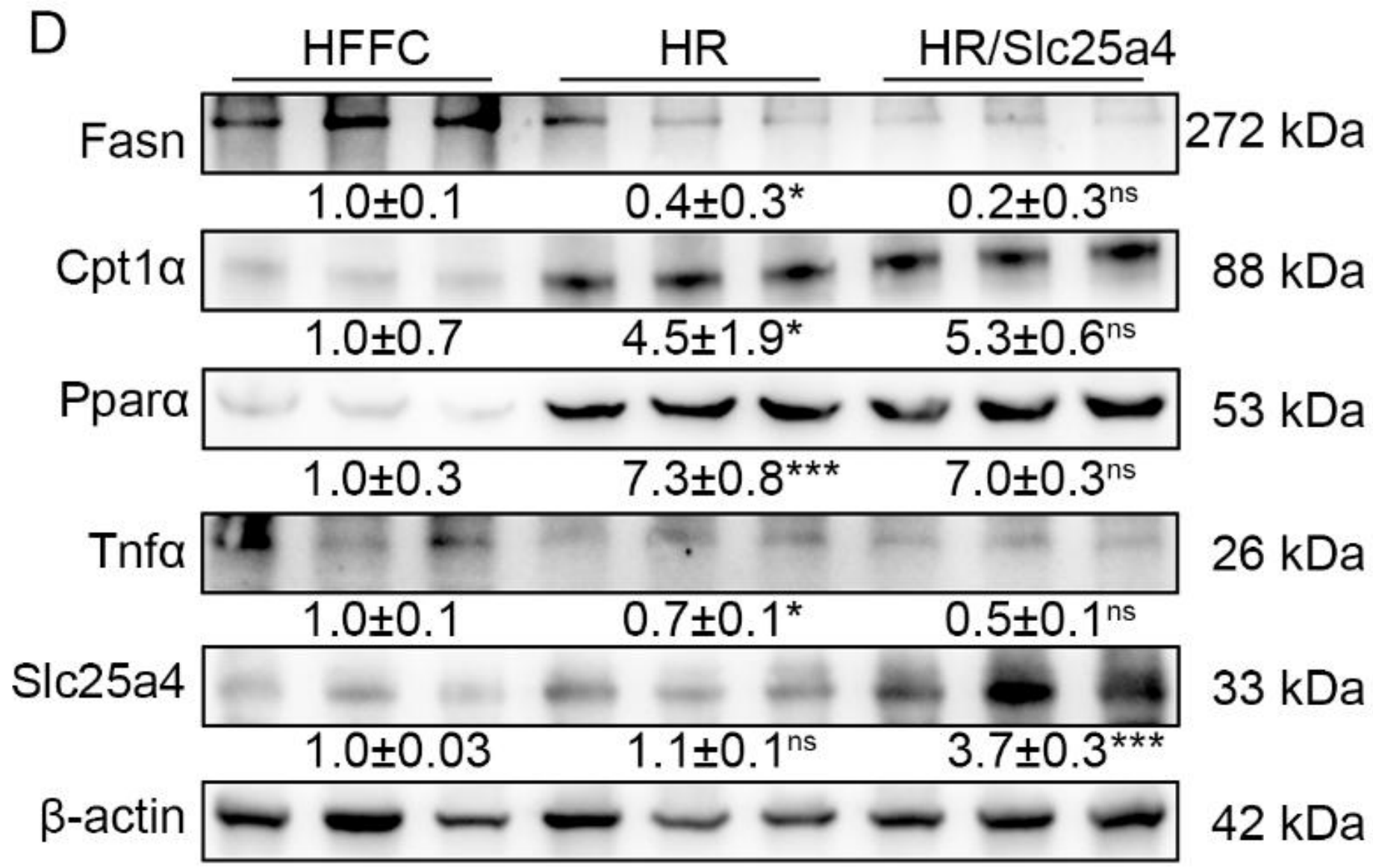
D
HFFC
HR
HR/Slc25a4
Fasn
272 kDa
1.0±0.1
0.4±0.3*
0.2±0.3ns
Cpt1α
88 kDa
1.0±0.7
4.5±1.9*
5.3±0.6ns
Pparα
53 kDa
1.0±0.3
7.3±0.8***
7.0±0.3ns
Tnfα
26 kDa
1.0±0.1
0.7±0.1*
0.5±0.1ns
Slc25a4
33 kDa
1.0±0.03
1.1±0.1ns
3.7±0.3***
β-actin
42 kDa

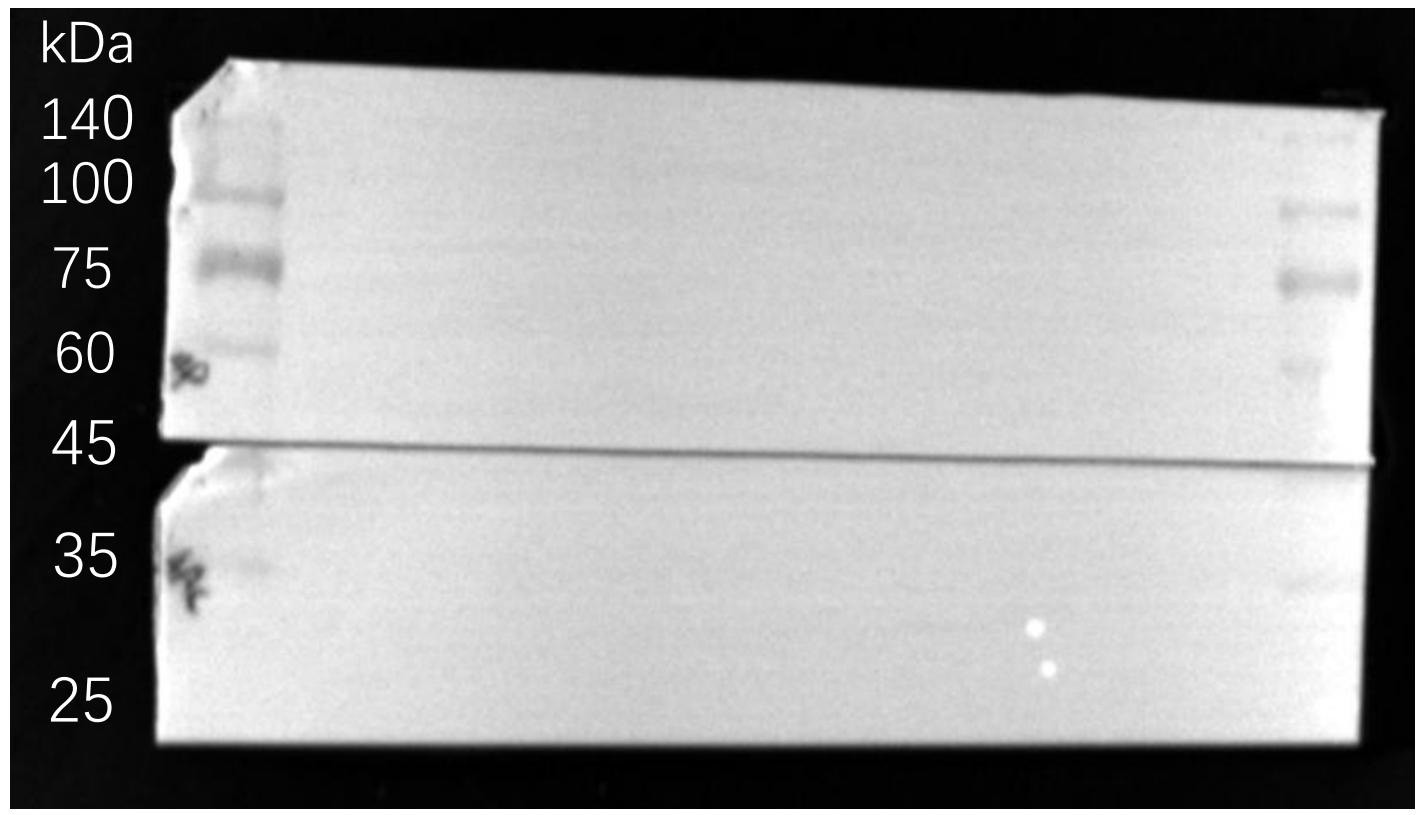
kDa
140
100
75
60
45
35
25

Figure S5D

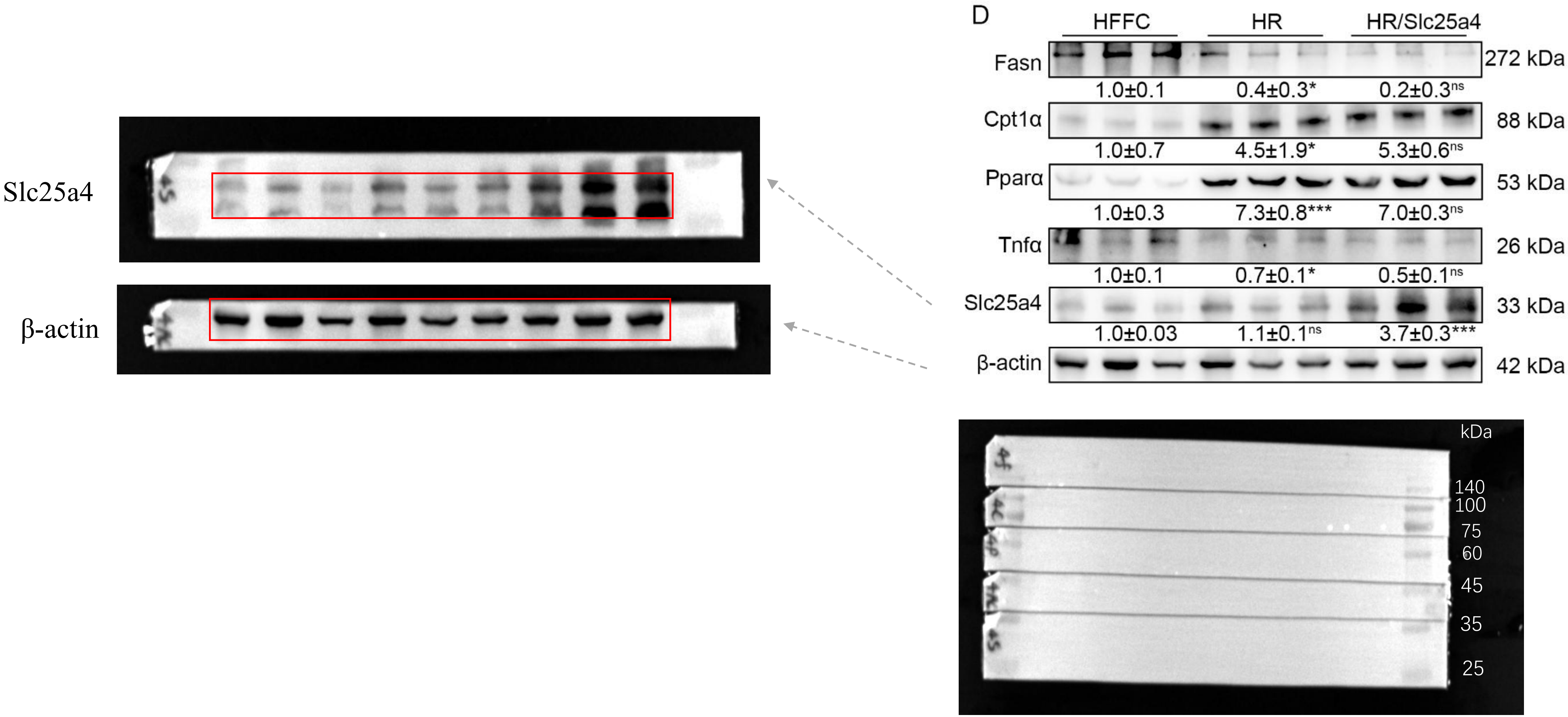
Slc25a4
β-actin
D
HFFC
HR
HR/Slc25a4
Fasn
272 kDa
1.0±0.1
0.4±0.3*
0.2±0.3ns
Cpt1α
88 kDa
1.0±0.7
4.5±1.9*
5.3±0.6ns
Pparα
53 kDa
1.0±0.3
7.3±0.8***
7.0±0.3ns
Tnfα
26 kDa
1.0±0.1
0.7±0.1*
0.5±0.1ns
Slc25a4
33 kDa
1.0±0.03
1.1±0.1ns
3.7±0.3***
β-actin
42 kDa
kDa
140
100
75
60
45
35
25

Figure S5D

Tnfα

β-actin

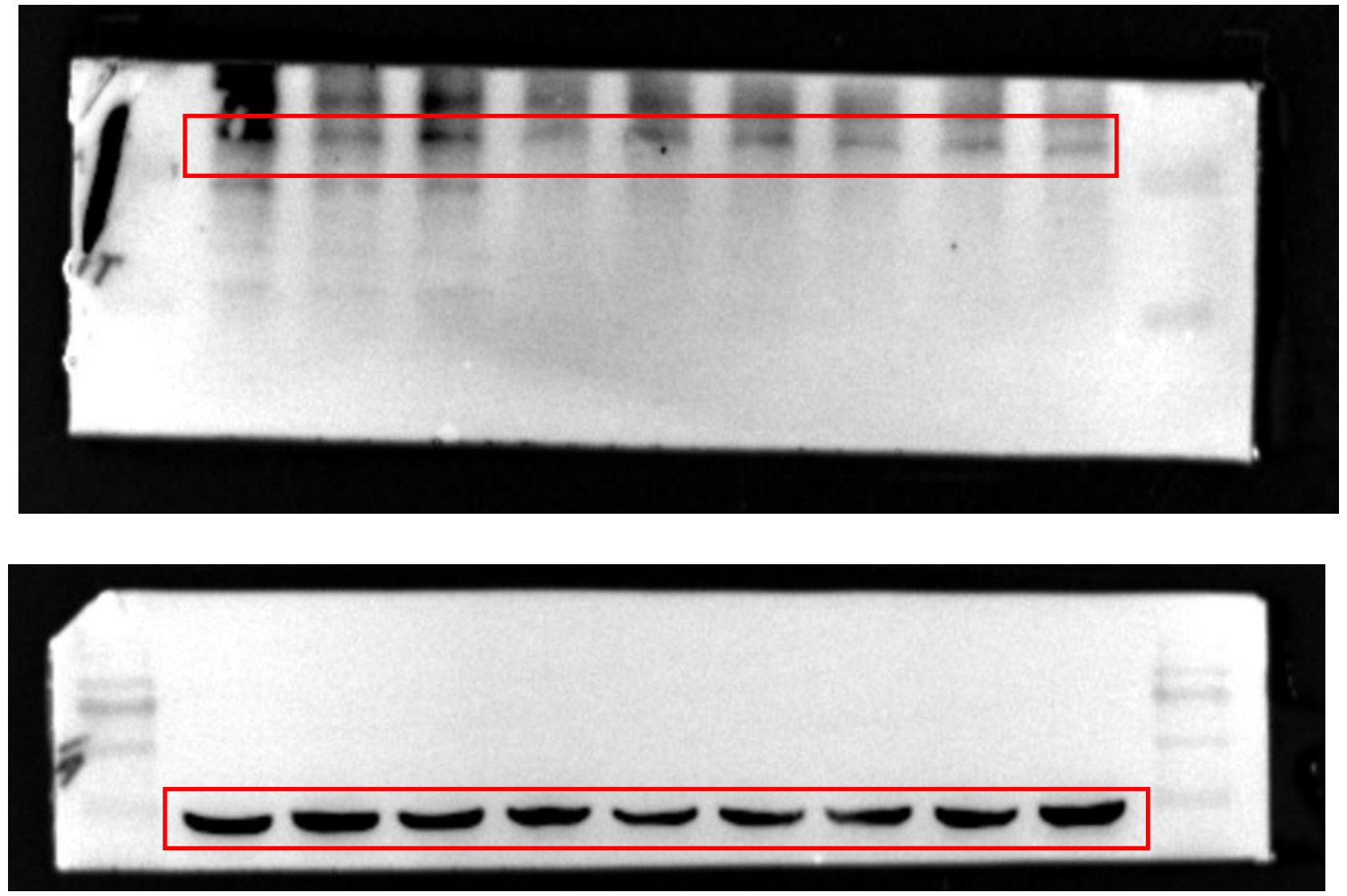

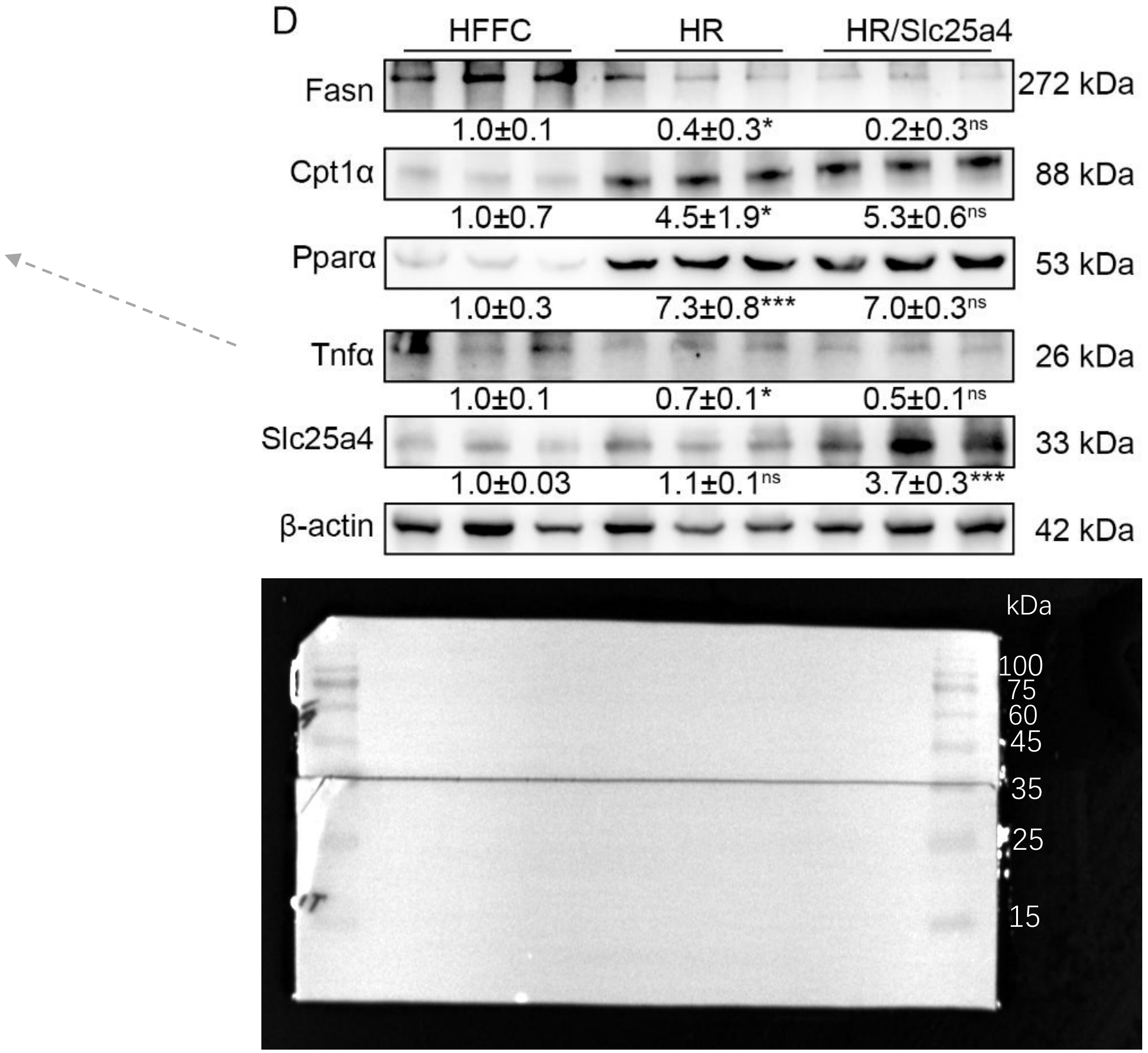

Figure S8D

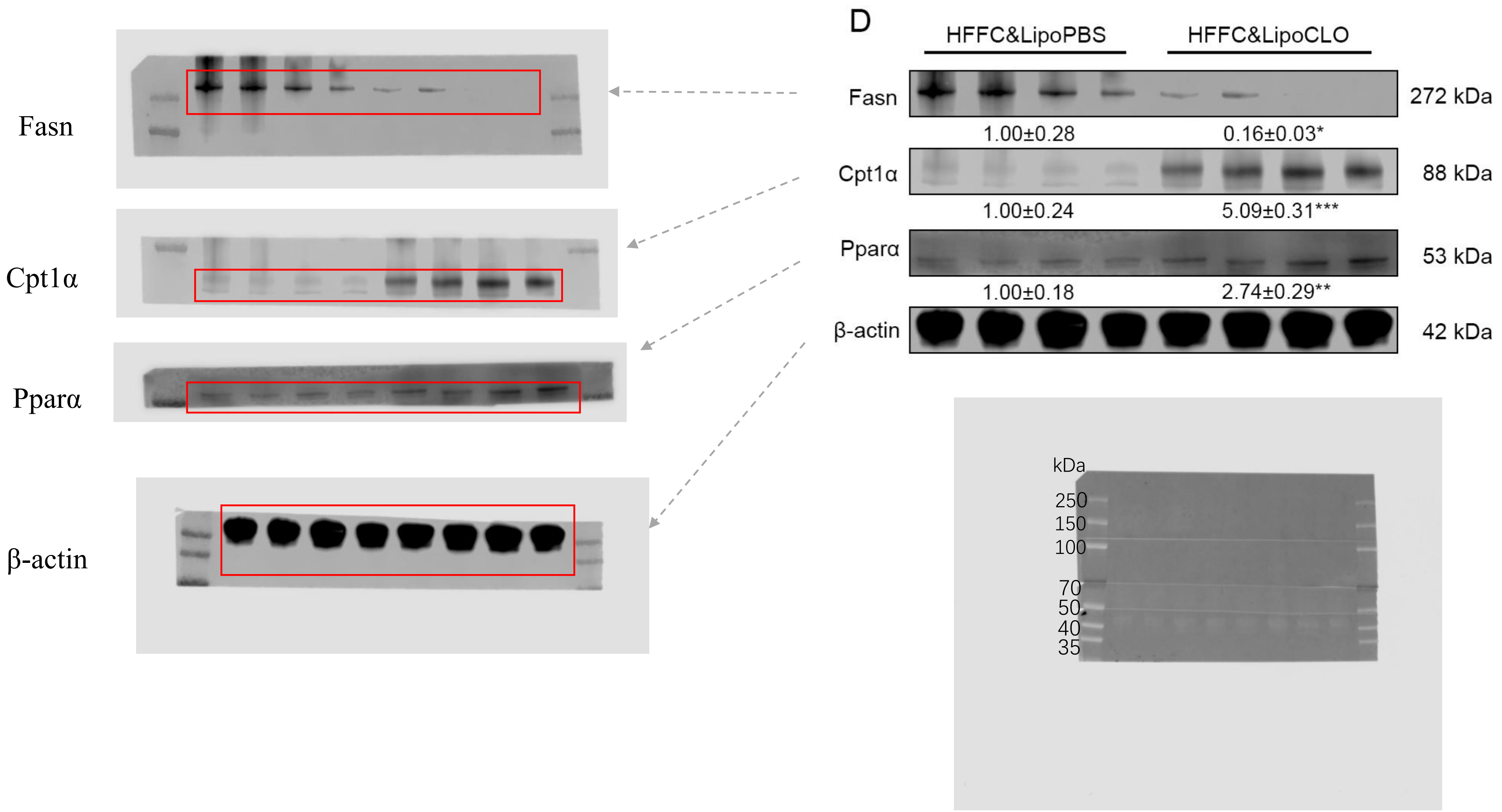
Fasn
Cpt1α
Pparα
β-actin
D
HFFC&LipoPBS
HFFC&LipoCLO
Fasn
272 kDa
1.00±0.28
0.16±0.03*
Cpt1α
88 kDa
1.00±0.24
5.09±0.31***
Pparα
53 kDa
1.00±0.18
2.74±0.29**
β-actin
42 kDa
kDa
250
150
100
70
50
40
35

Figure S11B

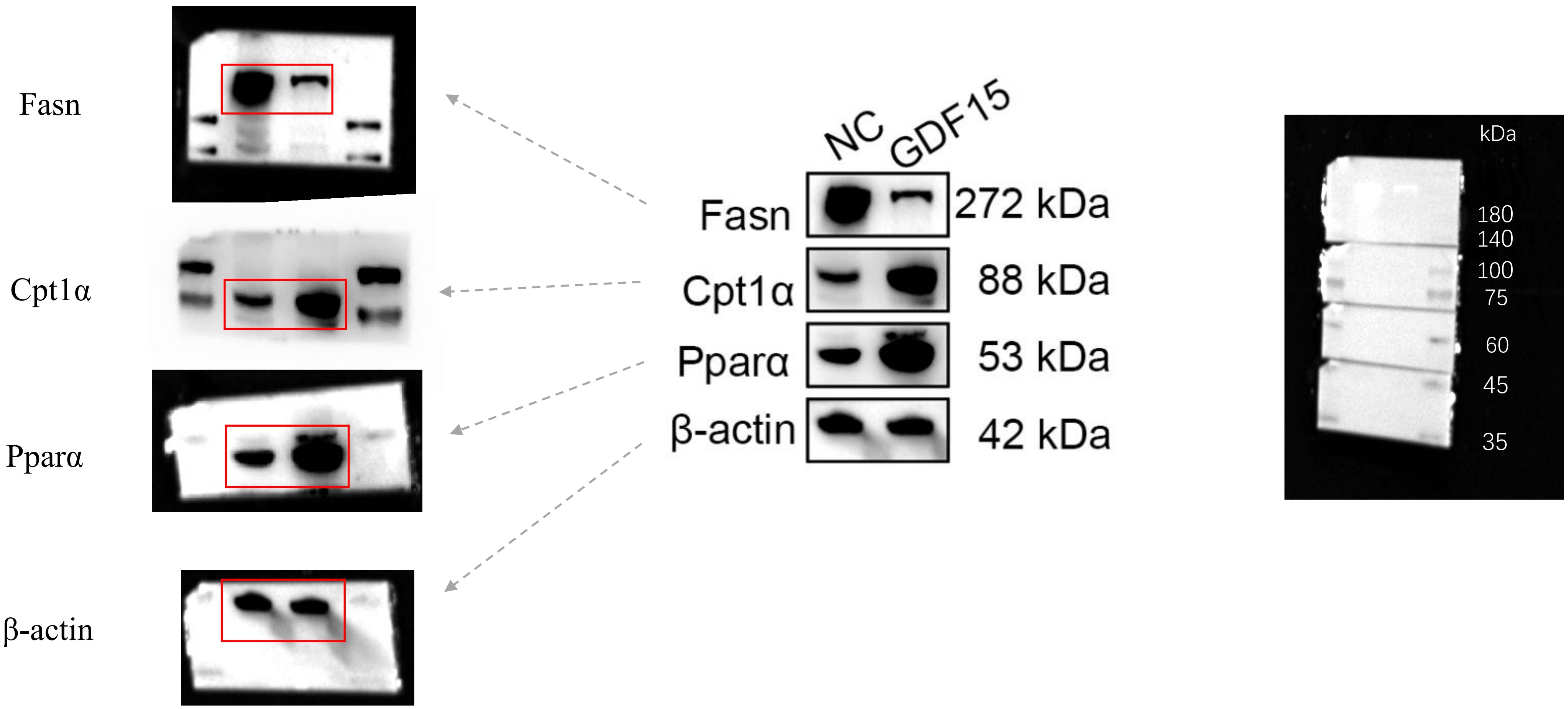
Fasn
Cpt1α
Pparα
β-actin
NC
GDF15
Fasn
Cpt1α
Pparα
β-actin
272 kDa
88 kDa
53 kDa
42 kDa
kDa
180
140
100
75
60
45
35
