# Supplementary material for: SLC25A4high Hepatocytes Enhance Lifestyle Intervention Response in MASLD by Recruiting Eating Macrophages
Source: Adv Sci (Weinh). 2026 Oct 15:e78004. Online ahead of print. doi: 10.1002/advs.78004 (PMC13672689; doi:10.1002/advs.78004)
Supplement: Supplementary file 2 [file ADVS-9999-e78004-s002.docx]

**SLC25A4^high^ Hepatocytes Enhance Lifestyle Intervention Response in MASLD by Recruiting Eating Macrophages**

Authors: Yiting Qian^1,2,^*, Linying Lai^1,6,^*, Yu Zhang^3,^*, Yan Meng^5^, Ziping Song^1^, Baoyuan Huang^1^, Guisheng Song^4,#^, Bo Sun^1,#^, Wenzhuo Yang^1,#^

**Affiliations:**

^1^Department of Gastroenterology and Hepatology, Institute of Digestive Disease, Tongji Hospital, School of Medicine, Tongji University, Shanghai 200065, P.R. China

^2^Department of Clinical Medicine, Suzhou Vocational Health College, Suzhou 215009, China

^3^Department of Gastroenterology, Shanghai East Hospital, School of Medicine, Tongji University, Shanghai 200092, China

^4^Department of Medicine, University of Minnesota Medical School, Minneapolis, Minnesota 55455, United States of America

^5^School of Pharmacy, Hubei University of Chinese Medicine, Wuhan 430065, China

^6^Department of Gastroenterology, Xiang'an Hospital of Xiamen University, School of

Medicine, Xiamen University, Xiamen 361101, China

*Yiting Qian, Linying Lai and Yu Zhang have contributed equally to this work.

**Supplementary result**

**High hepatic SLC25A4 expression is strongly associated with the beneficial effects of lifestyle intervention in MASH.**

To identify key genes mediating the therapeutic effects of lifestyle intervention (LSI), we established a two-stage screening and validation strategy using public transcriptomic datasets (**Supplementary Figure 1**).

First, we utilized the LSI responder/non-responder cohort extracted from the GSE106737 dataset as our training/discovery cohort. This dataset contains transcriptomic profiles of paired liver biopsies from metabolic dysfunction-associated steatohepatitis (MASH)/ nonalcoholic steatohepatitis (NASH) patients before and after intervention, classified into LSI responders and non-responders based on 1-year histological outcomes (**Supplementary Table 2**). Transcriptomic analysis revealed markedly increased immune cell infiltration (predominantly macrophages) and enhanced immunological pathway activation in LSI responders versus non-responders (**Supplementary Figure 2A**). Furthermore, deconvolution of transcriptomic data using CIBERSORT (Cell-type identification by estimating relative subsets of RNA transcripts) (**Supplementary Figure 2B**) and ESTIMATE (estimation of stromal and immune cells in malignant tumor tissues using expression data) algorithms (**Supplementary Figure 2C**), together with gene set enrichment analysis (GSEA) (**Supplementary Figure 2D**), demonstrated markedly increased immune cell infiltration—particularly of macrophages—and enhanced immunological pathway activation in LSI responders compared with LSI non-responders.

We then identified key genes distinguishing these two subsets. At the mRNA level, we performed weighted gene co-expression network analysis (WGCNA) to detect the co-expression modules most strongly positively correlated with macrophage infiltration (turquoise and blue modules; **Supplementary Figure 3A-C**). At the protein level, we applied the Molecular Complex Detection (MCODE) algorithm to identify 12 significant modules (**Supplementary Figure 3D**), among which MCODE4 and MCODE8 exhibited robust performance in the external GSE83452 cohort (area under the curve, AUC 0.948, 0.906) (**Supplementary Figure 3D**). Overlapping WGCNA and MCODE results identified *SLC25A4* and *PSMD8* as hub genes. Consistently, correlation analysis showed that *SLC25A4* and *PSMD8* were positively correlated with M2-like macrophages (**Supplementary Figure 3E**).

Next, to independently confirm our finding, we used the GSE83452 dataset as an external validation cohort exclusively for gene expression verification. *SLC25A4* (encoding the ANT1 protein)—but not *PSMD8*—was significantly upregulated in LSI responders relative to non-responders (**Figure 1A and Supplementary Figure 3F**). Collectively, elevated SLC25A4 expression emerges as a potential mediator of LSI responsiveness, prompting further experimental validation *in vitro* and *in vivo*.

**Supplementary Figure 1** Schematic overview of transcriptomic data mining to identify genes associated with lifestyle intervention responsiveness in MASLD/MASH.

**Supplementary Figure 2 Functional analysis and visualization of Immune microenvironment between LSI responders and non-responders.** (**A**) The top 40 GO term between LSI responders and non-responders. (**B**) Component of immune cells between two groups. (**C**) Histogram of immune scores between the two groups. (**D**) Top 10 gene sets are positively associated with LSI responders based on gene set enrichment analysis. Data represent mean ± standard error of the mean. **P* < 0.05, ***P* < 0.01, and ****P* < 0.001; ns, not significant (B: CIBERSORT analysis; C: ESTIMATE analysis; D: GSEA analysis)

**Supplementary Figure 3 Hub gene signatures associated with differential immune cell phenotypes in exercise responders and non-responders.** (**A**) Two distinct modules of genes in exercise responders and non-responders, as revealed by hierarchical clustering. (**B**) Heatmap showing correlations between module eigengenes (MEs) and immune cell populations. (**C**) Correlation between gene significance (GS) for macrophage infiltration and module membership (MM) in the turquoise and blue modules. (**D**) Receiver Operating Characteristic (ROC) curve analysis of the predictive performance of hub genes from the top-scoring Molecular Complex Detection (MCODE) cluster. (**E**) Independent validation of correlations between hub genes and immune cells. (**F**) Expression levels of *PSMD8* in LSI responders and non-responders in the training and validation cohorts. Data represent mean ± standard error of the mean. **P* < 0.05, ***P* < 0.01, and ****P* < 0.001; ns, not significant. LSI: lifestyle intervention. (B: WGCNA analysis; C, E: Pearson's correlation analysis; D: Logistic regression; F: two-tailed Student's t-test).

**Supplementary Figure 4 Correlation analysis between Slc25a4 and rate of controlled attenuation parameter (CAP) change.** (**A**) Correlation Analysis: Baseline Slc25a4 vs. rate of CAP change. Data represent mean ± standard error of the mean. **P* < 0.05, ***P* < 0.01, and ****P* < 0.001; ns, not significant (A: Pearson's correlation analysis)

**Supplementary Figure 5 Liver-specific expression of *Slc25a4* fails to enhance dietary restriction-mediated amelioration on MASLD.** (**A**) Schematic diagram: *Slc25a4*-overexpressing mice were fed a HFFC diet for 16 weeks, followed by a 4-week intervention of diet restriction. (**B**) Macroscopic and microscopic (H&E, original magnification, ×200) appearance of livers from HFFC mice, HR mice and HR/Slc25a4 mice. HFFC: high-fat, high-fructose, and high-cholesterol diet; HR: HFFC following diet restriction; H&E: Hematoxylin and Eosin. L.W., liver weight. (**C**) MASH activity score (MAS) in the livers of 3 groups of mice. (**D**) Protein levels of Fasn, Cpt1α, Pparα, Tnfα and Slc25a4 in livers of 3 groups of mice. (**E**) mRNA levels of *Il1β, Tnfα, Il6, Il10* in livers of 3 groups of mice. Data represent mean ± standard error of the mean. **P* < 0.05, ***P* < 0.01, and ****P* < 0.001; ns, not significant (C-E: one-way ANOVA followed by Tukey's post hoc test). Schematic diagram was created with BioRender.com. Original blots can be found in Supplementary Information.

**Supplementary Figure 6 UMAP feature plots of macrophage marker expression.** (**A**) UMAP visualization showing the expression distribution of macrophage marker genes *Cd163* and *Ccr2*.

**Supplementary Figure 7 Exhaustion of KCs during MASLD development.** (**A**) Immunostaining and flow cytometry analyses of livers of three groups of mice. The arrow represents Clec4f^+^ cells. Data represent mean ± standard error of the mean. **P* < 0.05, ***P* < 0.01, and ****P* < 0.001; ns, not significant (A: one-way ANOVA followed by Tukey's post hoc test)

**Supplementary Figure 8 Macrophage depletion alleviates hepatic steatosis and inflammation in HFFC mice. (A)** Macroscopic and microscopic (H&E, original magnification, ×200) appearance of livers from HFFC & LipoPBS mice (n = 6) and HFFC & LipoCLO mice (n = 6). Immunostaining of F4/80 marker in livers of two groups of mice. L.W., liver weight. **(B)** MASH activity score (MAS) in livers of two groups of mice. (C) Ratios of F4/80⁺ cells in livers of two groups of mice. (**D**) Protein levels of Fasn, Cpt1α and Pparα in livers of two groups of mice. (**E**) mRNA levels of genes encoding *Fasn*, *Pparα*, *Cpt1α*, *Il1β* and *Tnfα* in livers of two groups of mice. HFFC: high-fat, high-fructose, and high-cholesterol diet; LipoPBS: Control Liposomes (PBS); LipoCLO: Clodronate Liposomes; H&E: Hematoxylin and Eosin. Data represent mean ± standard error of the mean. **P* < 0.05, ***P* < 0.01, and ****P* < 0.001; ns, not significant (B, C, D and E: two-tailed Student's t-test). Original blots can be found in Supplementary Information.

**Supplementary Figure 9** **Free fatty acid stimulation promoted M1-like polarization of monocytes treated with M-CSF.** (**A**) mRNA levels of *Tnfα*, *Il6*, *Ptgs2*, *Ccl5*, *Mgl1*, *Mgl2*, *Il10* and *Arg1* in M-CSF-treated monocytes with/without FFA stimulation. FFA: free fatty acid. Data represent mean ± standard error of the mean. **P* < 0.05, ***P* < 0.01, and ****P* < 0.001; ns, not significant (A: two-tailed Student's t-test). Schematic diagrams were created with BioRender.com.

**Supplementary Figure 10 Functional pathway enrichment analysis across different macrophage subsets.** (A) The distinct pathways in Clec4f^+^Timd4^-^, Adgre1^+^Ly6c^-^, or Ly6c^+^Ccr2^+^ macrophages.

**Supplementary Figure 11 Co-culture of GDF15-expressing macrophages inhibited expression of lipogenic *Fasn* and promoted expression of FAO-related genes in hepatocytes.** (A) Lipid quantification in AML12 cells co-cultured with GDF15-expressing RAW264.7 via flow cytometry. (B) Protein levels of Fasn, Cpt1α and Pparα in THLE-2 cells stimulated with recombinant GDF15 protein. Data represent mean ± standard error of the mean. **P* < 0.05, ***P* < 0.01, and ****P* < 0.001; ns, not significant (A-B: two-tailed Student's t-test). Schematic diagrams were created with BioRender.com. Original blots can be found in Supplementary Information.

**Supplementary Figure 12** **GDF15-PPARα signaling is required for Slc25a4 to reduce hepatic lipid content**. (**A**) TG content in the livers of HFFCOE/SCR (*n* = 6), HEOE/SCR (*n* = 6) or HEOE/shGDF15 (*n* = 6). (**B**) MASH activity score in the livers of HFFCOE/SCR (*n* = 6), HEOE/SCR (*n* = 6) and HEOE/shPPARα (*n* = 6). HFFC: high-fat, high-fructose, and high-cholesterol diet; HFFCOE: HFFC-fed mice injected with Slc25a4; HEOE: HFFC-fed, exercise-trained mice with *Slc25a4* overexpression. SCR: scramble. Data represent mean ± standard error of the mean. **P* < 0.05, ***P* < 0.01, and ****P* < 0.001; ns, not significant (A-B: one-way ANOVA followed by Tukey's post hoc test)

**Supplementary Table 1**

| Gene | Species | Primer sequence (5' to 3') | |
| --- | --- | --- | --- |
|  |  | Forward Primer | Reverse Primer |
| *Slc25a4* | mouse | AACCTGGCCAACGTGATCC | CTCGCTGGGAAGATCCCTTG |
| *Fasn* | mouse | GGAGGTGGTGATAGCCGGTAT | TGGGTAATCCATAGAGCCCAG |
| *Cpt1α* | mouse | TTTGCCTCGTGTTTGTGGG | CAGCCGTGGTAGGACAGAA |
| *Acadm* | mouse | AGGGTTTAGTTTTGAGTTGACGG | CCCCGCTTTTGTCATATTCCG |
| *Tnfα* | mouse | CAGGCGGTGCCTATGTCTC | CGATCACCCCGAAGTTCAGTAG |
| *Il1β* | mouse | GCAACTGTTCCTGAACTCAACT | ATCTTTTGGGGTCCGTCAACT |
| *Il6* | mouse | ACAAGTCGGAGGCTTAATTACACAT | TTGCCATTGCACAACTCTTTTC |
| *Il10* | mouse | CTGGCTCAGCACTGCTATGT | GCAGTTATTGTCACCCCGGA |
| *Ccl5* | mouse | GCTGCTTTGCCTACCTCTCC | TCGAGTGACAAACACGACTGC |
| *Mgl1* | mouse | GCCTGGTAAAGCAGCTCCTTC | CACATCCACTTTCAGAGAACTGAT |
| *Mgl2* | mouse | TTAGCCAATGTGCTTAGCTGG | GGCCTCCAATTCTTGAAACCT |
| *Arg1* | mouse | CTCCAAGCCAAAGTCCTTAGAG | AGGAGCTGTCATTAGGGACATC |
| *Pparα* | mouse | GGCCTTGACCTTGTTCATGT | ATGCCAGTACTGCCGTTTTC |
| *Pbx1* | mouse | AAGCCTGCCTTGTTTAATGTGT | GCCCAAAGGTTGGAGTTTTCA |
| *Nr4a1* | mouse | TTGAGTTCGGCAAGCCTACC | GTGTACCCGTCCATGAAGGTG |
| *Areg* | mouse | GGTCTTAGGCTCAGGCCATTA | CGCTTATGGTGGAAACCTCTC |
| *Ptgs2* | mouse | TTCAACACACTCTATCACTGGC | AGAAGCGTTTGCGGTACTCAT |
| *P2ry12* | mouse | ATGGATATGCCTGGTGTCAACA | AGCAATGGGAAGAGAACCTGG |
| *β-actin* | mouse | GGCTGTATTCCCCTCCATCG | CCAGTTGGTAACAATGCCATGT |

**Supplementary Table 2. Clinical and histological characteristics of LSI responders and non-responders before and after lifestyle intervention**

| Parameter | LSI responders (n=10) | | | | LSI non-responders (n=10) | | | | |
| --- | --- | --- | --- | --- | --- | --- | --- | --- | --- |
|  | Baseline | After LSI | P value | | | Baseline | After LSI | | P value |
| Age (years) | 45.0 ± 13.9 |  | |  | | 45.7 ± 14.2 | |  |  |
| BMI (kg/m²) | 40.3 ± 11.2 | 37.0 ± 10.5 | | 0.002 | | 38.0 ± 5.5 | | 34.7 ± 5.5 | 0.01 |
| Body weight (kg) | 113.9 ± 26.2 | 104.6 ± 24.6 | | 0.001 | | 112.2 ± 21.2 | | 102.2 ± 18.4 | 0.04 |
| Lobular inflammation (score) | 1.6 ± 0.8 | 0.5 ± 0.7 | | 0.01 | | 1.6 ± 0.8 | | 1.7 ± 0.5 | NS |
| Hepatocellular ballooning (score) | 1.7 ± 0.5 | 0.5 ± 0.5 | | 0.003 | | 1.4 ± 0.5 | | 1.6 ± 0.5 | NS |
| Steatosis (score) | 1.6 ± 0.8 | 0.7 ± 0.8 | | 0.058 | | 2.5 ± 0.7 | | 1.9 ± 0.6 | NS |
| NAFLD Activity Score (NAS) | 4.9 ± 1.4 | 1.7 ± 1.9 | | 0.006 | | 5.5 ± 1.2 | | 5.2 ± 1.1 | NS |
| Fasting glucose (mg/dl) | 83.2 ± 17.9 | 84.2 ± 13.7 | | NS | | 80.1 ± 11.3 | | 85.7 ± 6.6 | NS |
| 2-hour postprandial glucose (mg/dl) | 153.5 ± 45.5 | 120.3 ± 41.4 | | 0.002 | | 130.2 ± 19.1 | | 131.0 ± 30.0 | NS |

Note: In LSI non-responders, although body weight improved significantly after lifestyle intervention, histological features including lobular inflammation, hepatocellular ballooning, and steatosis remained unchanged. Data are presented as mean ± SD. LSI, lifestyle intervention; NS, not significant. (Adapted from PMID: 31701087, data access was authorized by the Springer Nature, permit no.: 6322300929277)
